# Supplementary material for: Association analyses of host genetics, root-colonizing microbes, and plant phenotypes under different nitrogen conditions in maize
Source: eLife. 2022 Jul 27;11:e75790. doi: 10.7554/eLife.75790 (PMC9470161; doi:10.7554/eLife.75790)
Supplement: Supplementary file 6. [file elife-75790-supp6.pdf]

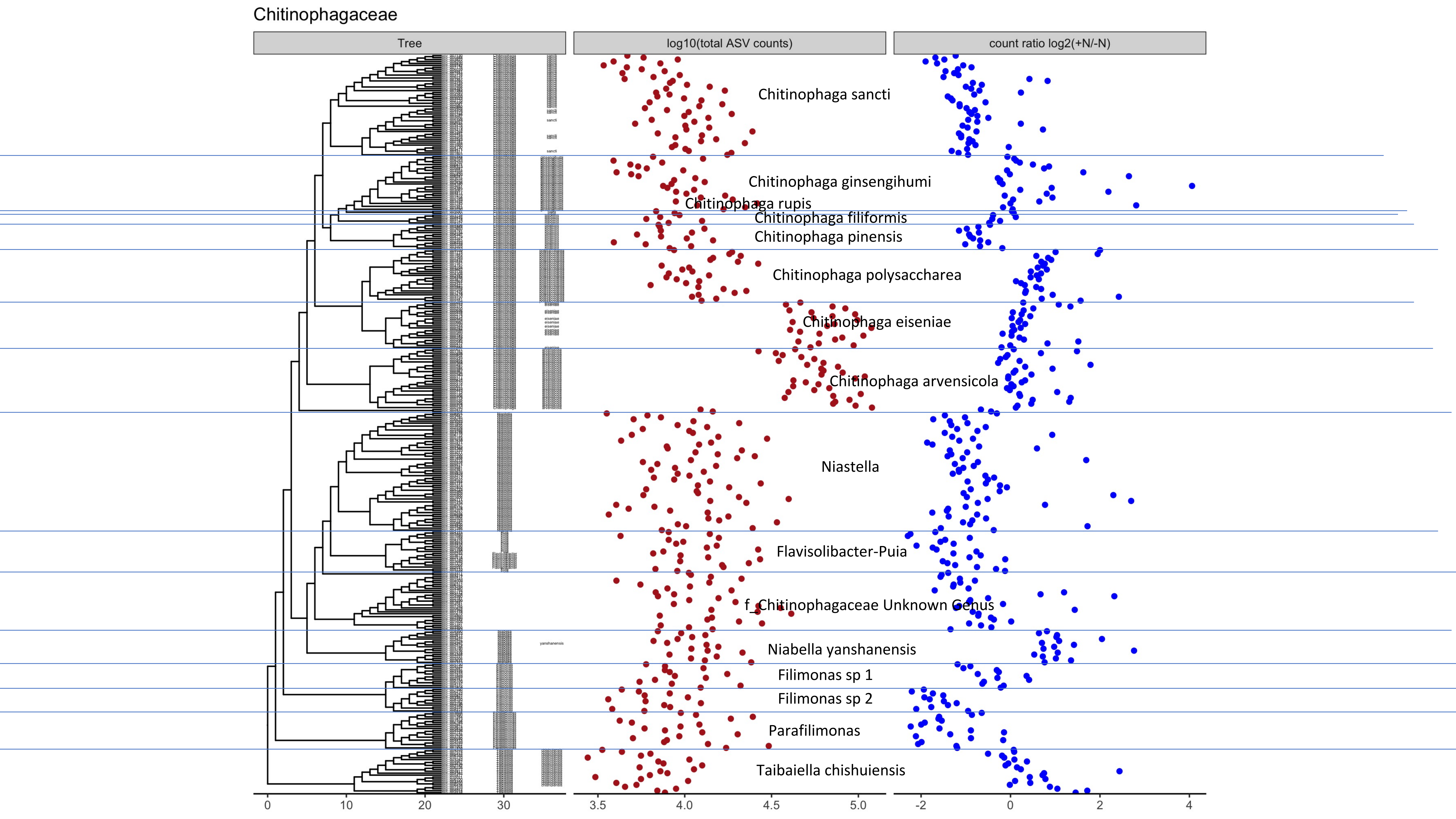

Chitinophagaceae
Tree
log10(total ASV counts)
count ratio log2(+N/-N)
Chitinophaga sancti
Chitinophaga ginsengihumi
Chitinophaga rupis
Chitinophaga filiformis
Chitinophaga pinensis
Chitinophaga polysaccharea
Chitinophaga eiseniae
Chitinophaga arvensicola
Niastella
Flavisolibacter-Puia
f_Chitinophagaceae Unknown Genus
Niabella yanshanensis
Filimonas sp 1
Filimonas sp 2
Parafilimonas
Taibaiella chishuiensis
0
10
20
30
3.5
4.0
4.5
5.0
-2
0
2
4

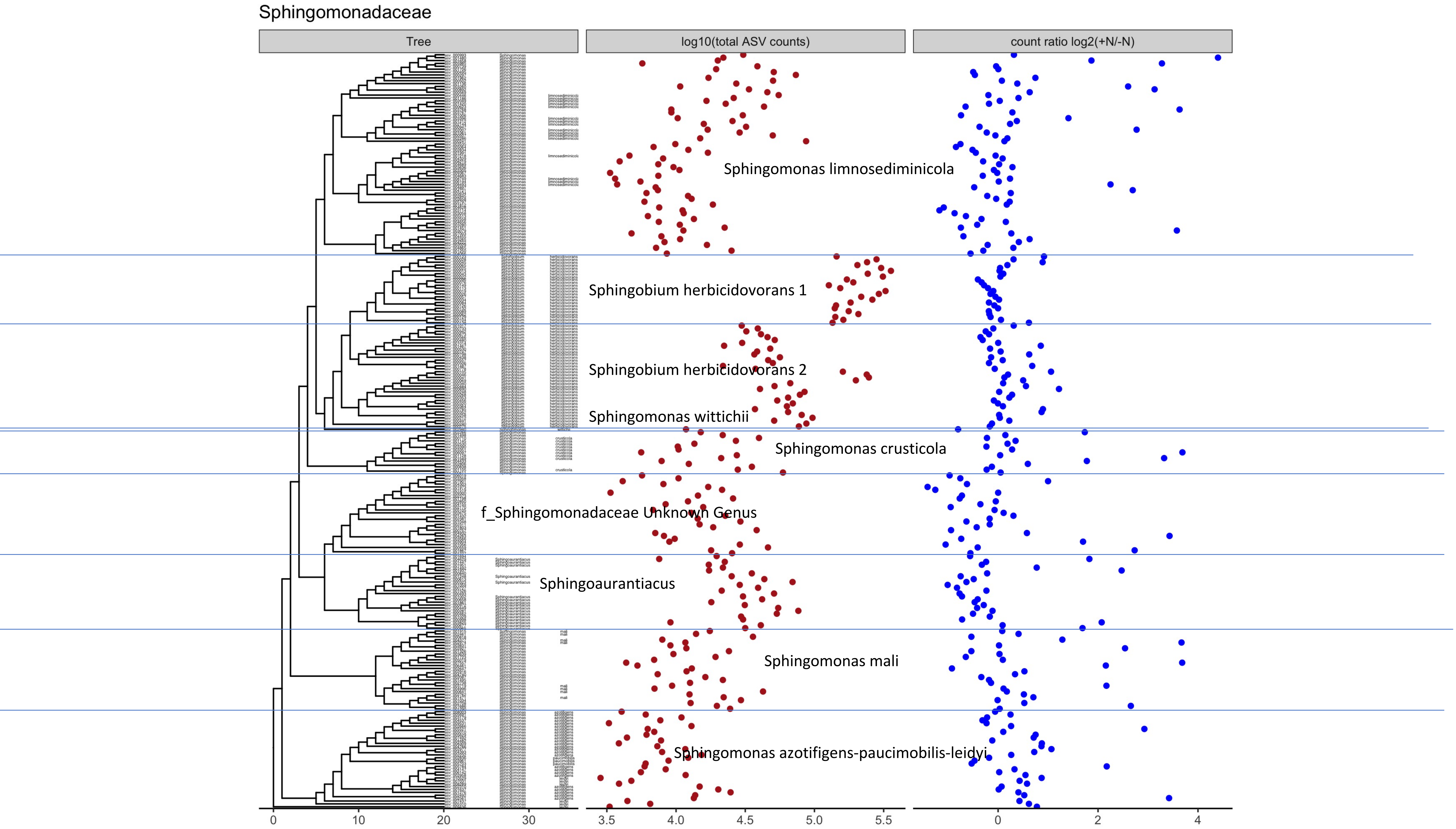
Sphingomonadaceae
Tree
log10(total ASV counts)
count ratio log2(+N/-N)
Sphingomonas limnosediminicola
Sphingobium herbicidovorans 1
Sphingobium herbicidovorans 2
Sphingomonas wittichii
Sphingomonas crusticola
f_Sphingomonadaceae Unknown Genus
Sphingoaurantiacus
Sphingomonas mali
Sphingomonas azotifigens-paucimobilis-leidyi
0
10
20
30
3.5
4.0
4.5
5.0
5.5
0
2
4

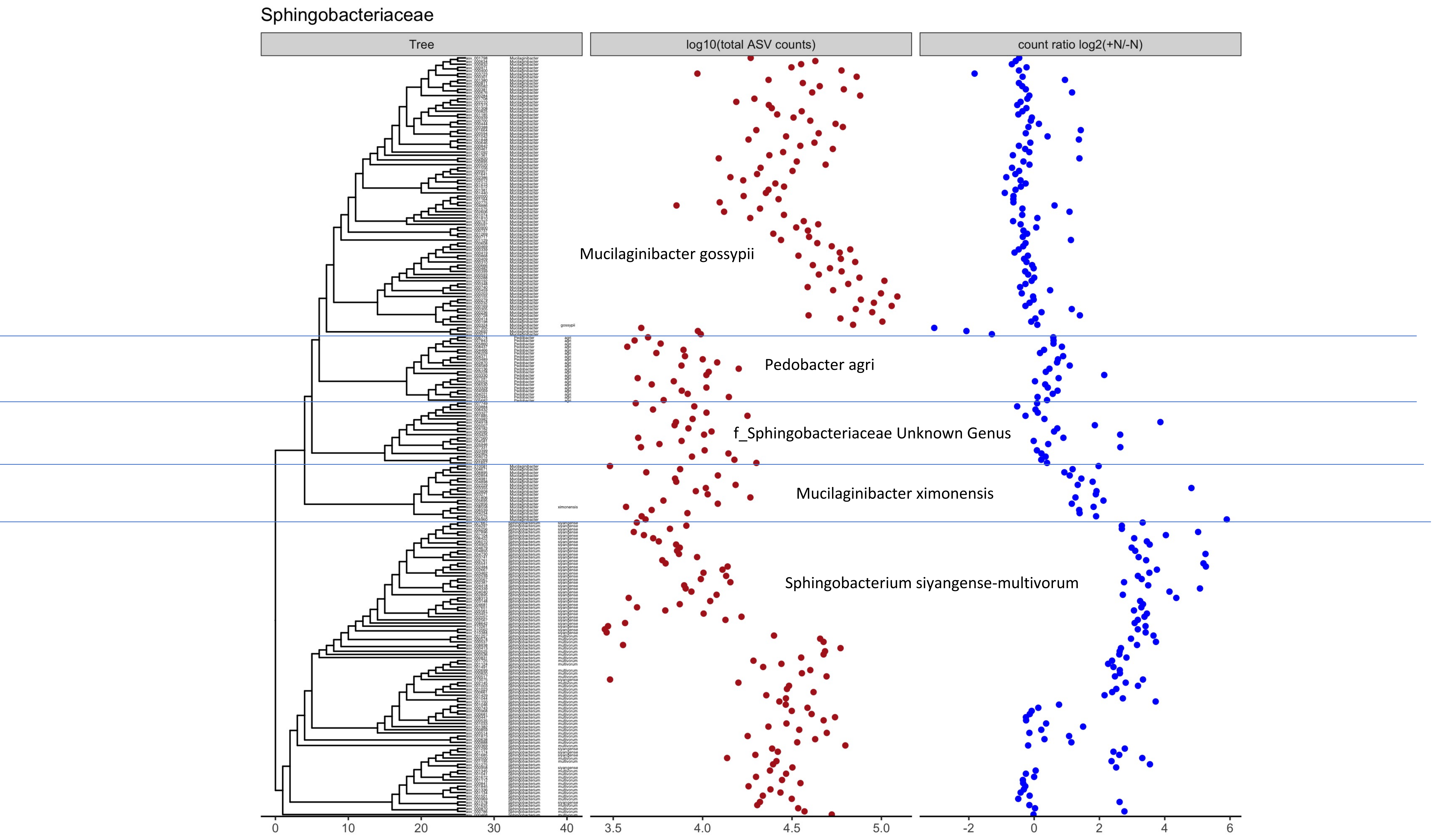

Sphingobacteriaceae
Tree
log10(total ASV counts)
count ratio log2(+N/-N)
Mucilaginibacter gossypii
Pedobacter agri
f_Sphingobacteriaceae Unknown Genus
Mucilaginibacter ximonensis
Sphingobacterium siyangense-multivorum
0
10
20
30
40
3.5
4.0
4.5
5.0
-2
0
2
4
6

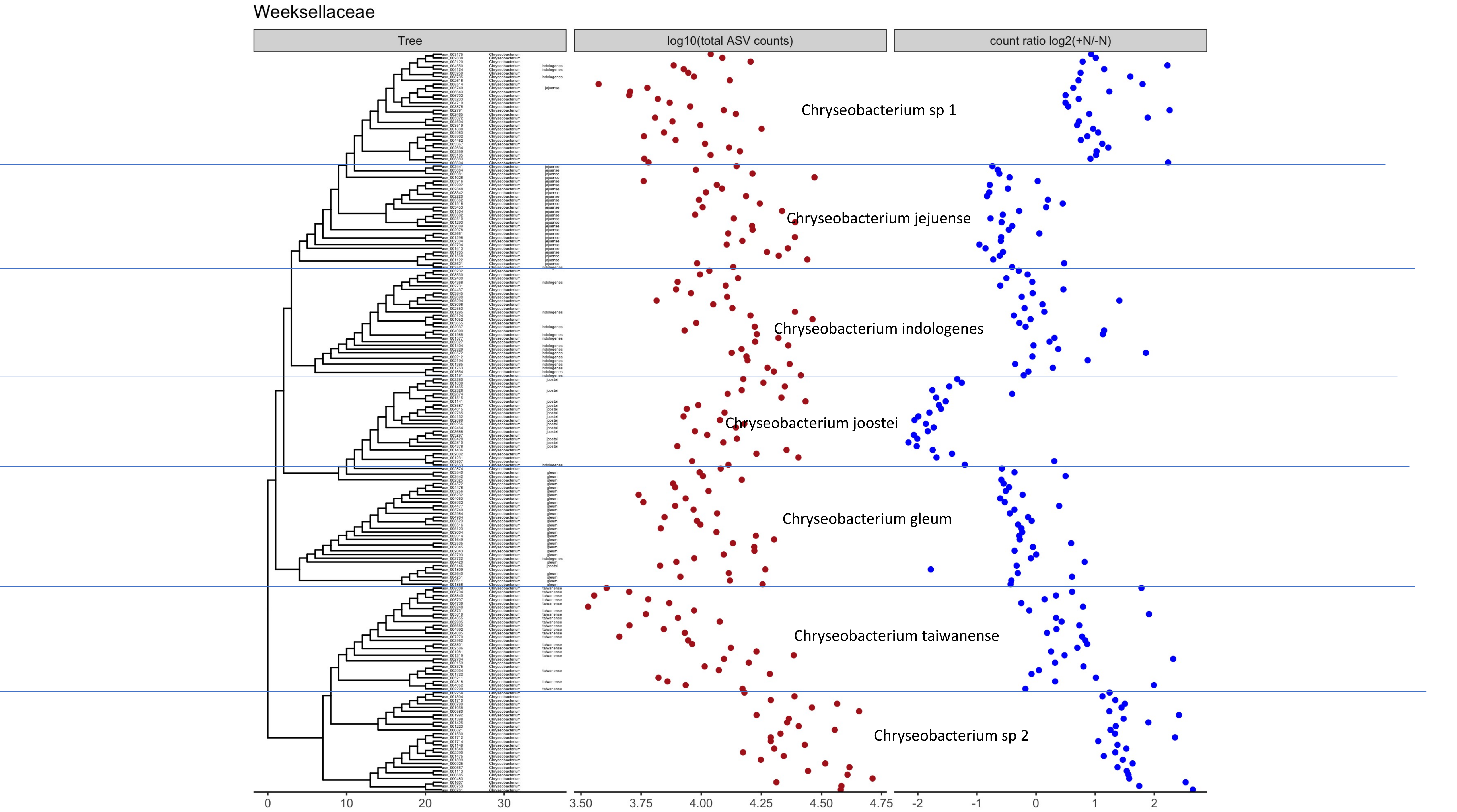

Weeksellaceae
Tree
log10(total ASV counts)
count ratio log2(+N/-N)
Chryseobacterium sp 1
Chryseobacterium jejuense
Chryseobacterium indologenes
Chryseobacterium joostei
Chryseobacterium gleum
Chryseobacterium taiwanense
Chryseobacterium sp 2
0
10
20
30
3.50
3.75
4.00
4.25
4.50
4.75
-2
-1
0
1
2

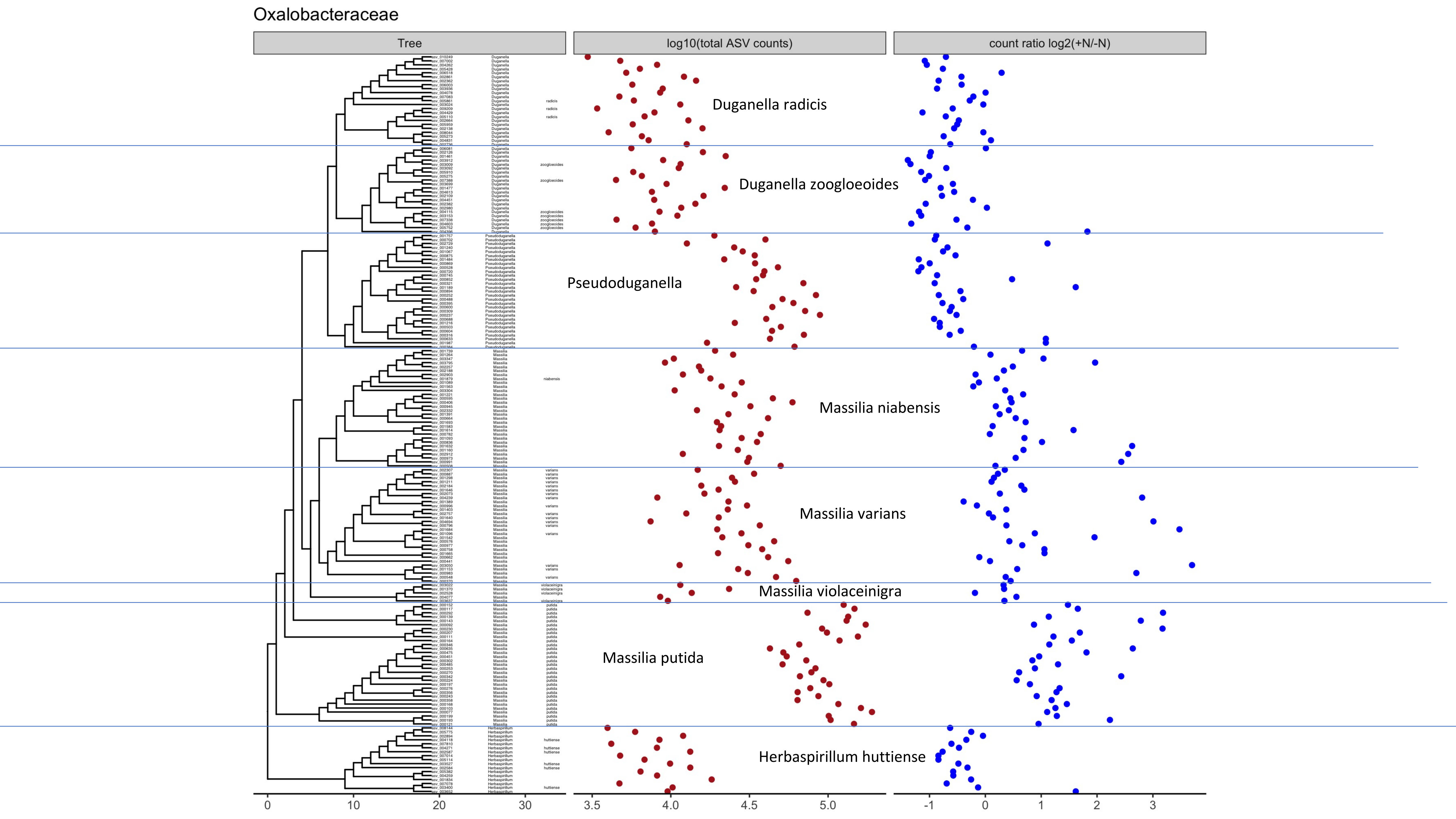

Oxalobacteraceae
Tree
log10(total ASV counts)
count ratio log2(+N/-N)
Duganella radicis
Duganella zoogloeoides
Pseudoduganella
Massilia niabensis
Massilia varians
Massilia violaceinigra
Massilia putida
Herbaspirillum huttiense
0
10
20
30
3.5
4.0
4.5
5.0
-1
0
1
2
3

Burkholderiaceae

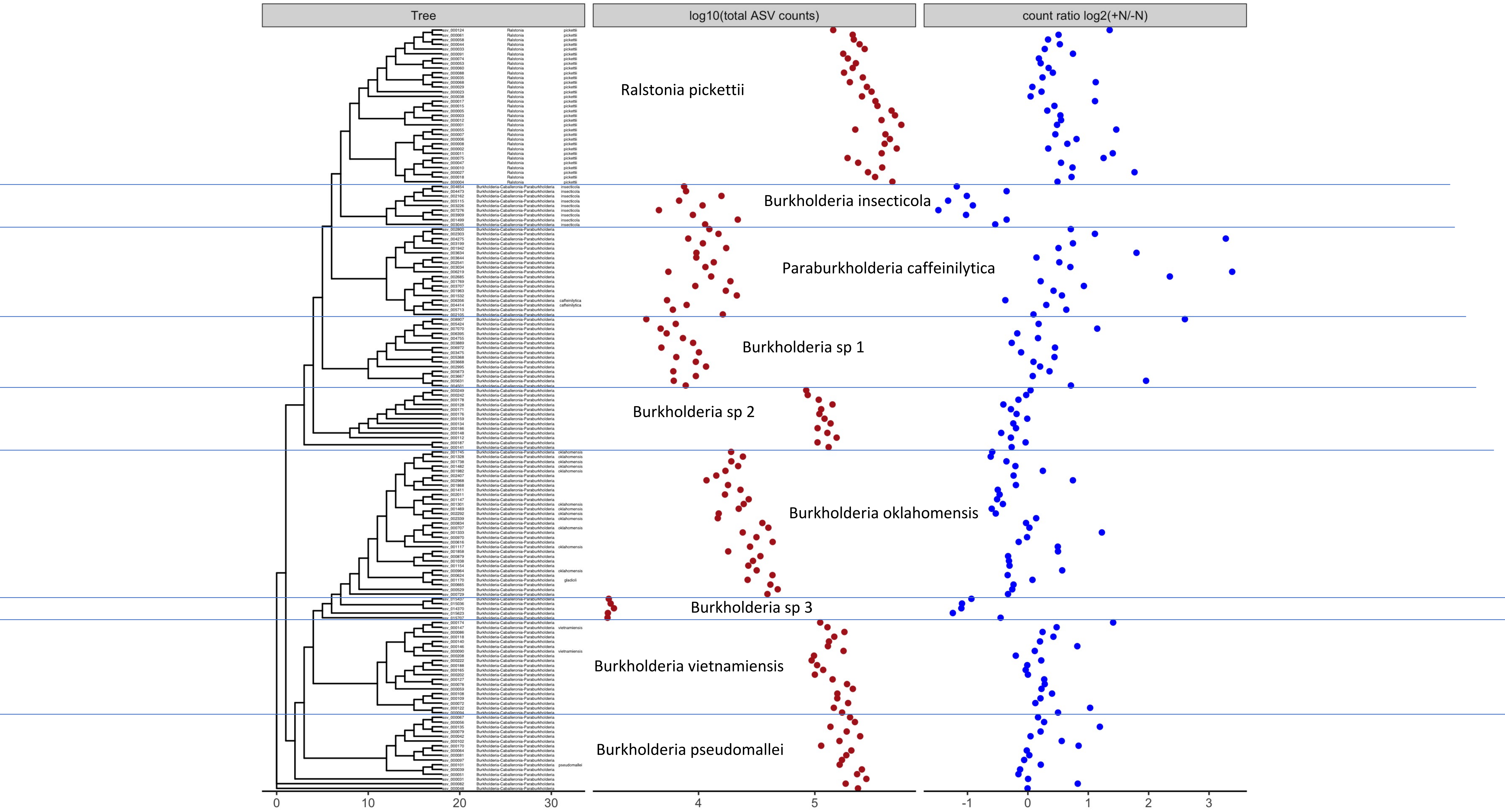

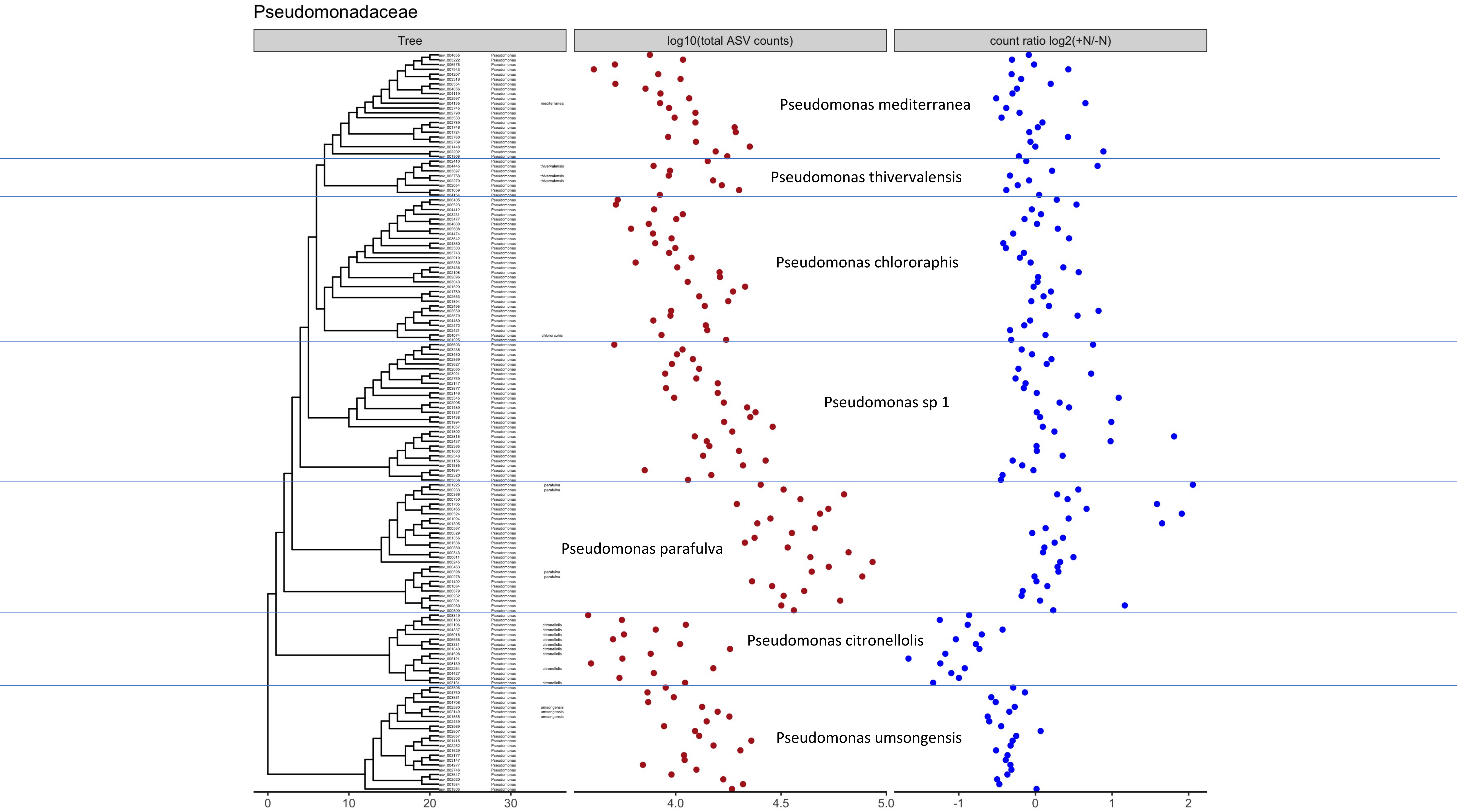
Pseudomonadaceae
Tree
log10(total ASV counts)
count ratio log2(+N/-N)
Pseudomonas mediterranea
Pseudomonas thivervalensis
Pseudomonas chlororaphis
Pseudomonas sp 1
Pseudomonas parafulva
Pseudomonas citronellolis
Pseudomonas umsongensis
0
10
20
30
4.0
4.5
5.0
-1
0
1
2

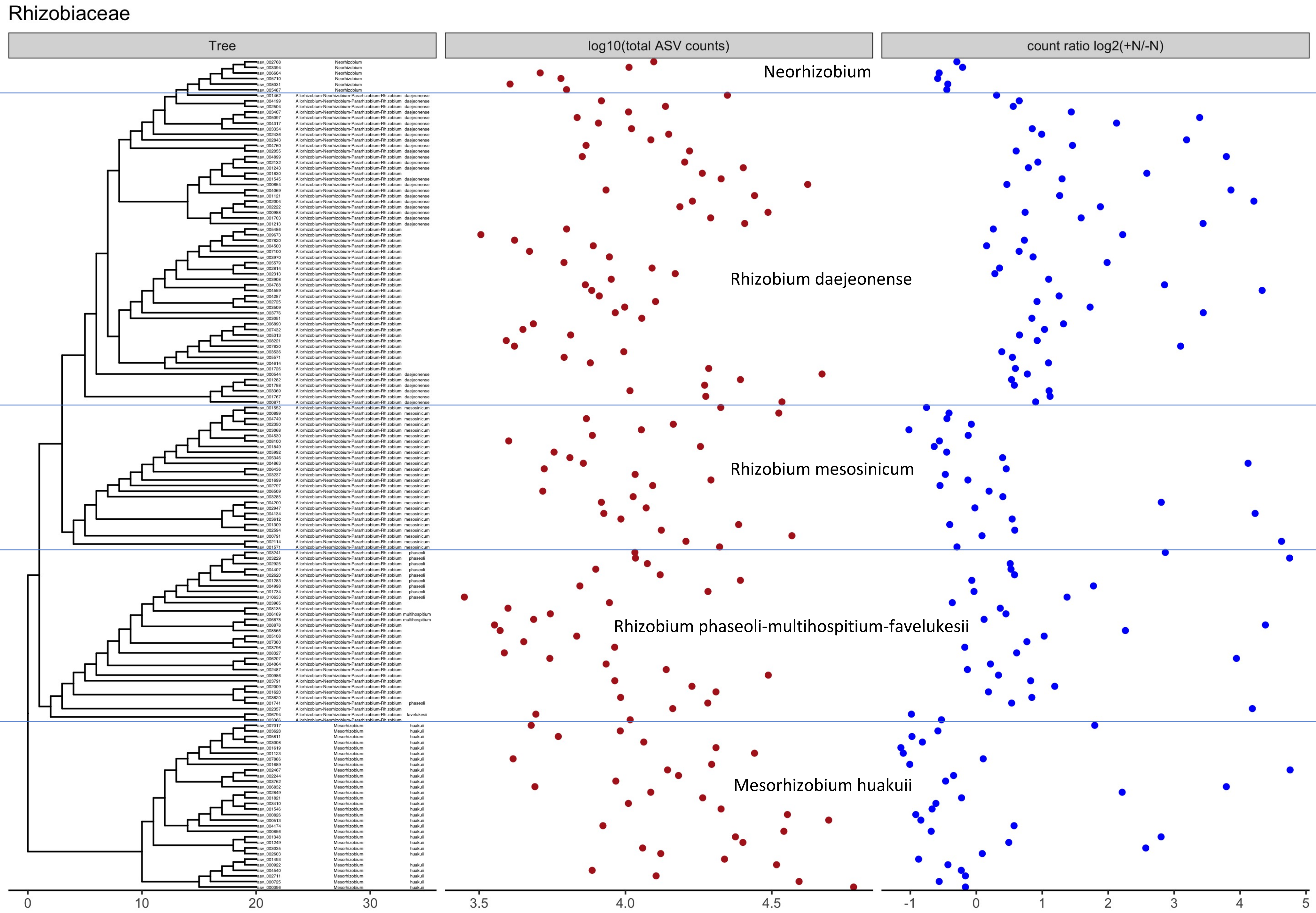

Rhizobiaceae
Tree
log10(total ASV counts)
count ratio log2(+N/-N)
Neorhizobium
Rhizobium daejeonense
Rhizobium mesosinicum
Rhizobium phaseoli-multihospitium-favelukesii
Mesorhizobium huakuii
0
10
20
30
3.5
4.0
4.5
-1
0
1
2
3
4
5

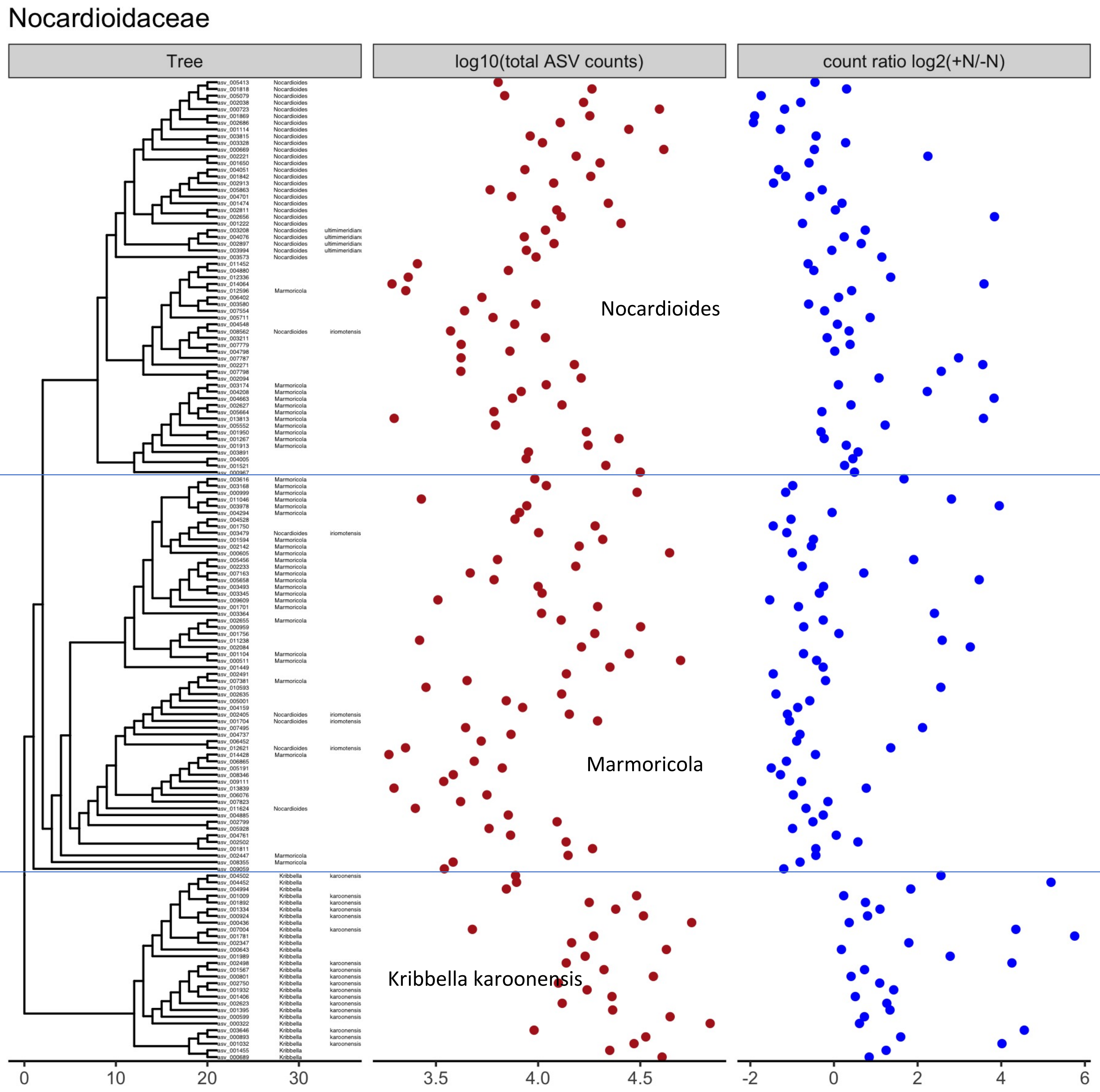
Nocardioidaceae
Tree
log10(total ASV counts)
count ratio log2(+N/-N)
Nocardioides
Marmoricola
Kribbella karoonensis
0
10
20
30
3.5
4.0
4.5
-2
0
2
4
6

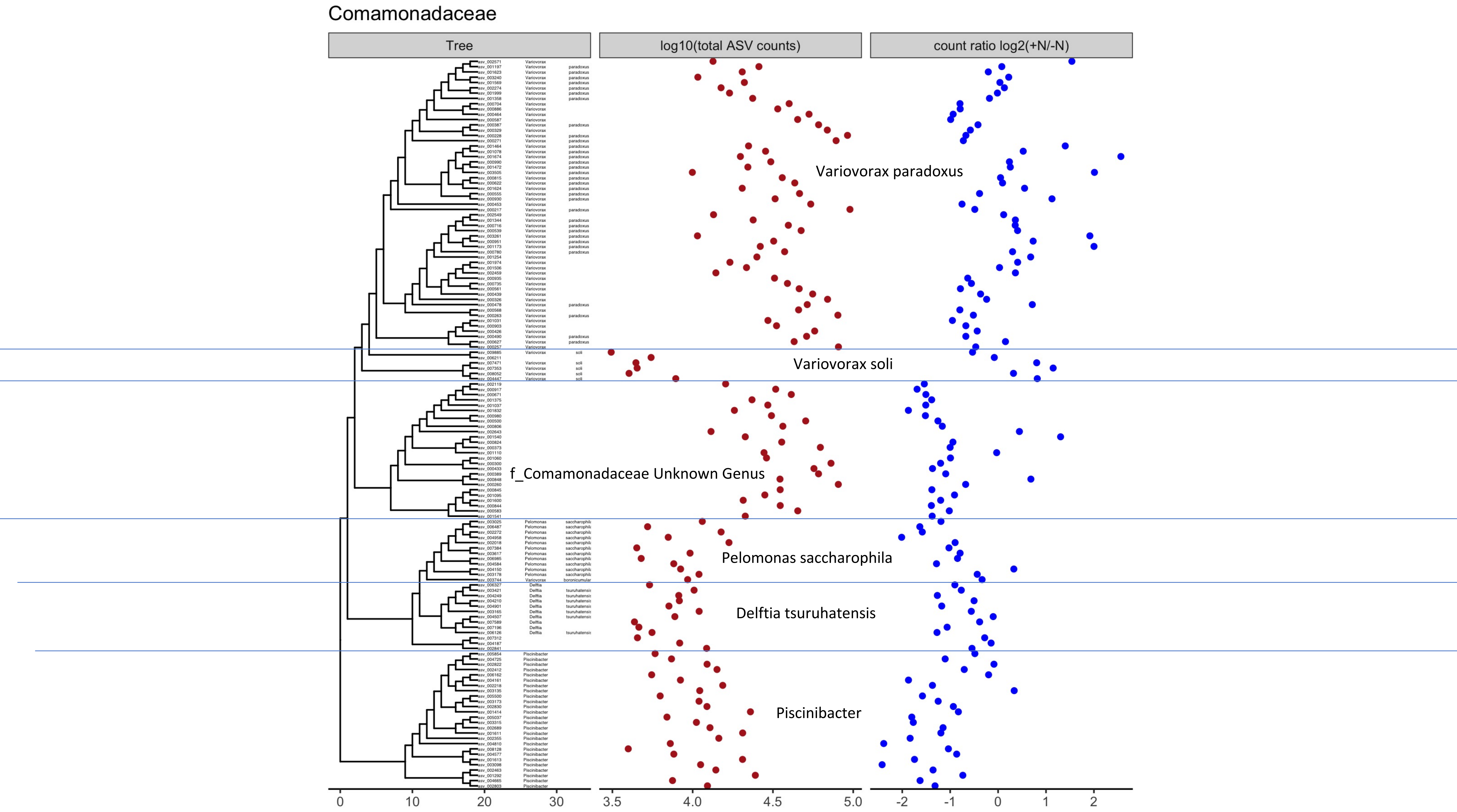
Comamonadaceae
Tree
log10(total ASV counts)
count ratio log2(+N/-N)
Variovorax paradoxus
Variovorax soli
f_Comamonadaceae Unknown Genus
Pelomonas saccharophila
Delftia tsuruhatensis
Piscinibacter
0
10
20
30
3.5
4.0
4.5
5.0
-2
-1
0
1
2

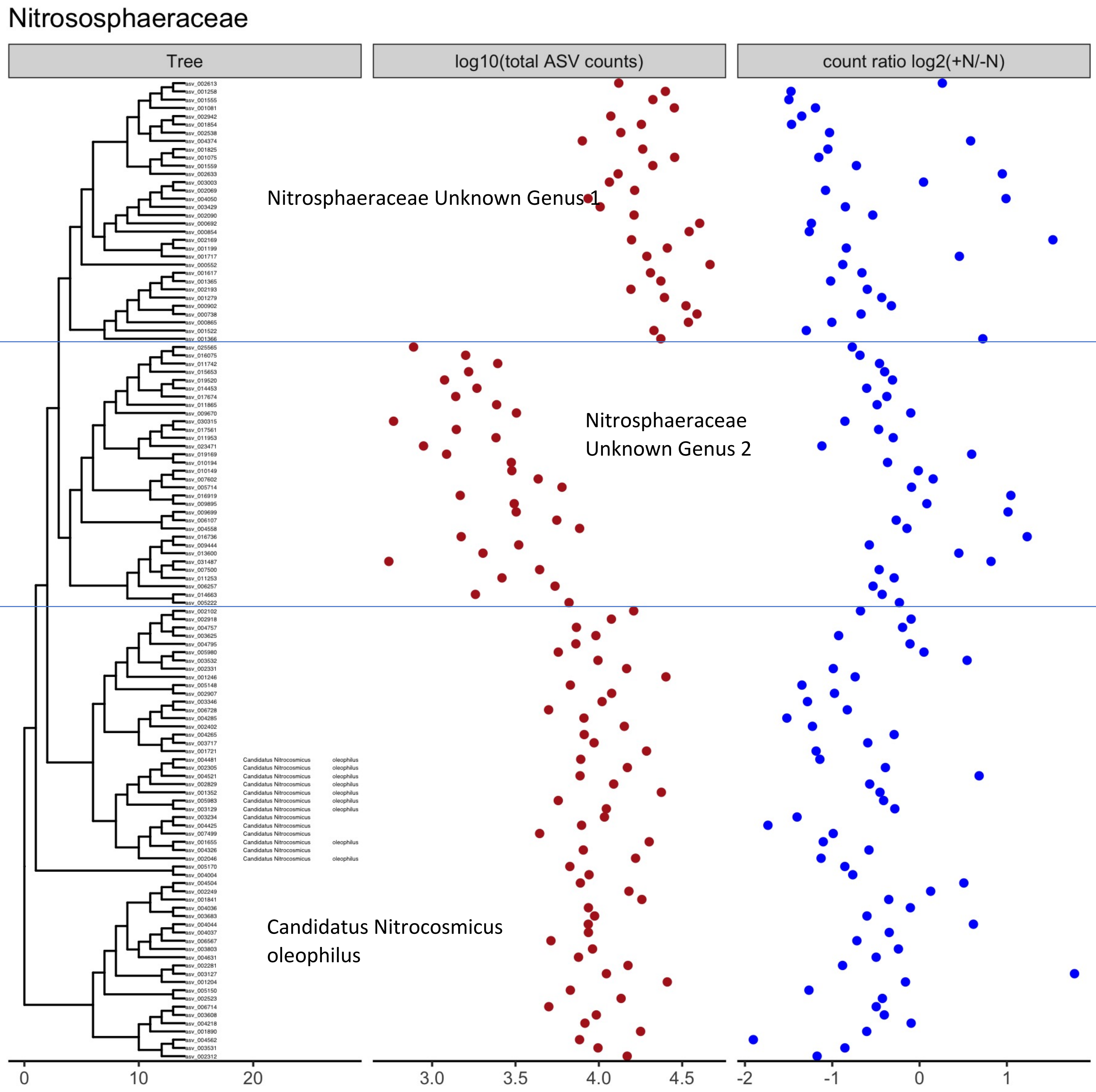
Nitrososphaeraceae
Tree
log10(total ASV counts)
count ratio log2(+N/-N)
Nitrsphaeraceae Unknown Genus 1
Nitrsphaeraceae
Unknown Genus 2
Candidatus Nitrocosmicus
oleophilus
0 10 20
3.0 3.5 4.0 4.5
-2 -1 0 1

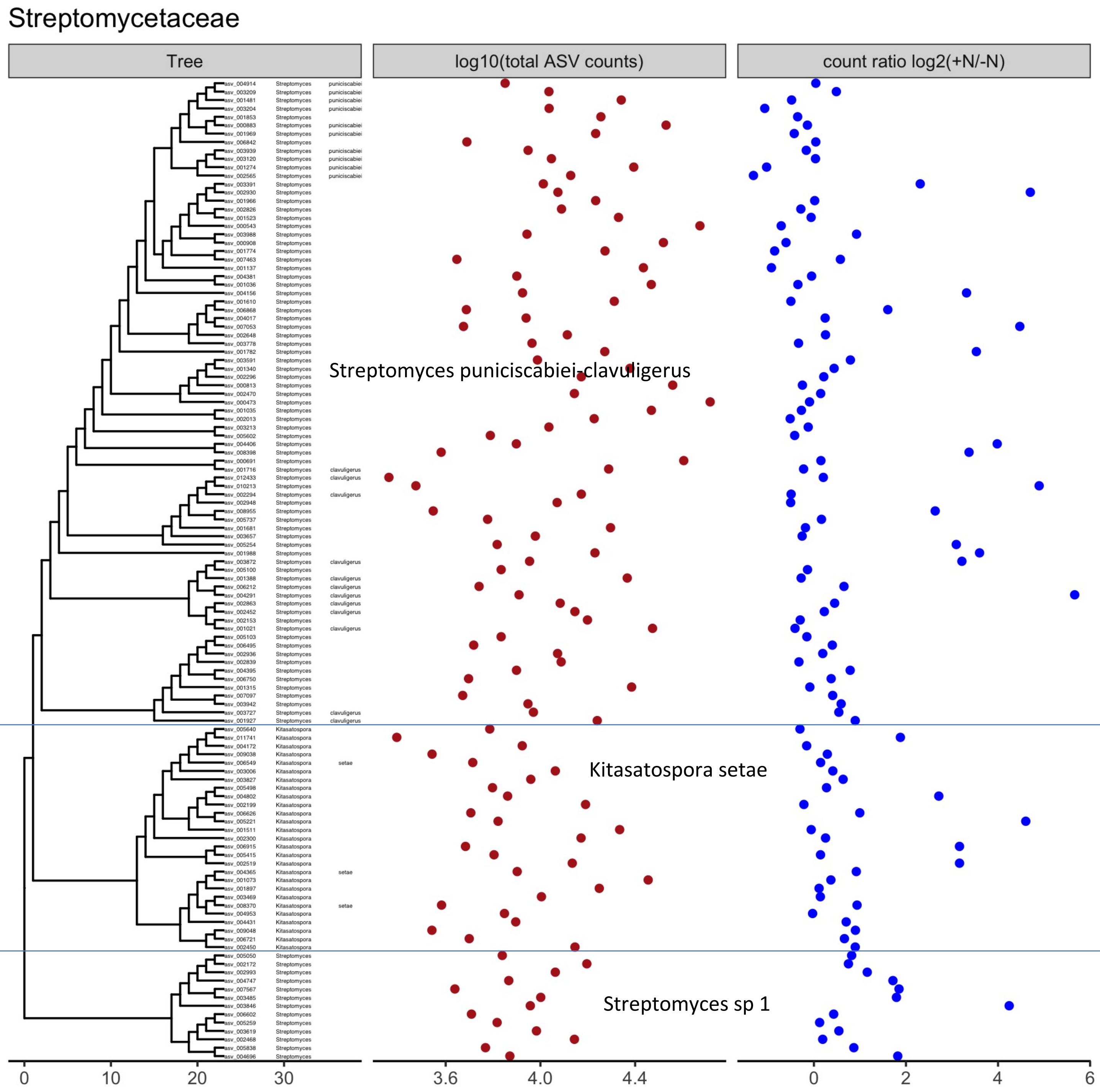
Streptomycetaceae
Tree
log10(total ASV counts)
count ratio log2(+N/-N)
Streptomyces puniciscabiei-clavuligerus
Kitasatospora setae
Streptomyces sp 1
0
10
20
30
3.6
4.0
4.4
0
2
4
6

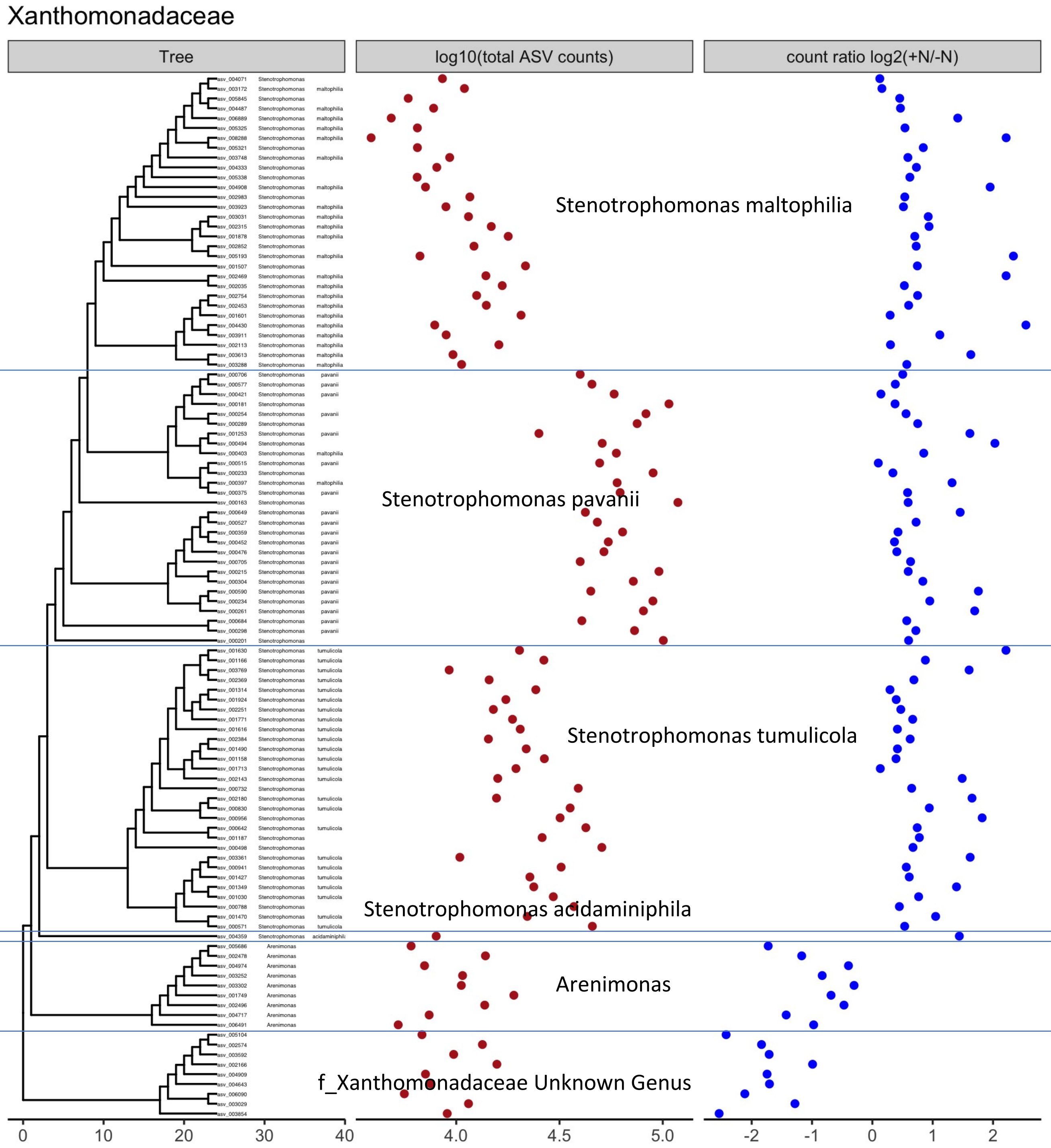
Xanthomonadaceae
Tree
log10(total ASV counts)
count ratio log2(+N/-N)
Stenotrophomonas maltophilia
Stenotrophomonas pavanii
Stenotrophomonas tumulicola
Stenotrophomonas acidaminiphila
Arenimonas
f_Xanthomonadaceae Unknown Genus
0
10
20
30
40
4.0
4.5
5.0
-2
-1
0
1
2

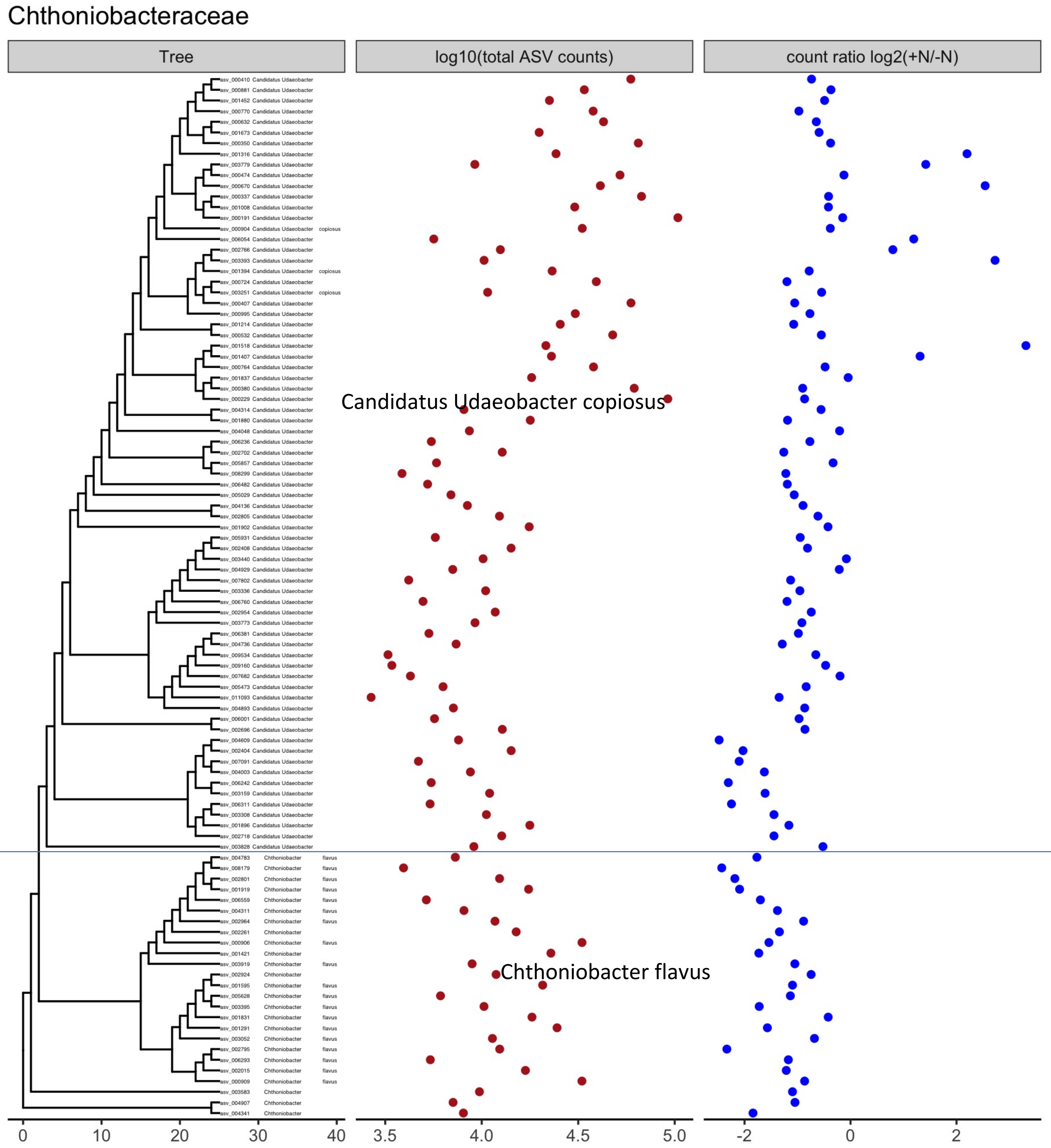

Chthoniobacteraceae
Tree
log10(total ASV counts)
count ratio log2(+N/-N)
Candidatus Udaeobacter copiosus
Chthoniobacter flavus
0
10
20
30
40
3.5
4.0
4.5
5.0
-2
0
2

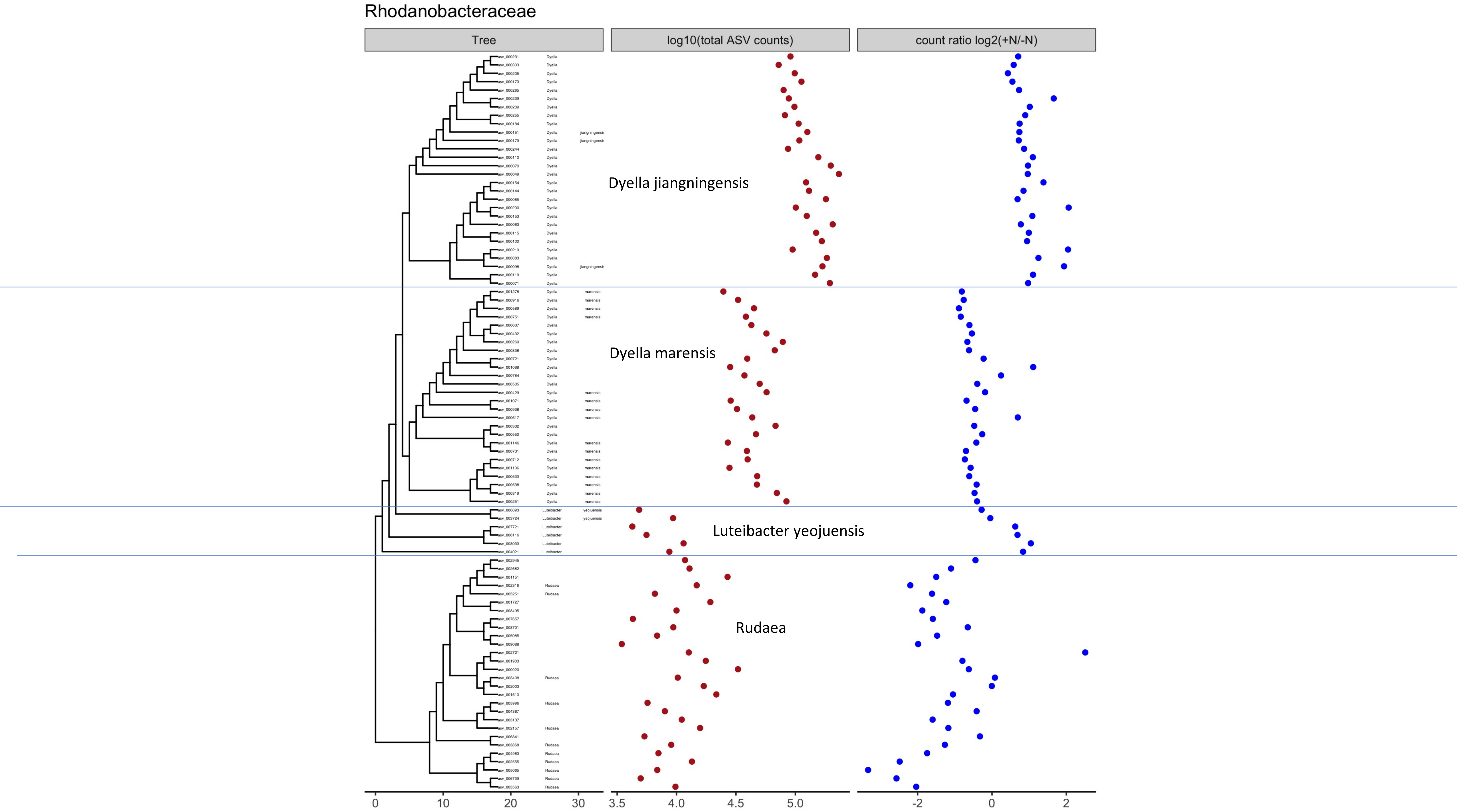
Rhodanobacteraceae
Tree
log10(total ASV counts)
count ratio log2(+N/-N)
Dyella jiangningensis
Dyella marensis
Luteibacter yeojuensis
Rudaea
0
10
20
30
3.5
4.0
4.5
5.0
-2
0
2

Moraxellaceae

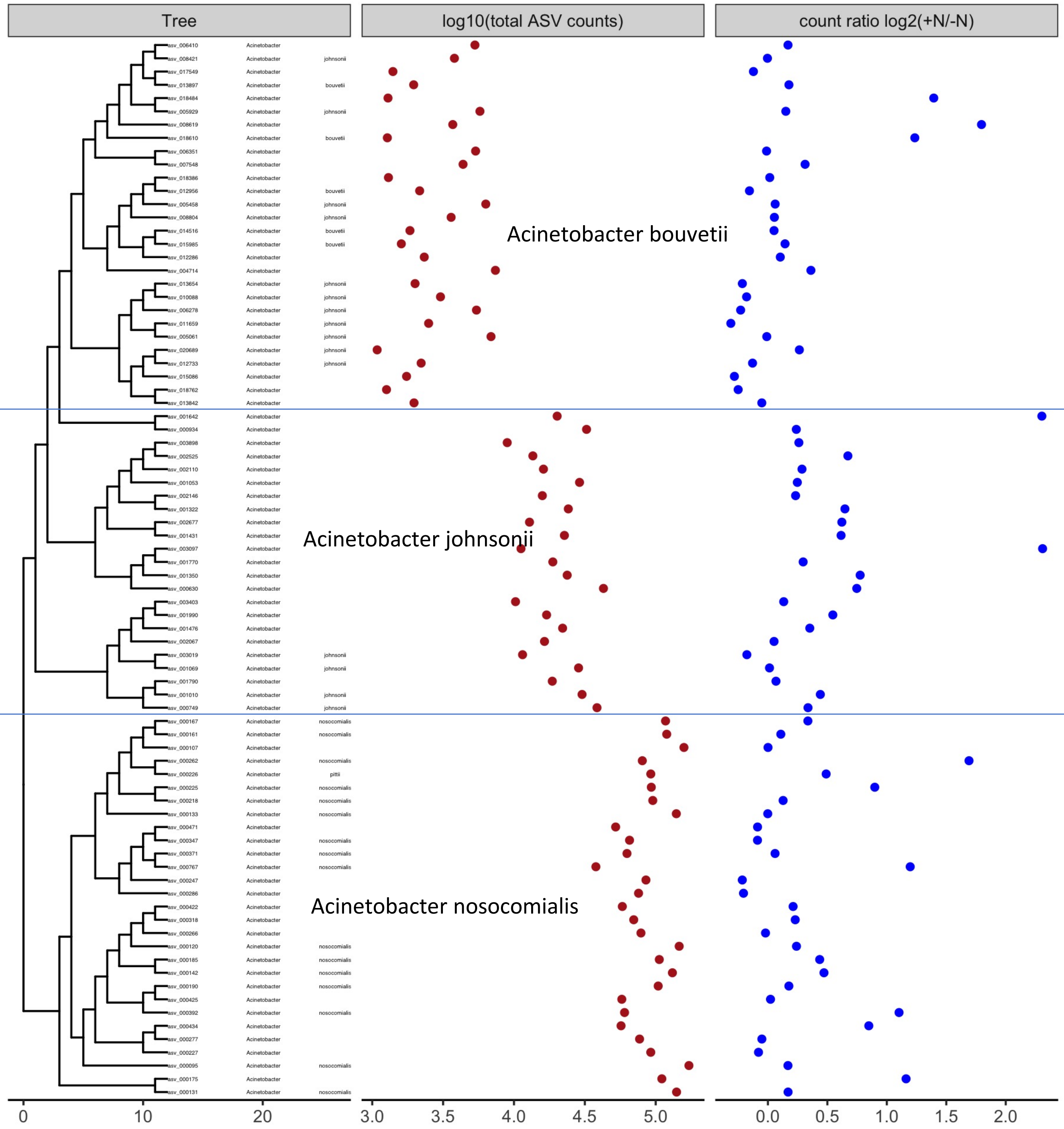

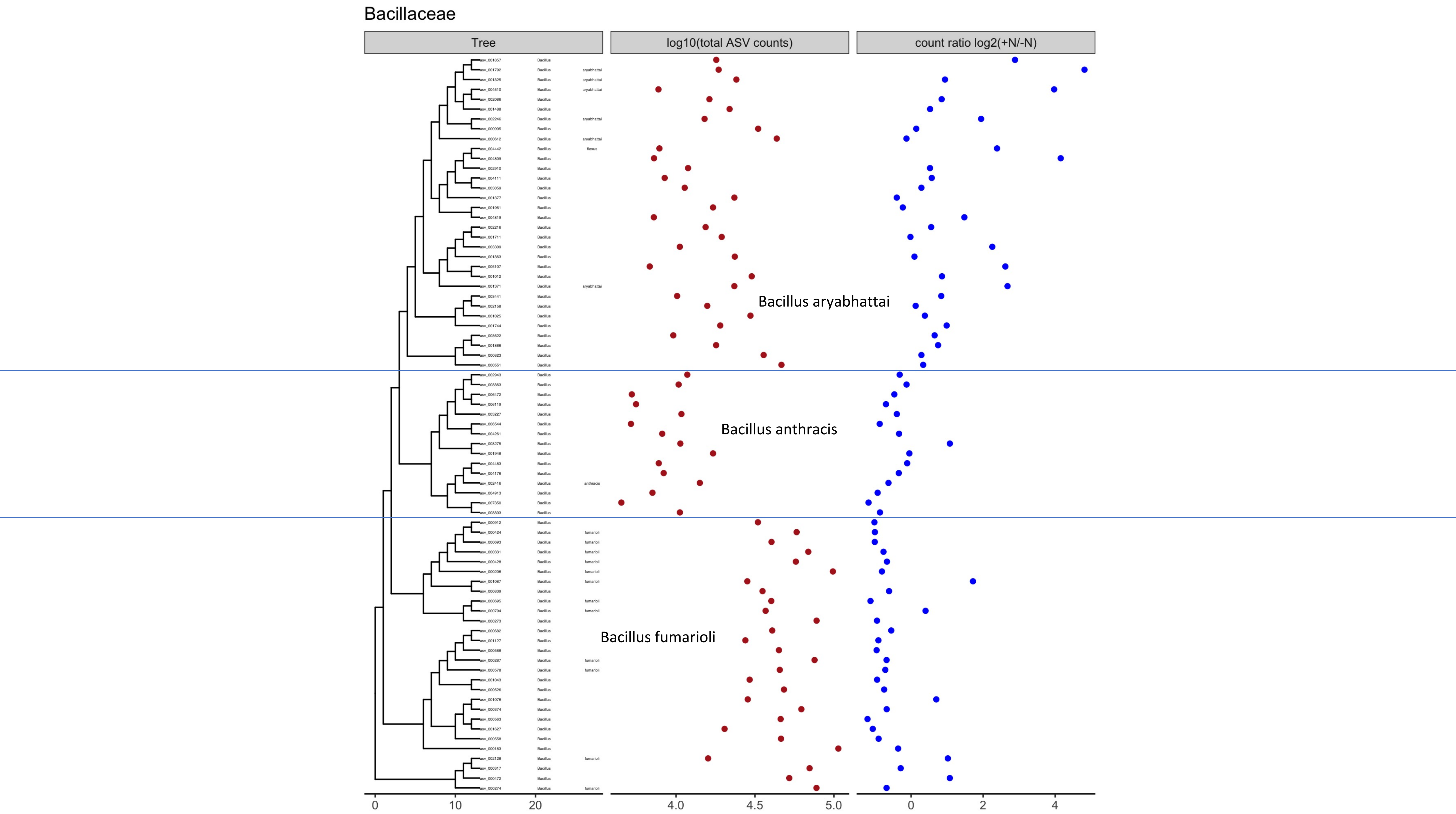
Bacillaceae
Tree
log10(total ASV counts)
count ratio log2(+N/-N)
Bacillus aryabhattai
Bacillus anthracis
Bacillus fumarioli
0
10
20
4.0
4.5
5.0
0
2
4

Spirosomaceae

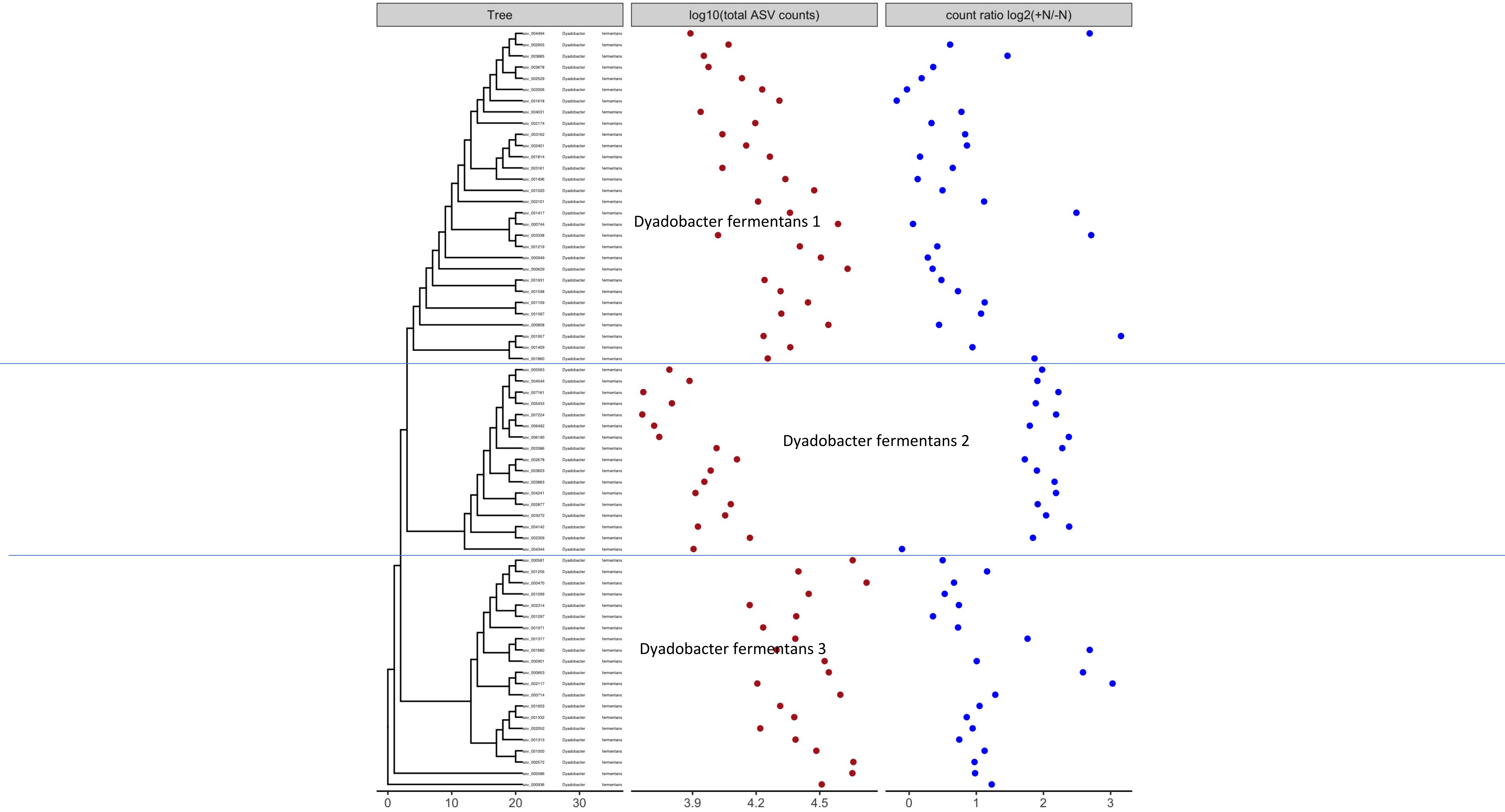

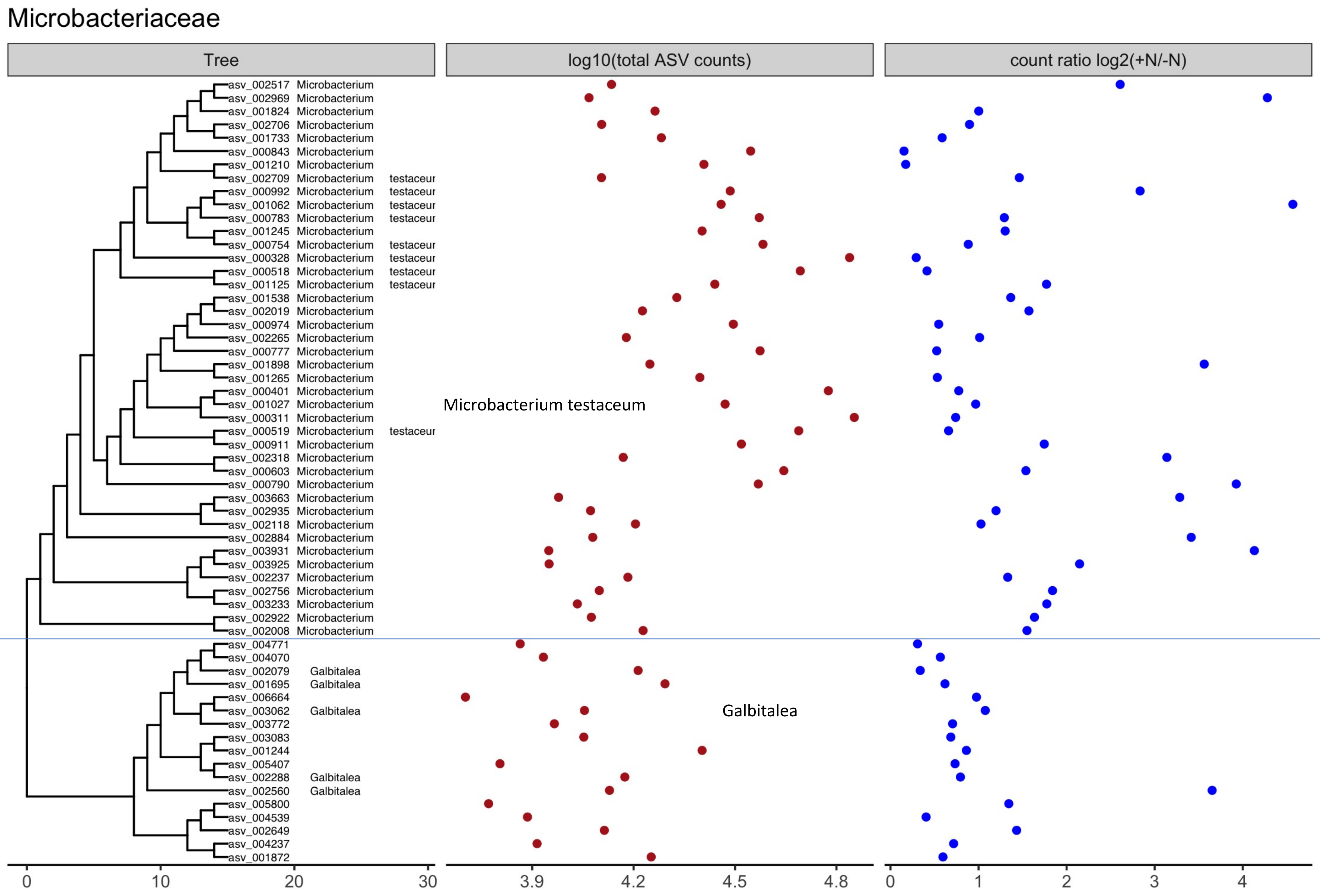

Microbacteriaceae
Tree
log10(total ASV counts)
count ratio log2(+N/-N)
asv_002517 Microbacterium
asv_002969 Microbacterium
asv_001824 Microbacterium
asv_002706 Microbacterium
asv_001733 Microbacterium
asv_000843 Microbacterium
asv_001210 Microbacterium
asv_002709 Microbacterium testaceur
asv_000992 Microbacterium testaceur
asv_001062 Microbacterium testaceur
asv_000783 Microbacterium testaceur
asv_001245 Microbacterium
asv_000754 Microbacterium testaceur
asv_000328 Microbacterium testaceur
asv_000518 Microbacterium testaceur
asv_001125 Microbacterium testaceur
asv_001538 Microbacterium
asv_002019 Microbacterium
asv_000974 Microbacterium
asv_002265 Microbacterium
asv_000777 Microbacterium
asv_001898 Microbacterium
asv_001265 Microbacterium
asv_000401 Microbacterium
asv_001027 Microbacterium
asv_000311 Microbacterium
asv_000519 Microbacterium testaceur
asv_000911 Microbacterium
asv_002318 Microbacterium
asv_000603 Microbacterium
asv_000790 Microbacterium
asv_003663 Microbacterium
asv_002935 Microbacterium
asv_002118 Microbacterium
asv_002884 Microbacterium
asv_003931 Microbacterium
asv_003925 Microbacterium
asv_002237 Microbacterium
asv_002756 Microbacterium
asv_003233 Microbacterium
asv_002922 Microbacterium
asv_002008 Microbacterium
asv_004771
asv_004070
asv_002079 Galbitalea
asv_001695 Galbitalea
asv_006664
asv_003062 Galbitalea
asv_003772
asv_003083
asv_001244
asv_005407
asv_002288 Galbitalea
asv_002560 Galbitalea
asv_005800
asv_004539
asv_002649
asv_004237
asv_001872
Microbacterium testaceum
Galbitalea
0 10 20 30
3.9 4.2 4.5 4.8
0 1 2 3 4

Pyrinomonadaceae

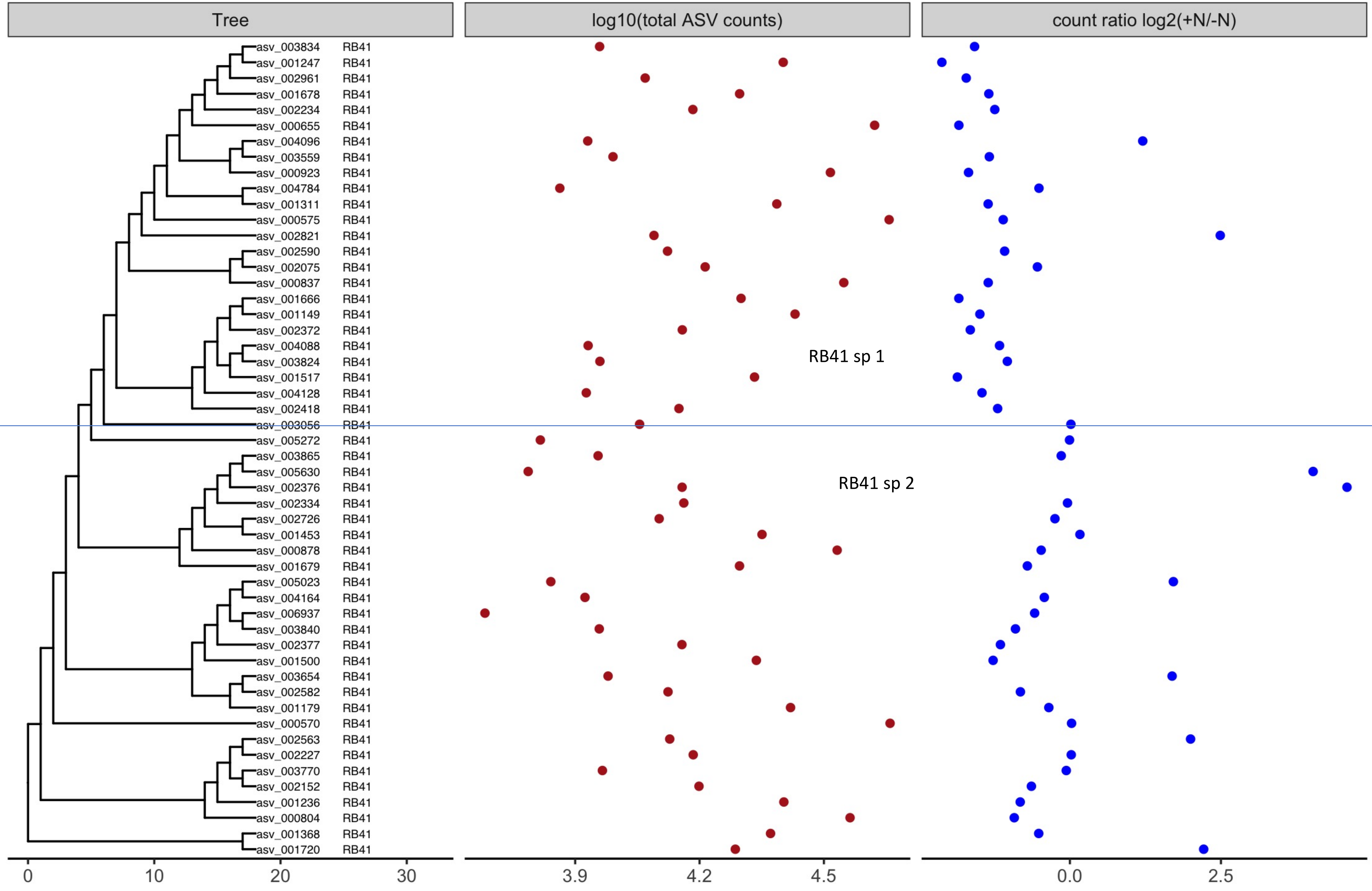

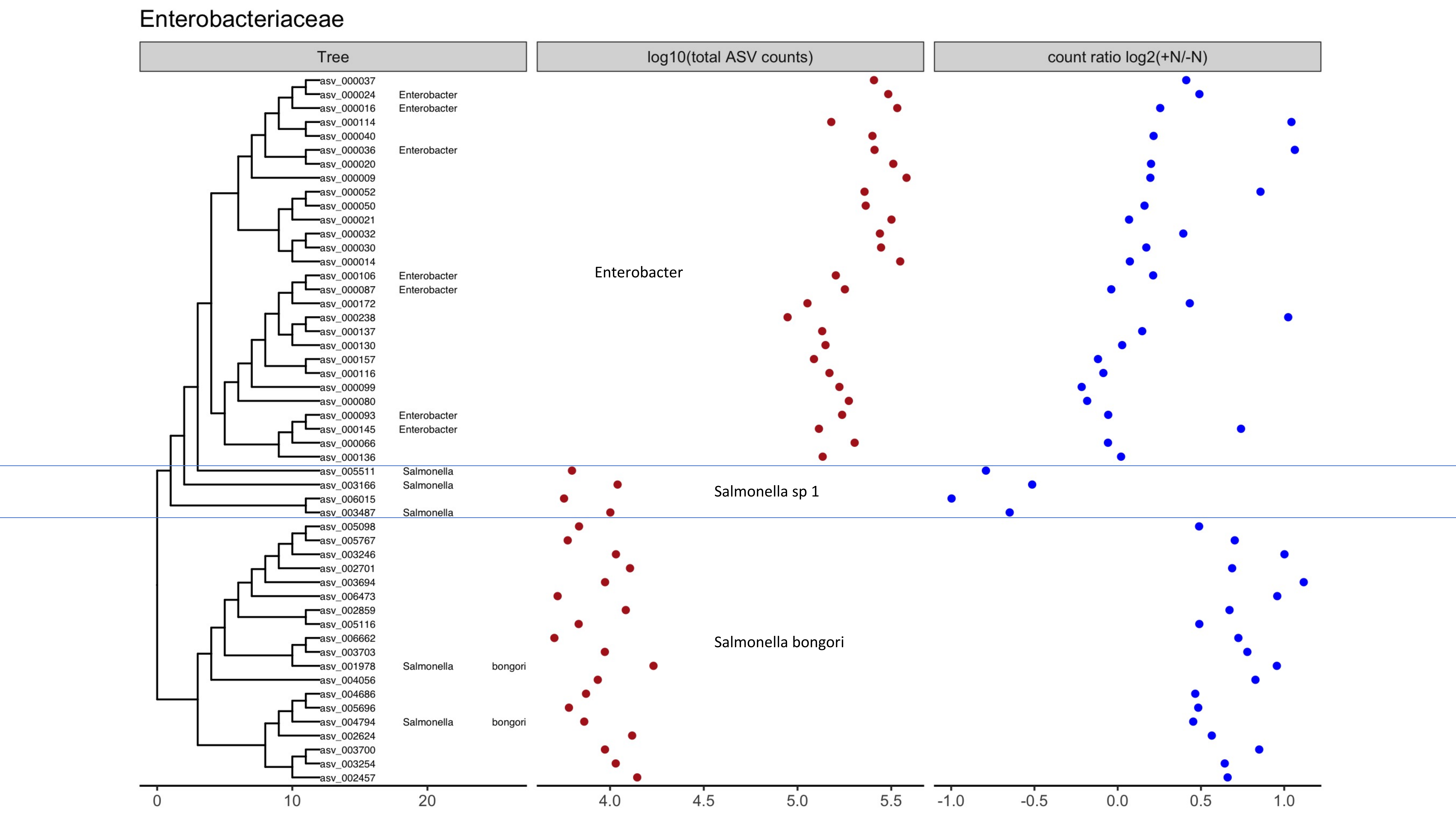

Enterobacteriaceae
Tree
log10(total ASV counts)
count ratio log2(+N/-N)
asv_000037
asv_000024 Enterobacter
asv_000016 Enterobacter
asv_000114
asv_000040
asv_000036 Enterobacter
asv_000020
asv_000009
asv_000052
asv_000050
asv_000021
asv_000032
asv_000030
asv_000014
asv_000106 Enterobacter
asv_000087 Enterobacter
asv_000172
asv_000238
asv_000137
asv_000130
asv_000157
asv_000116
asv_000099
asv_000080
asv_000093 Enterobacter
asv_000145 Enterobacter
asv_000066
asv_000136
asv_005511 Salmonella
asv_003166 Salmonella
asv_006015
asv_003487 Salmonella
asv_005098
asv_005767
asv_003246
asv_002701
asv_003694
asv_006473
asv_002859
asv_005116
asv_006662
asv_003703
asv_001978 Salmonella bongori
asv_004056
asv_004686
asv_005696
asv_004794 Salmonella bongori
asv_002624
asv_003700
asv_003254
asv_002457
Enterobacter
Salmonella sp 1
Salmonella bongori
0
10
20
4.0
4.5
5.0
5.5
-1.0
-0.5
0.0
0.5
1.0

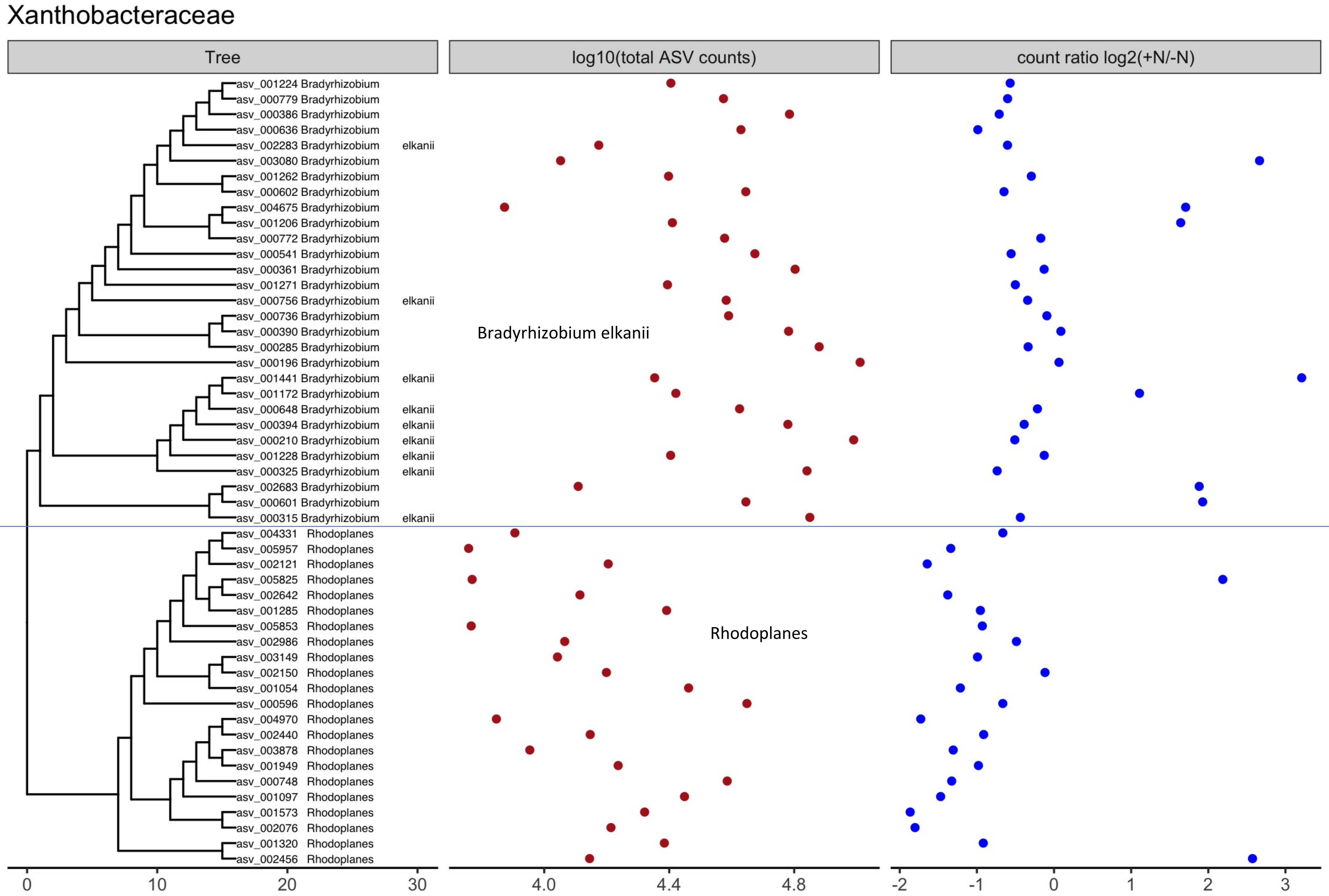
Xanthobacteraceae
Tree
log10(total ASV counts)
count ratio log2(+N/-N)
asv_001224 Bradyrhizobium
asv_000779 Bradyrhizobium
asv_000386 Bradyrhizobium
asv_000636 Bradyrhizobium
asv_002283 Bradyrhizobium elkanii
asv_003080 Bradyrhizobium
asv_001262 Bradyrhizobium
asv_000602 Bradyrhizobium
asv_004675 Bradyrhizobium
asv_001206 Bradyrhizobium
asv_000772 Bradyrhizobium
asv_000541 Bradyrhizobium
asv_000361 Bradyrhizobium
asv_001271 Bradyrhizobium
asv_000756 Bradyrhizobium elkanii
asv_000736 Bradyrhizobium
asv_000390 Bradyrhizobium
asv_000285 Bradyrhizobium
asv_000196 Bradyrhizobium
asv_001441 Bradyrhizobium elkanii
asv_001172 Bradyrhizobium
asv_000648 Bradyrhizobium elkanii
asv_000394 Bradyrhizobium elkanii
asv_000210 Bradyrhizobium elkanii
asv_001228 Bradyrhizobium elkanii
asv_000325 Bradyrhizobium elkanii
asv_002683 Bradyrhizobium
asv_000601 Bradyrhizobium
asv_000315 Bradyrhizobium elkanii
asv_004331 Rhodoplanes
asv_005957 Rhodoplanes
asv_002121 Rhodoplanes
asv_005825 Rhodoplanes
asv_002642 Rhodoplanes
asv_001285 Rhodoplanes
asv_005853 Rhodoplanes
asv_002986 Rhodoplanes
asv_003149 Rhodoplanes
asv_002150 Rhodoplanes
asv_001054 Rhodoplanes
asv_000596 Rhodoplanes
asv_004970 Rhodoplanes
asv_002440 Rhodoplanes
asv_003878 Rhodoplanes
asv_001949 Rhodoplanes
asv_000748 Rhodoplanes
asv_001097 Rhodoplanes
asv_001573 Rhodoplanes
asv_002076 Rhodoplanes
asv_001320 Rhodoplanes
asv_002456 Rhodoplanes
Bradyrhizobium elkanii
Rhodoplanes
0
10
20
30
4.0
4.4
4.8
-2
-1
0
1
2
3

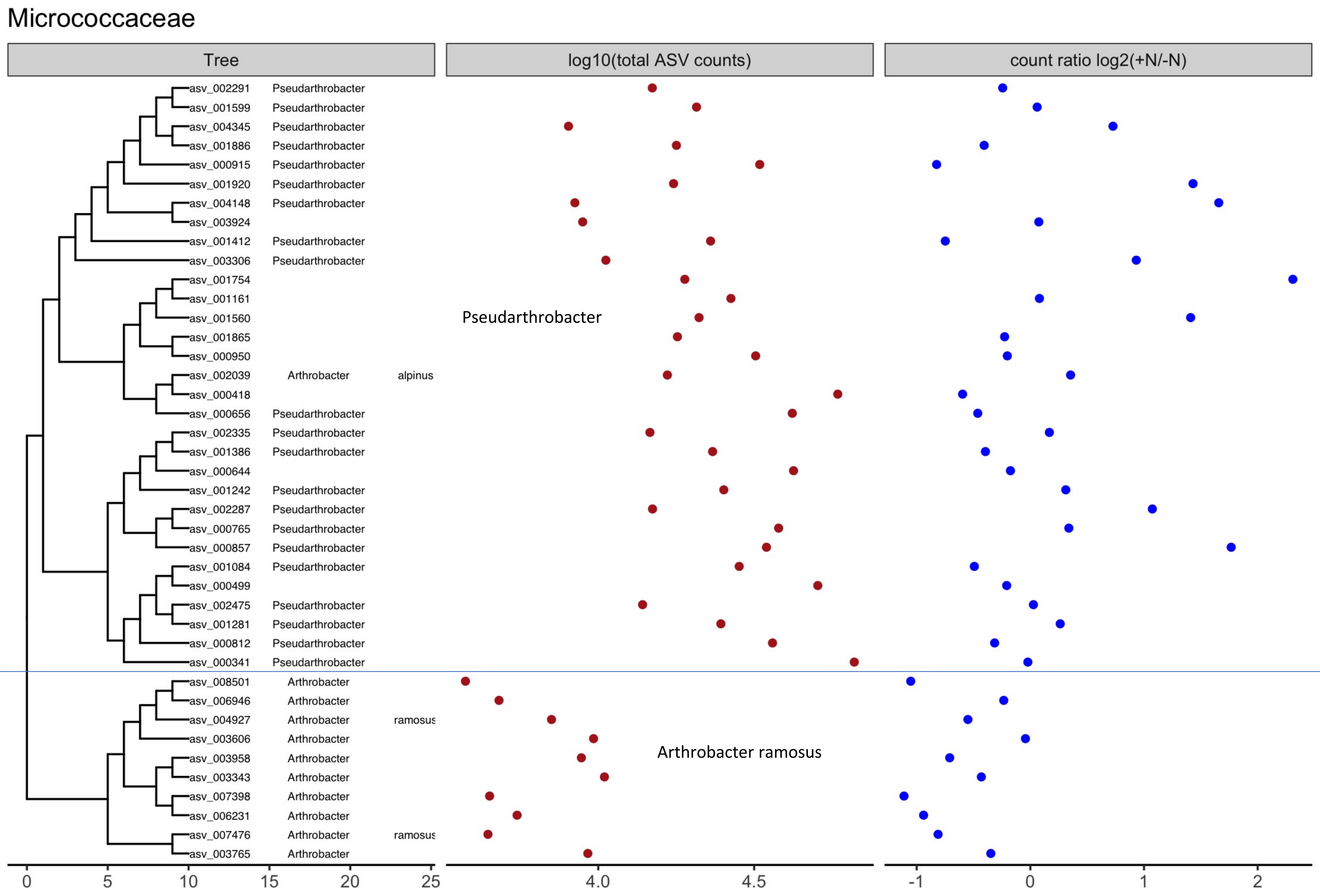

Micrococcaceae
Tree
log10(total ASV counts)
count ratio log2(+N/-N)
asv_002291 Pseudarthrobacter
asv_001599 Pseudarthrobacter
asv_004345 Pseudarthrobacter
asv_001886 Pseudarthrobacter
asv_000915 Pseudarthrobacter
asv_001920 Pseudarthrobacter
asv_004148 Pseudarthrobacter
asv_003924
asv_001412 Pseudarthrobacter
asv_003306 Pseudarthrobacter
asv_001754
asv_001161
asv_001560
asv_001865
asv_000950
asv_002039 Arthrobacter alpinus
asv_000418
asv_000656 Pseudarthrobacter
asv_002335 Pseudarthrobacter
asv_001386 Pseudarthrobacter
asv_000644
asv_001242 Pseudarthrobacter
asv_002287 Pseudarthrobacter
asv_000765 Pseudarthrobacter
asv_000857 Pseudarthrobacter
asv_001084 Pseudarthrobacter
asv_000499
asv_002475 Pseudarthrobacter
asv_001281 Pseudarthrobacter
asv_000812 Pseudarthrobacter
asv_000341 Pseudarthrobacter
asv_008501 Arthrobacter
asv_006946 Arthrobacter
asv_004927 Arthrobacter ramosus
asv_003606 Arthrobacter
asv_003958 Arthrobacter
asv_003343 Arthrobacter
asv_007398 Arthrobacter
asv_006231 Arthrobacter
asv_007476 Arthrobacter ramosus
asv_003765 Arthrobacter
Pseudarthrobacter
Arthrobacter ramosus
0 5 10 15 20 25
4.0 4.5
-1 0 1 2

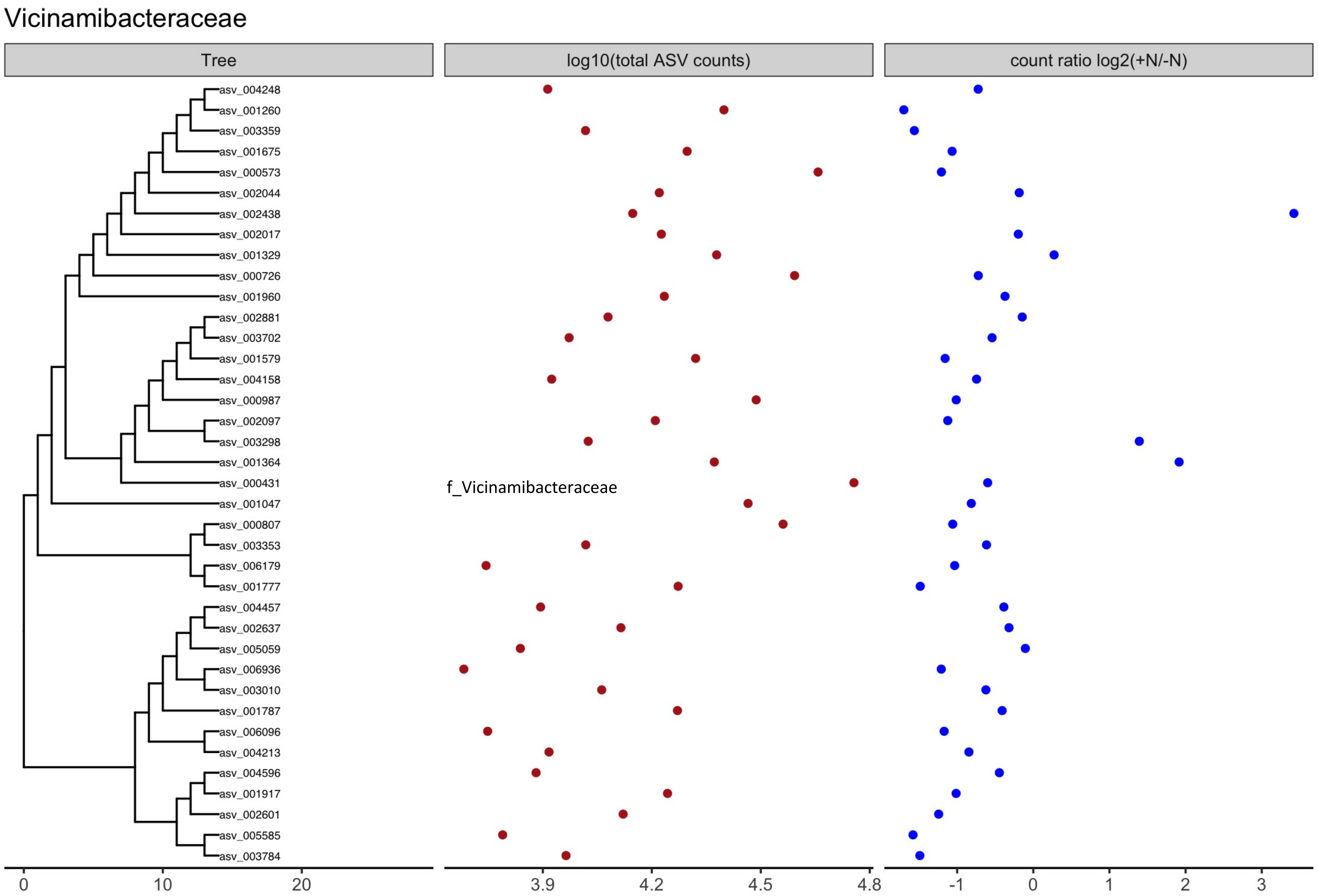

Vicinamibacteraceae
Tree
log10(total ASV counts)
count ratio log2(+N/-N)
asv_004248
asv_001260
asv_003359
asv_001675
asv_000573
asv_002044
asv_002438
asv_002017
asv_001329
asv_000726
asv_001960
asv_002881
asv_003702
asv_001579
asv_004158
asv_000987
asv_002097
asv_003298
asv_001364
asv_000431
asv_001047
asv_000807
asv_003353
asv_006179
asv_001777
asv_004457
asv_002637
asv_005059
asv_006936
asv_003010
asv_001787
asv_006096
asv_004213
asv_004596
asv_001917
asv_002601
asv_005585
asv_003784
f_Vicinamibacteraceae
0
10
20
3.9
4.2
4.5
4.8
-1
0
1
2
3

Caulobacteraceae

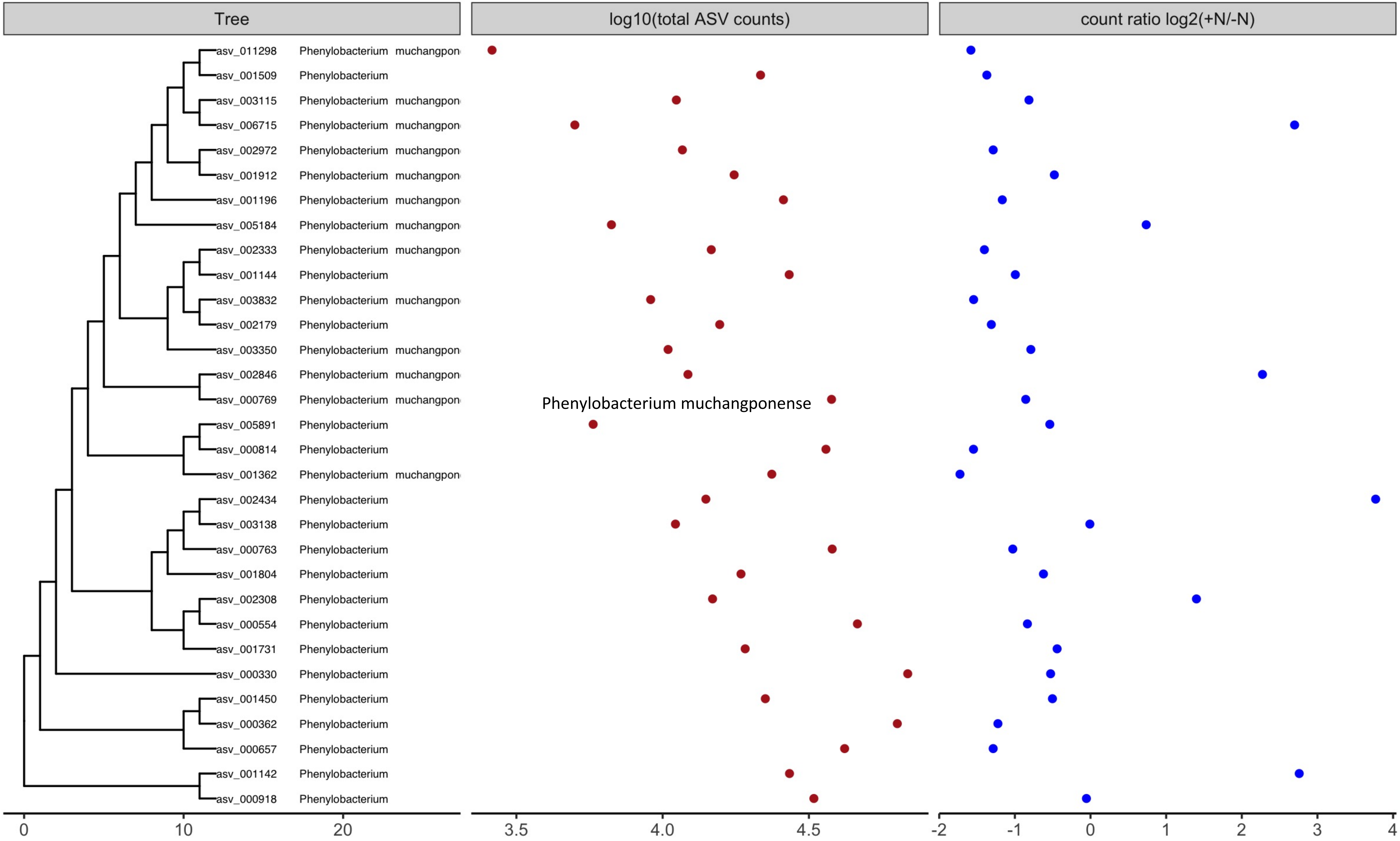

Labraceae

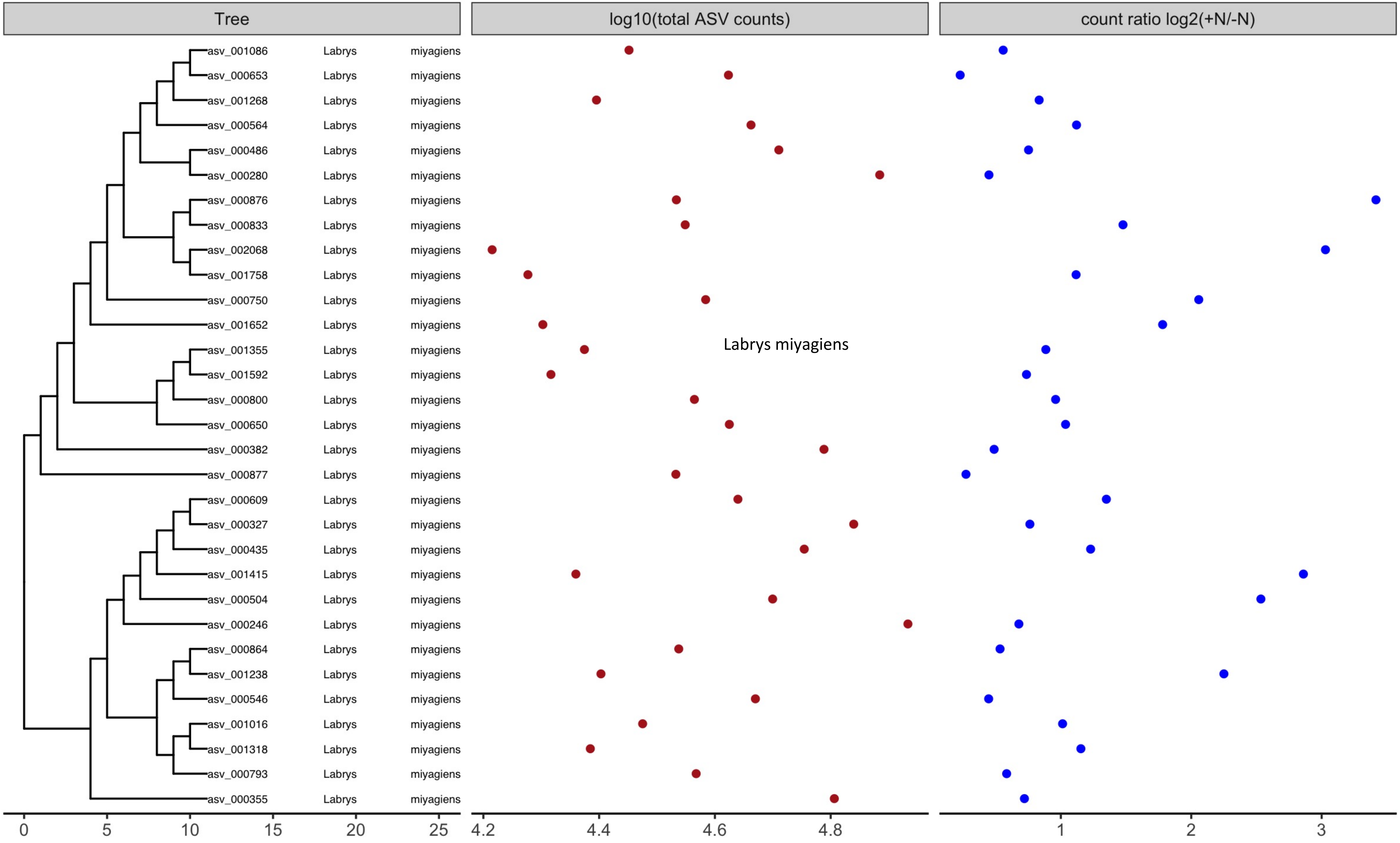
Tree
log10(total ASV counts)
count ratio log2(+N/-N)
asv_001086 Labrys miyagiens
asv_000653 Labrys miyagiens
asv_001268 Labrys miyagiens
asv_000564 Labrys miyagiens
asv_000486 Labrys miyagiens
asv_000280 Labrys miyagiens
asv_000876 Labrys miyagiens
asv_000833 Labrys miyagiens
asv_002068 Labrys miyagiens
asv_001758 Labrys miyagiens
asv_000750 Labrys miyagiens
asv_001652 Labrys miyagiens
asv_001355 Labrys miyagiens
asv_001592 Labrys miyagiens
asv_000800 Labrys miyagiens
asv_000650 Labrys miyagiens
asv_000382 Labrys miyagiens
asv_000877 Labrys miyagiens
asv_000609 Labrys miyagiens
asv_000327 Labrys miyagiens
asv_000435 Labrys miyagiens
asv_001415 Labrys miyagiens
asv_000504 Labrys miyagiens
asv_000246 Labrys miyagiens
asv_000864 Labrys miyagiens
asv_001238 Labrys miyagiens
asv_000546 Labrys miyagiens
asv_001016 Labrys miyagiens
asv_001318 Labrys miyagiens
asv_000793 Labrys miyagiens
asv_000355 Labrys miyagiens
Labrys miyagiens
0 5 10 15 20 25
4.2 4.4 4.6 4.8
1 2 3

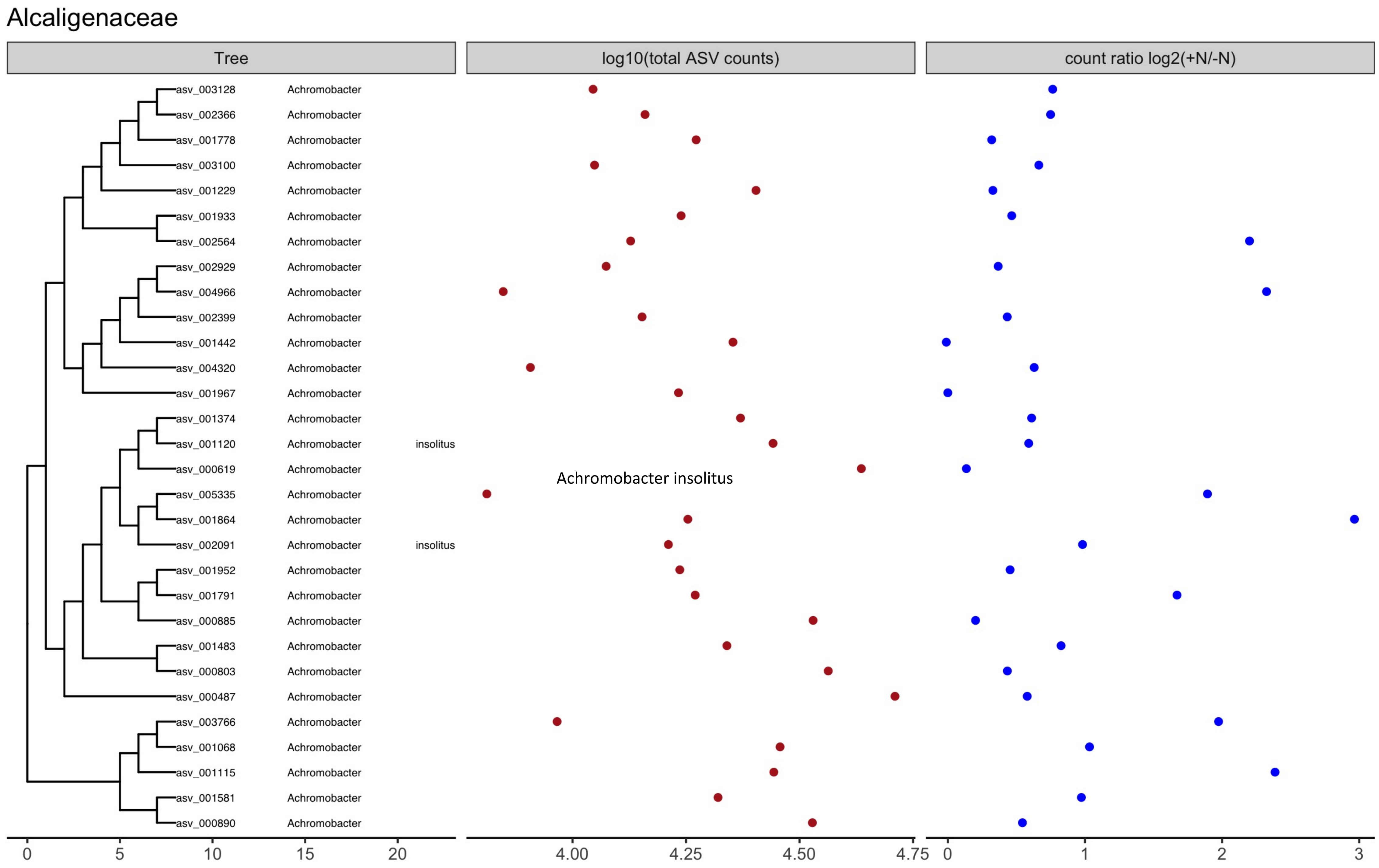
Alcaligenaceae
Tree
log10(total ASV counts)
count ratio log2(+N/-N)
asv_003128 Achromobacter
asv_002366 Achromobacter
asv_001778 Achromobacter
asv_003100 Achromobacter
asv_001229 Achromobacter
asv_001933 Achromobacter
asv_002564 Achromobacter
asv_002929 Achromobacter
asv_004966 Achromobacter
asv_002399 Achromobacter
asv_001442 Achromobacter
asv_004320 Achromobacter
asv_001967 Achromobacter
asv_001374 Achromobacter
asv_001120 Achromobacter insolitus
asv_000619 Achromobacter
asv_005335 Achromobacter
asv_001864 Achromobacter
asv_002091 Achromobacter insolitus
asv_001952 Achromobacter
asv_001791 Achromobacter
asv_000885 Achromobacter
asv_001483 Achromobacter
asv_000803 Achromobacter
asv_000487 Achromobacter
asv_003766 Achromobacter
asv_001068 Achromobacter
asv_001115 Achromobacter
asv_001581 Achromobacter
asv_000890 Achromobacter
Achromobacter insolitus
0 5 10 15 20
4.00 4.25 4.50 4.75
0 1 2 3

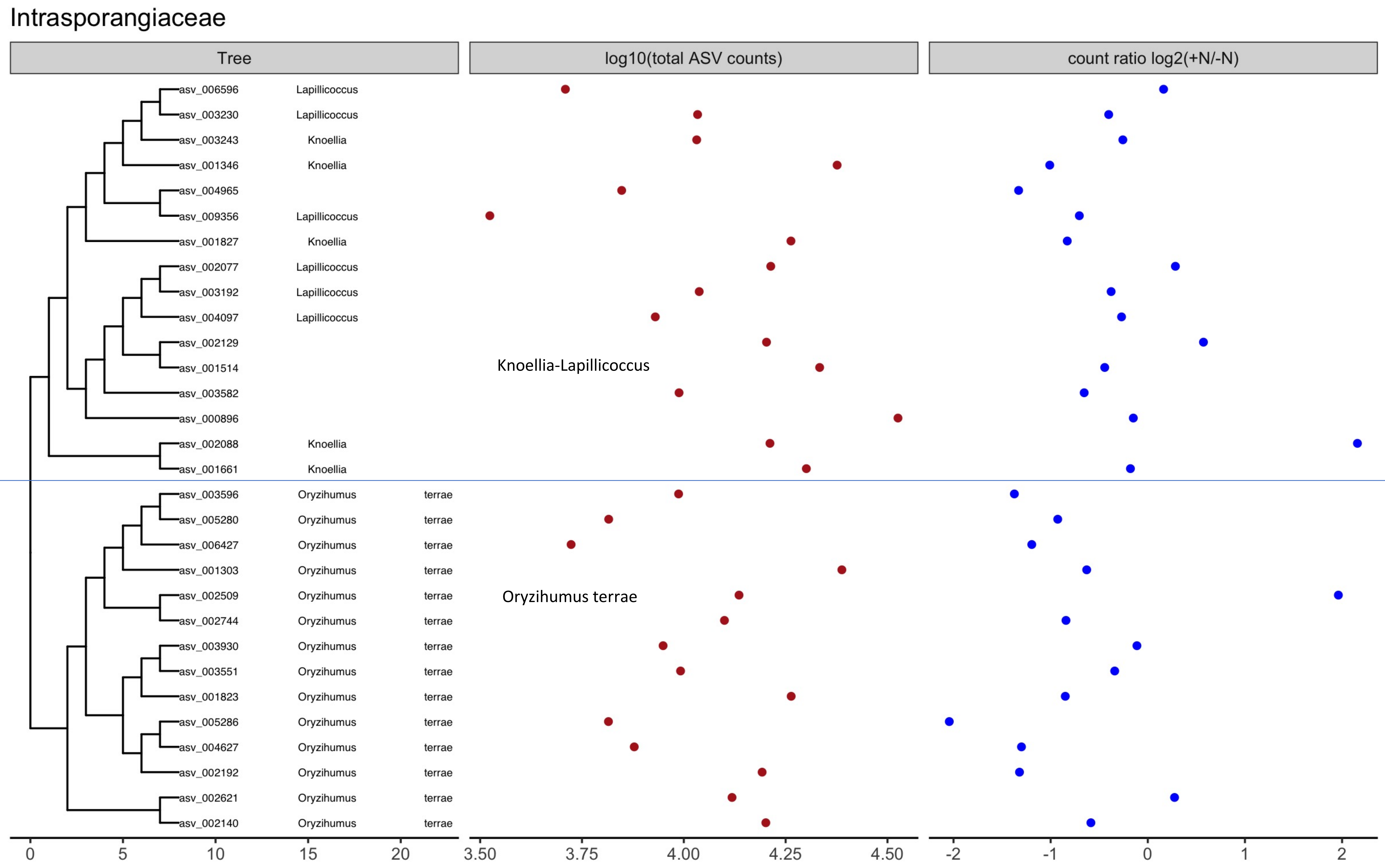

Intrasporangiaceae
Tree
log10(total ASV counts)
count ratio log2(+N/-N)
asv_006596 Lapillicoccus
asv_003230 Lapillicoccus
asv_003243 Knoellia
asv_001346 Knoellia
asv_004965
asv_009356 Lapillicoccus
asv_001827 Knoellia
asv_002077 Lapillicoccus
asv_003192 Lapillicoccus
asv_004097 Lapillicoccus
asv_002129
asv_001514
asv_003582
asv_000896
asv_002088 Knoellia
asv_001661 Knoellia
asv_003596 Oryzihumus terrae
asv_005280 Oryzihumus terrae
asv_006427 Oryzihumus terrae
asv_001303 Oryzihumus terrae
asv_002509 Oryzihumus terrae
asv_002744 Oryzihumus terrae
asv_003930 Oryzihumus terrae
asv_003551 Oryzihumus terrae
asv_001823 Oryzihumus terrae
asv_005286 Oryzihumus terrae
asv_004627 Oryzihumus terrae
asv_002192 Oryzihumus terrae
asv_002621 Oryzihumus terrae
asv_002140 Oryzihumus terrae
Knoellia-Lapillicoccus
Oryzihumus terrae
0 5 10 15 20
3.50 3.75 4.00 4.25 4.50
-2 -1 0 1 2

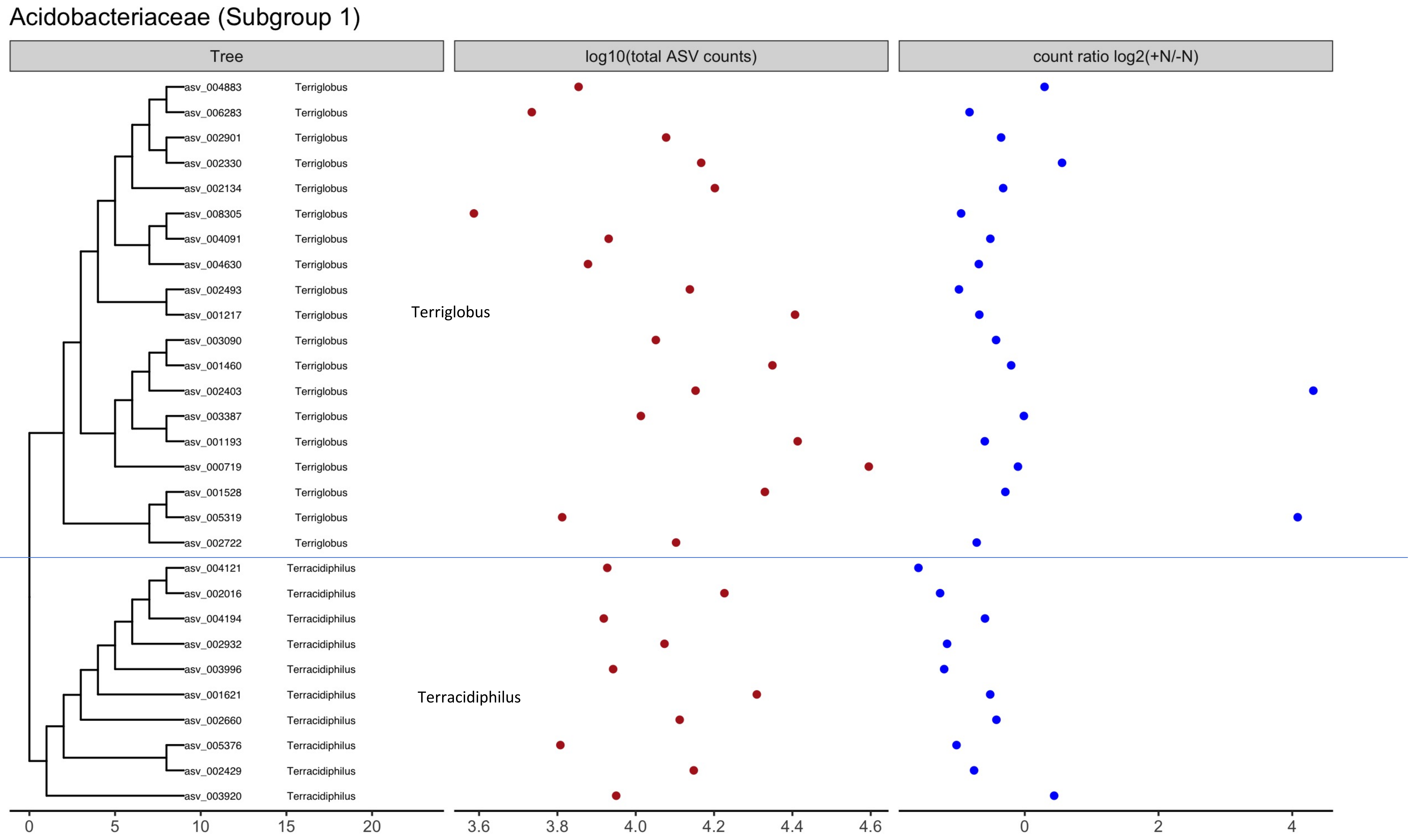
Acidobacteriaceae (Subgroup 1)
Tree
log10(total ASV counts)
count ratio log2(+N/-N)
asv_004883 Terriglobus
asv_006283 Terriglobus
asv_002901 Terriglobus
asv_002330 Terriglobus
asv_002134 Terriglobus
asv_008305 Terriglobus
asv_004091 Terriglobus
asv_004630 Terriglobus
asv_002493 Terriglobus
asv_001217 Terriglobus
asv_003090 Terriglobus
asv_001460 Terriglobus
asv_002403 Terriglobus
asv_003387 Terriglobus
asv_001193 Terriglobus
asv_000719 Terriglobus
asv_001528 Terriglobus
asv_005319 Terriglobus
asv_002722 Terriglobus
asv_004121 Terracidiphilus
asv_002016 Terracidiphilus
asv_004194 Terracidiphilus
asv_002932 Terracidiphilus
asv_003996 Terracidiphilus
asv_001621 Terracidiphilus
asv_002660 Terracidiphilus
asv_005376 Terracidiphilus
asv_002429 Terracidiphilus
asv_003920 Terracidiphilus
Terriglobus
Terracidiphilus
0
5
10
15
20
3.6
3.8
4.0
4.2
4.4
4.6
0
2
4

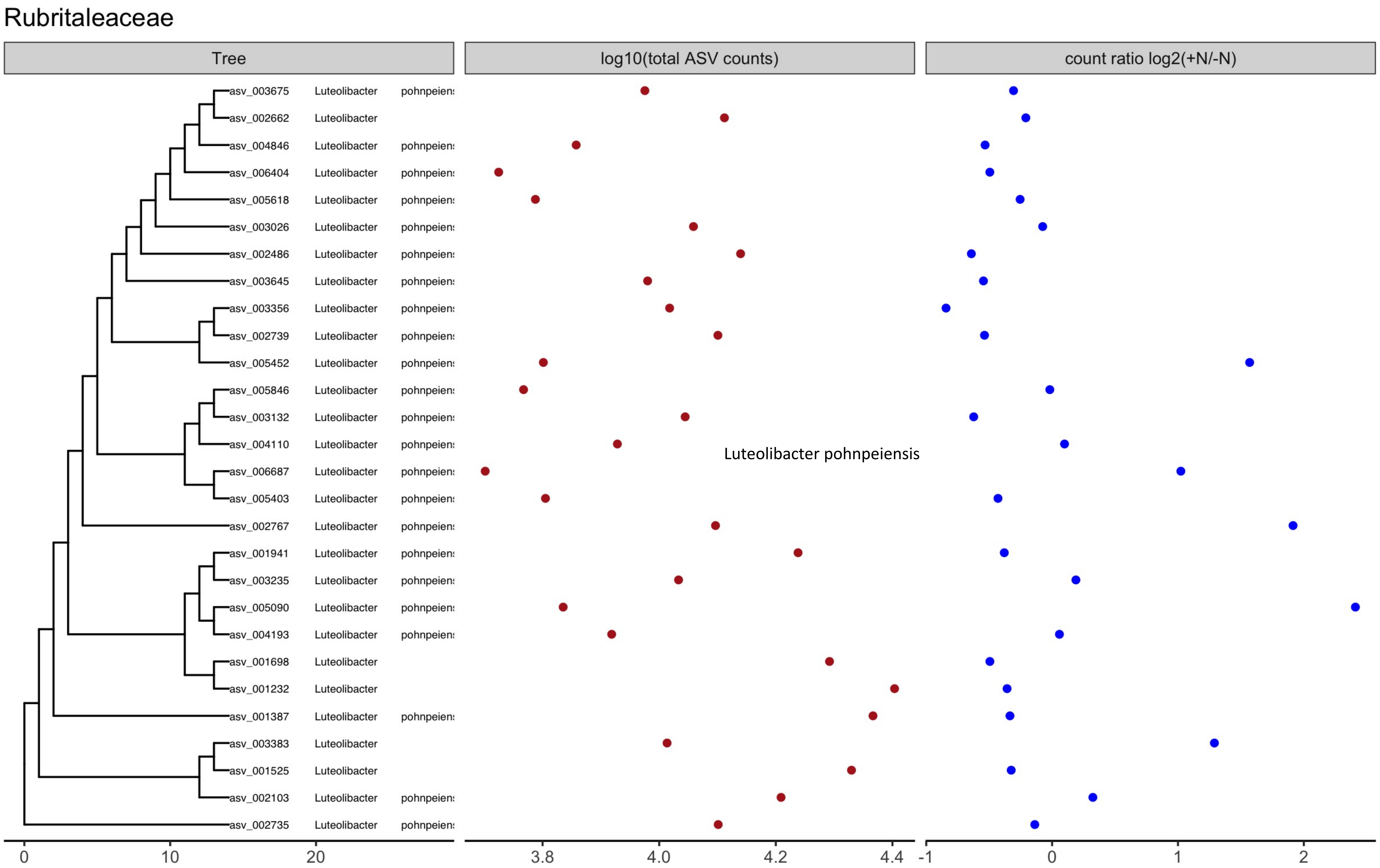

Rubritaleaceae
Tree
log10(total ASV counts)
count ratio log2(+N/-N)
asv_003675 Luteolibacter pohnpeiens
asv_002662 Luteolibacter
asv_004846 Luteolibacter pohnpeiens
asv_006404 Luteolibacter pohnpeiens
asv_005618 Luteolibacter pohnpeiens
asv_003026 Luteolibacter pohnpeiens
asv_002486 Luteolibacter pohnpeiens
asv_003645 Luteolibacter pohnpeiens
asv_003356 Luteolibacter pohnpeiens
asv_002739 Luteolibacter pohnpeiens
asv_005452 Luteolibacter pohnpeiens
asv_005846 Luteolibacter pohnpeiens
asv_003132 Luteolibacter pohnpeiens
asv_004110 Luteolibacter pohnpeiens
asv_006687 Luteolibacter pohnpeiens
asv_005403 Luteolibacter pohnpeiens
asv_002767 Luteolibacter pohnpeiens
asv_001941 Luteolibacter pohnpeiens
asv_003235 Luteolibacter pohnpeiens
asv_005090 Luteolibacter pohnpeiens
asv_004193 Luteolibacter pohnpeiens
asv_001698 Luteolibacter
asv_001232 Luteolibacter
asv_001387 Luteolibacter pohnpeiens
asv_003383 Luteolibacter
asv_001525 Luteolibacter
asv_002103 Luteolibacter pohnpeiens
asv_002735 Luteolibacter pohnpeiens
Luteolibacter pohnpeiensis
0
10
20
3.8
4.0
4.2
4.4
-1
0
1
2

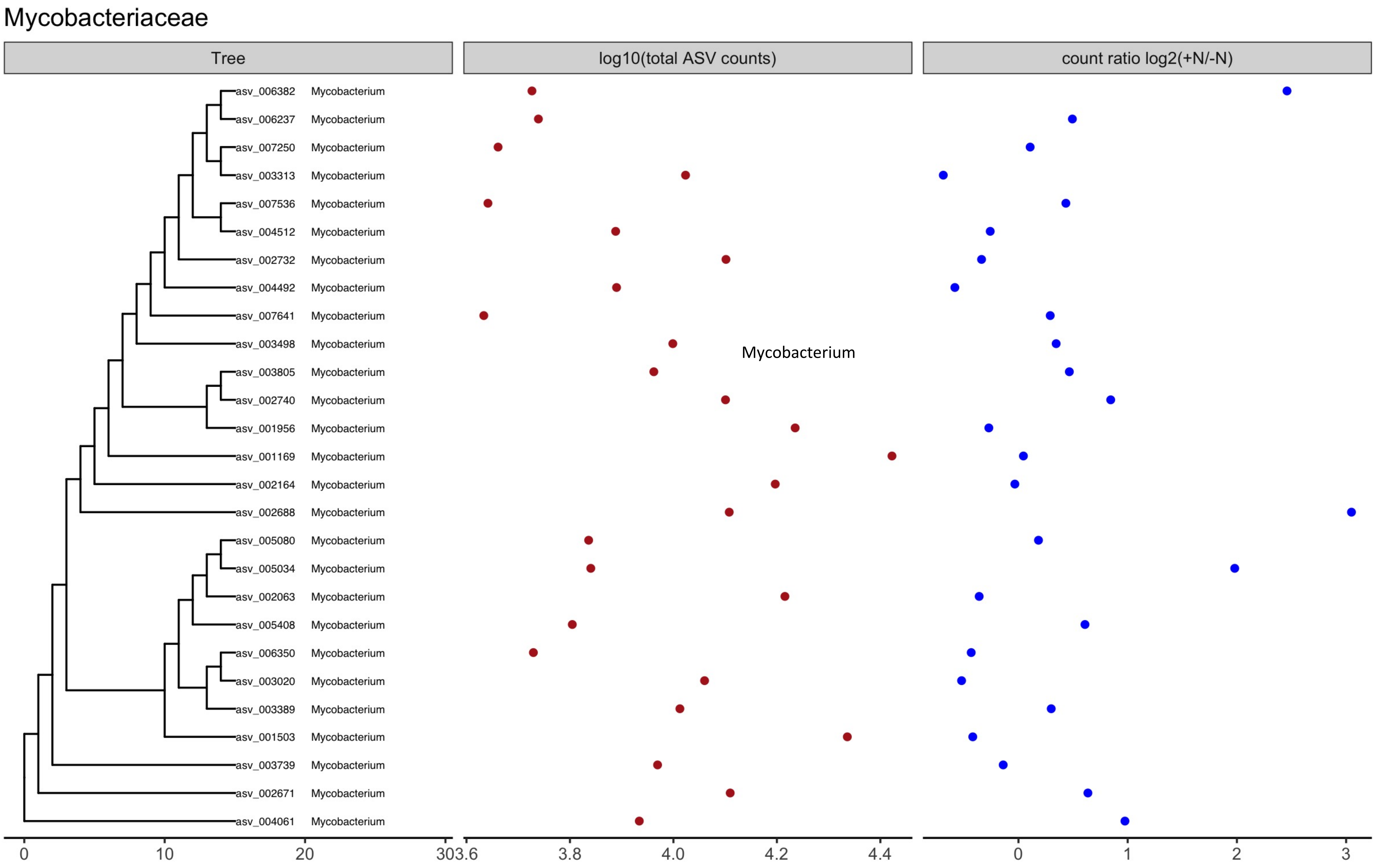

Mycobacteriaceae
Tree
log10(total ASV counts)
count ratio log2(+N/-N)
asv_006382 Mycobacterium
asv_006237 Mycobacterium
asv_007250 Mycobacterium
asv_003313 Mycobacterium
asv_007536 Mycobacterium
asv_004512 Mycobacterium
asv_002732 Mycobacterium
asv_004492 Mycobacterium
asv_007641 Mycobacterium
asv_003498 Mycobacterium
asv_003805 Mycobacterium
asv_002740 Mycobacterium
asv_001956 Mycobacterium
asv_001169 Mycobacterium
asv_002164 Mycobacterium
asv_002688 Mycobacterium
asv_005080 Mycobacterium
asv_005034 Mycobacterium
asv_002063 Mycobacterium
asv_005408 Mycobacterium
asv_006350 Mycobacterium
asv_003020 Mycobacterium
asv_003389 Mycobacterium
asv_001503 Mycobacterium
asv_003739 Mycobacterium
asv_002671 Mycobacterium
asv_004061 Mycobacterium
Mycobacterium
0
10
20
30
3.6
3.8
4.0
4.2
4.4
0
1
2
3

LWQ8

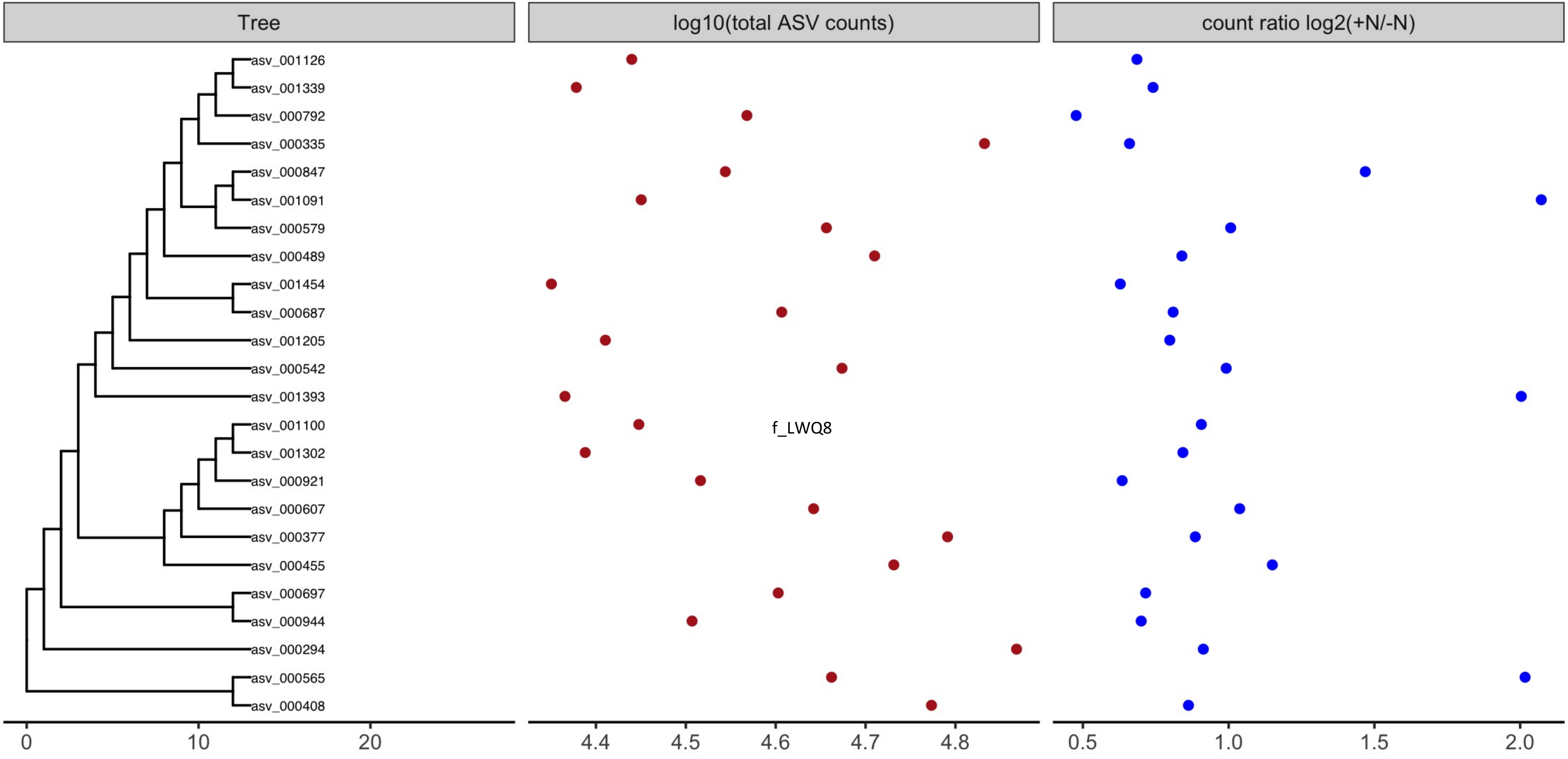
Tree
log10(total ASV counts)
count ratio log2(+N/-N)
asv_001126
asv_001339
asv_000792
asv_000335
asv_000847
asv_001091
asv_000579
asv_000489
asv_001454
asv_000687
asv_001205
asv_000542
asv_001393
asv_001100
asv_001302
asv_000921
asv_000607
asv_000377
asv_000455
asv_000697
asv_000944
asv_000294
asv_000565
asv_000408
f_LWQ8
0
10
20
4.4
4.5
4.6
4.7
4.8
0.5
1.0
1.5
2.0

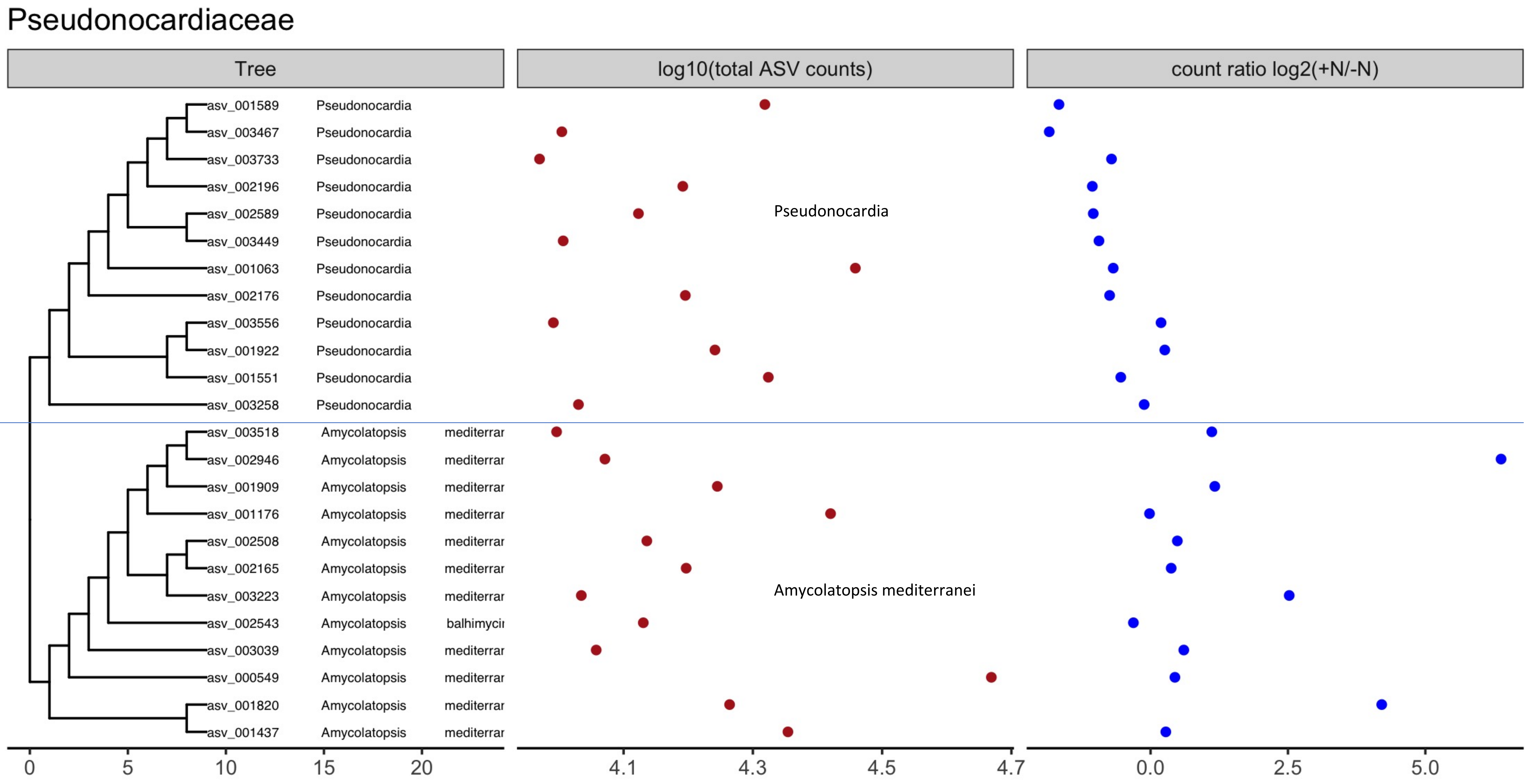

Pseudonocardiaceae
Tree
log10(total ASV counts)
count ratio log2(+N/-N)
asv_001589 Pseudonocardia
asv_003467 Pseudonocardia
asv_003733 Pseudonocardia
asv_002196 Pseudonocardia
asv_002589 Pseudonocardia
asv_003449 Pseudonocardia
asv_001063 Pseudonocardia
asv_002176 Pseudonocardia
asv_003556 Pseudonocardia
asv_001922 Pseudonocardia
asv_001551 Pseudonocardia
asv_003258 Pseudonocardia
asv_003518 Amycolatopsis mediterrar
asv_002946 Amycolatopsis mediterrar
asv_001909 Amycolatopsis mediterrar
asv_001176 Amycolatopsis mediterrar
asv_002508 Amycolatopsis mediterrar
asv_002165 Amycolatopsis mediterrar
asv_003223 Amycolatopsis mediterrar
asv_002543 Amycolatopsis balhimycir
asv_003039 Amycolatopsis mediterrar
asv_000549 Amycolatopsis mediterrar
asv_001820 Amycolatopsis mediterrar
asv_001437 Amycolatopsis mediterrar
Pseudonocardia
Amycolatopsis mediterranei
0
5
10
15
20
4.1
4.3
4.5
4.7
0.0
2.5
5.0

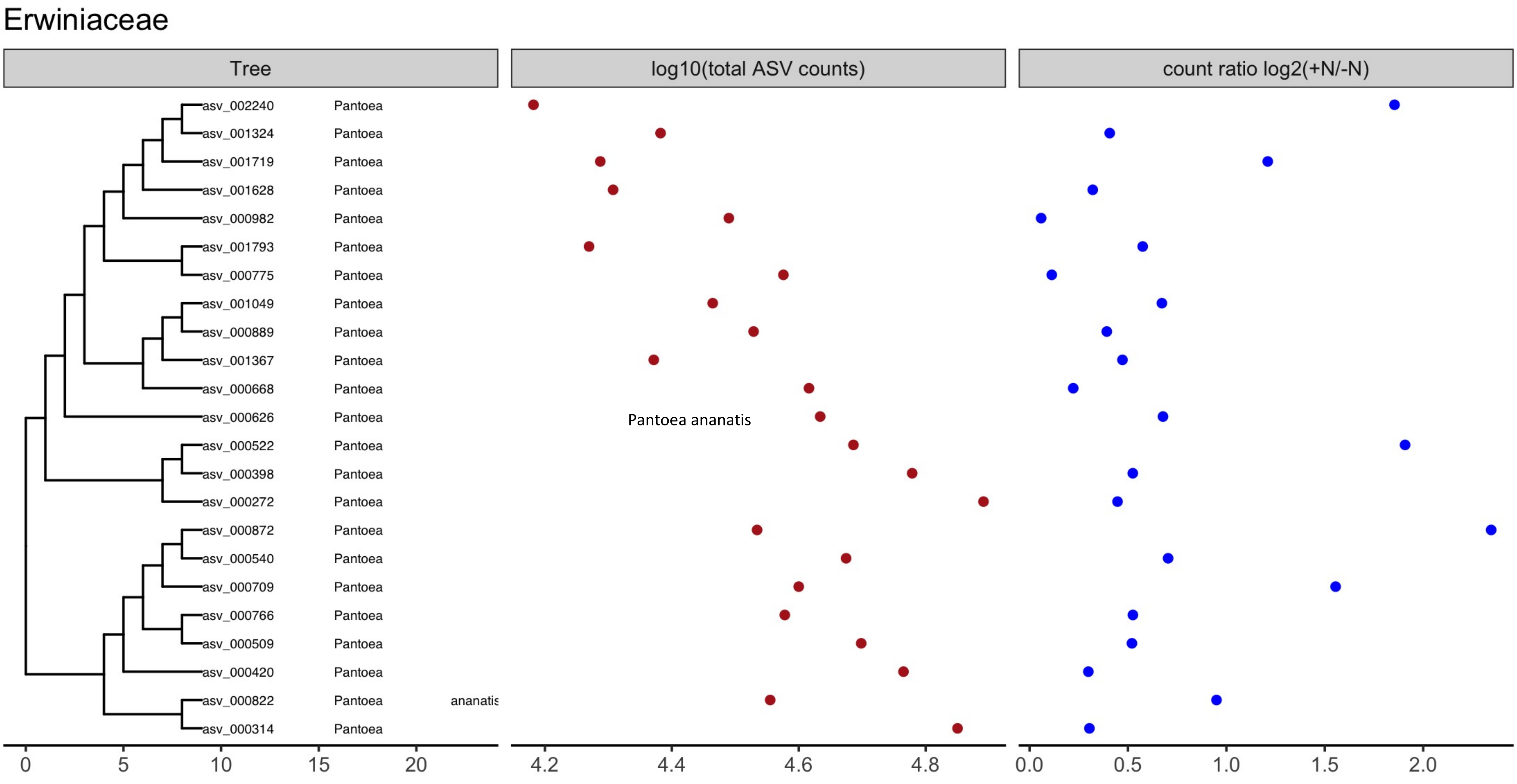

Erwiniaceae
Tree
log10(total ASV counts)
count ratio log2(+N/-N)
asv_002240 Pantoea
asv_001324 Pantoea
asv_001719 Pantoea
asv_001628 Pantoea
asv_000982 Pantoea
asv_001793 Pantoea
asv_000775 Pantoea
asv_001049 Pantoea
asv_000889 Pantoea
asv_001367 Pantoea
asv_000668 Pantoea
asv_000626 Pantoea
Pantoea ananatis
asv_000522 Pantoea
asv_000398 Pantoea
asv_000272 Pantoea
asv_000872 Pantoea
asv_000540 Pantoea
asv_000709 Pantoea
asv_000766 Pantoea
asv_000509 Pantoea
asv_000420 Pantoea
asv_000822 Pantoea ananatis
asv_000314 Pantoea
0 5 10 15 20
4.2 4.4 4.6 4.8
0.0 0.5 1.0 1.5 2.0

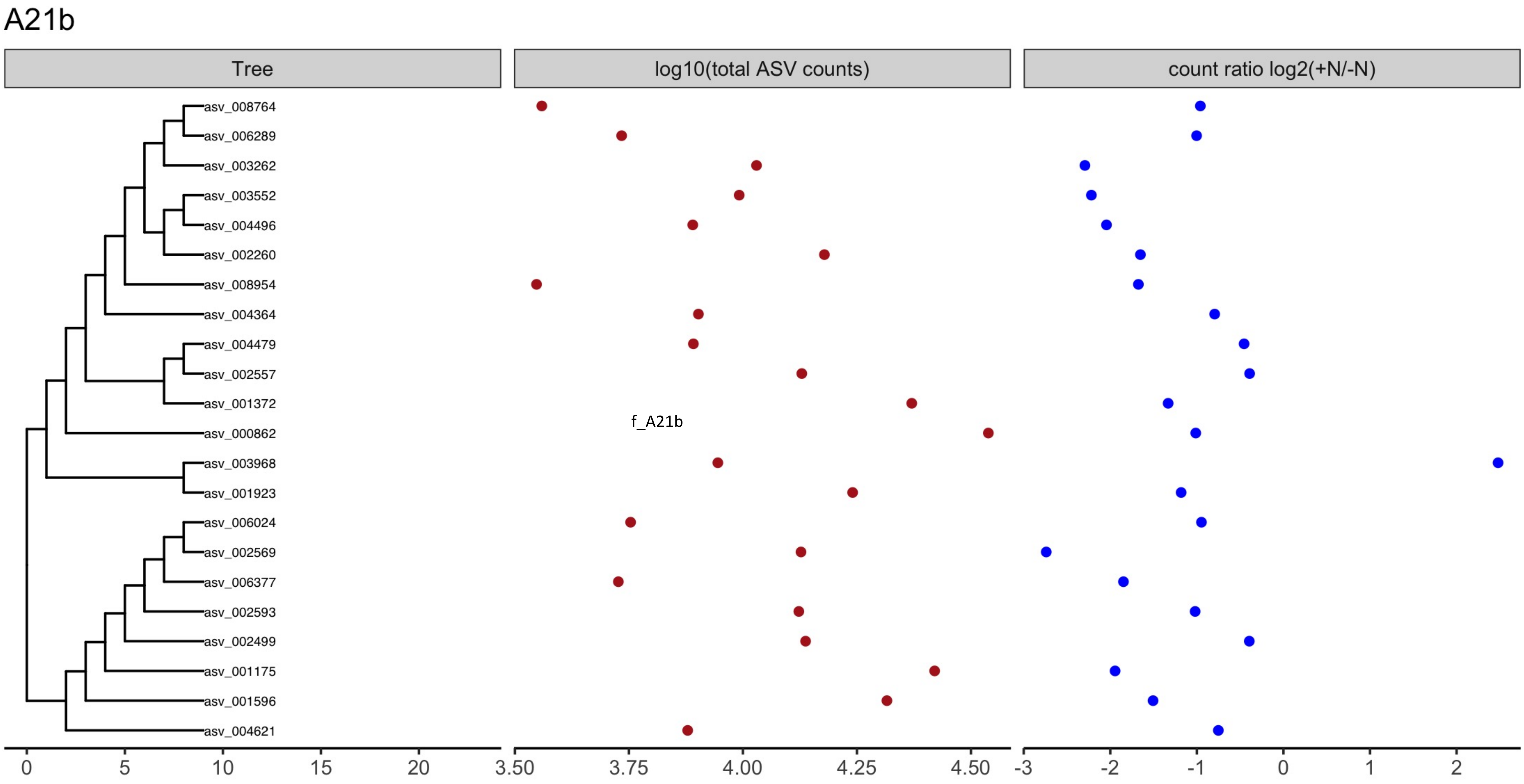
A21b
Tree
log10(total ASV counts)
count ratio log2(+N/-N)
asv_008764
asv_006289
asv_003262
asv_003552
asv_004496
asv_002260
asv_008954
asv_004364
asv_004479
asv_002557
asv_001372
asv_000862
asv_003968
asv_001923
asv_006024
asv_002569
asv_006377
asv_002593
asv_002499
asv_001175
asv_001596
asv_004621
f_A21b
0
5
10
15
20
3.50
3.75
4.00
4.25
4.50
-3
-2
-1
0
1
2

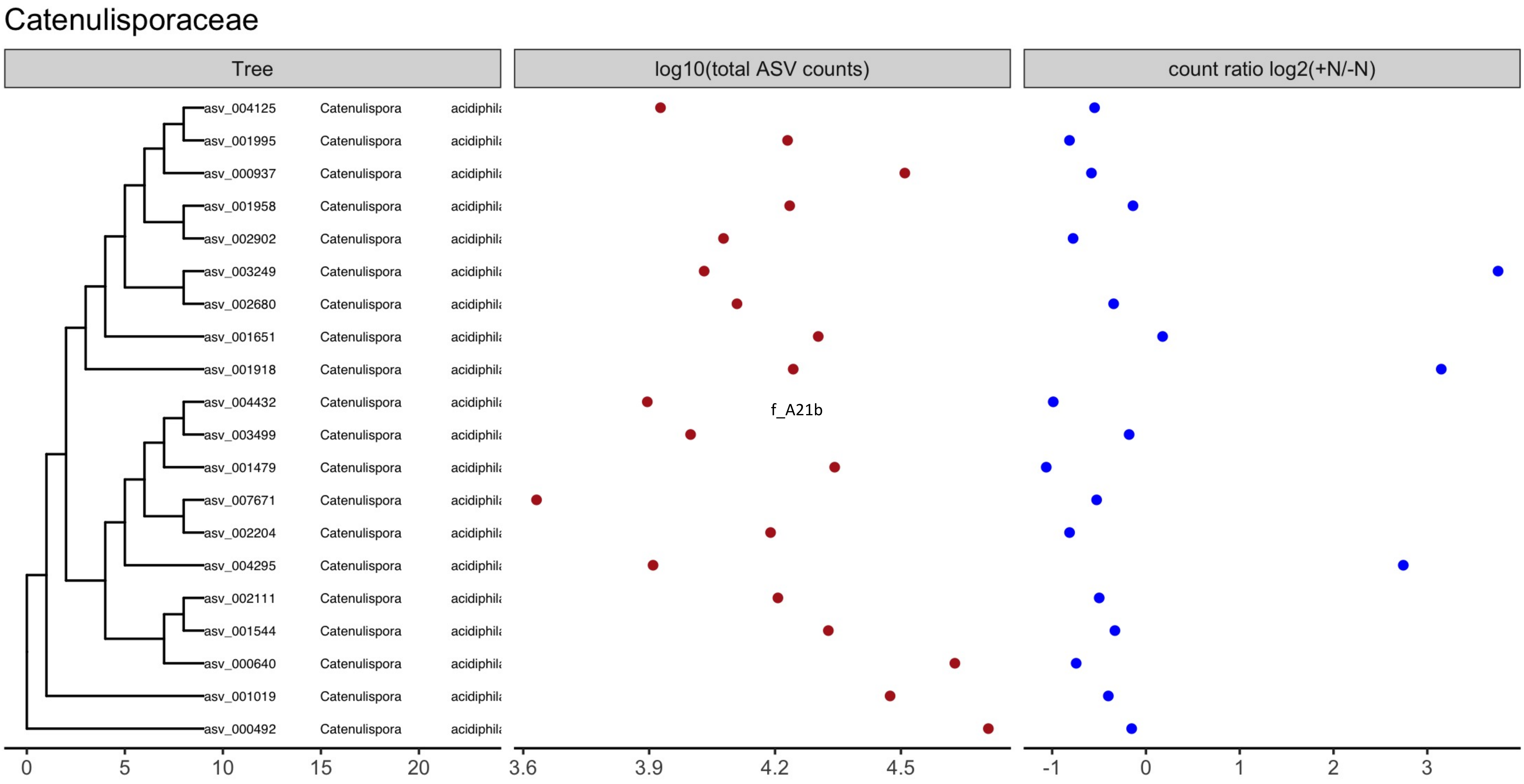
Catenulisporaceae
Tree
log10(total ASV counts)
count ratio log2(+N/-N)
asv_004125 Catenulispora acidiphil
asv_001995 Catenulispora acidiphil
asv_000937 Catenulispora acidiphil
asv_001958 Catenulispora acidiphil
asv_002902 Catenulispora acidiphil
asv_003249 Catenulispora acidiphil
asv_002680 Catenulispora acidiphil
asv_001651 Catenulispora acidiphil
asv_001918 Catenulispora acidiphil
asv_004432 Catenulispora acidiphil
asv_003499 Catenulispora acidiphil
asv_001479 Catenulispora acidiphil
asv_007671 Catenulispora acidiphil
asv_002204 Catenulispora acidiphil
asv_004295 Catenulispora acidiphil
asv_002111 Catenulispora acidiphil
asv_001544 Catenulispora acidiphil
asv_000640 Catenulispora acidiphil
asv_001019 Catenulispora acidiphil
asv_000492 Catenulispora acidiphil
f_A21b
0 5 10 15 20
3.6 3.9 4.2 4.5
-1 0 1 2 3

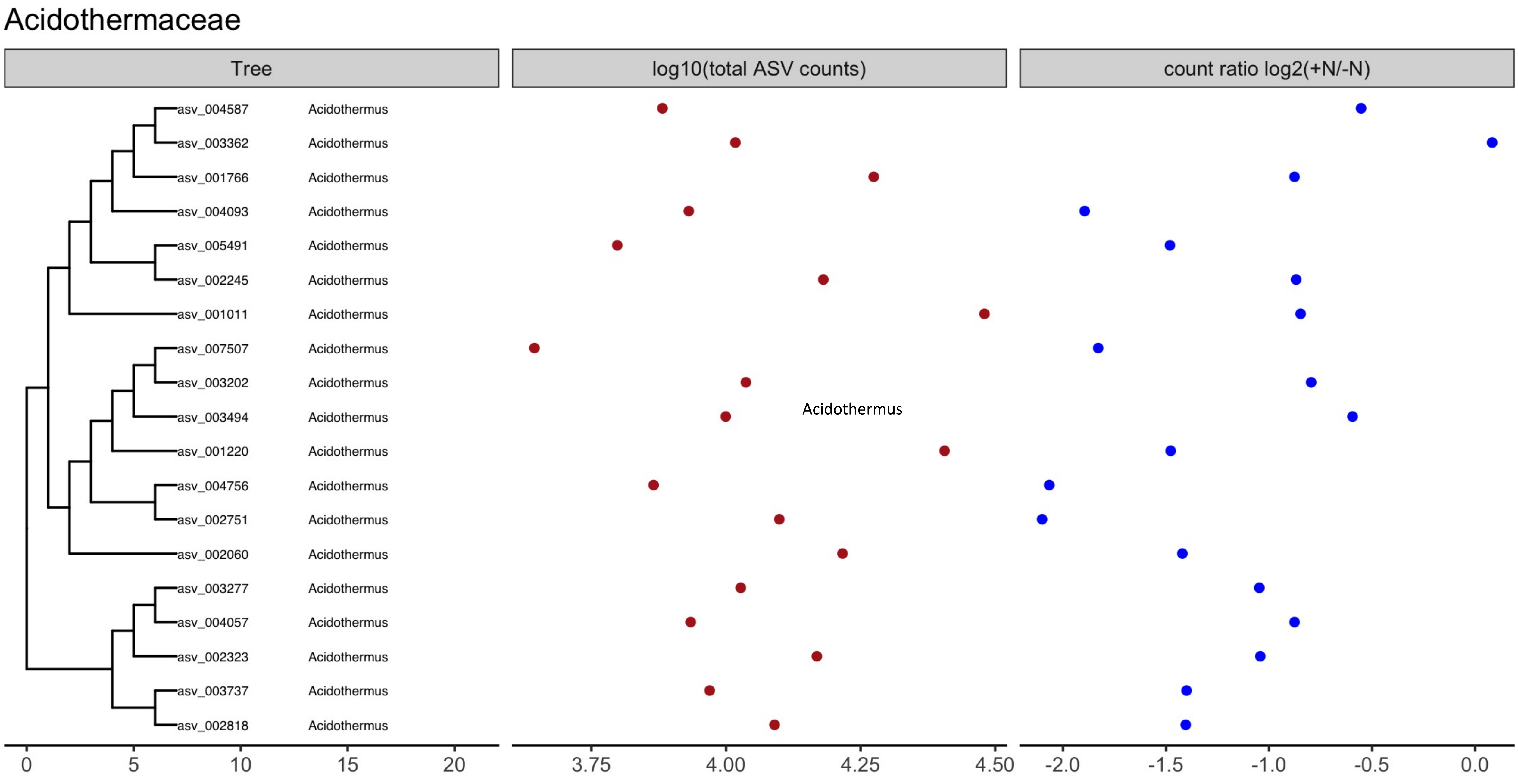
Acidothermaceae
Tree
log10(total ASV counts)
count ratio log2(+N/-N)
asv_004587 Acidothermus
asv_003362 Acidothermus
asv_001766 Acidothermus
asv_004093 Acidothermus
asv_005491 Acidothermus
asv_002245 Acidothermus
asv_001011 Acidothermus
asv_007507 Acidothermus
asv_003202 Acidothermus
asv_003494 Acidothermus
asv_001220 Acidothermus
asv_004756 Acidothermus
asv_002751 Acidothermus
asv_002060 Acidothermus
asv_003277 Acidothermus
asv_004057 Acidothermus
asv_002323 Acidothermus
asv_003737 Acidothermus
asv_002818 Acidothermus
Acidothermus
0 5 10 15 20
3.75 4.00 4.25 4.50
-2.0 -1.5 -1.0 -0.5 0.0

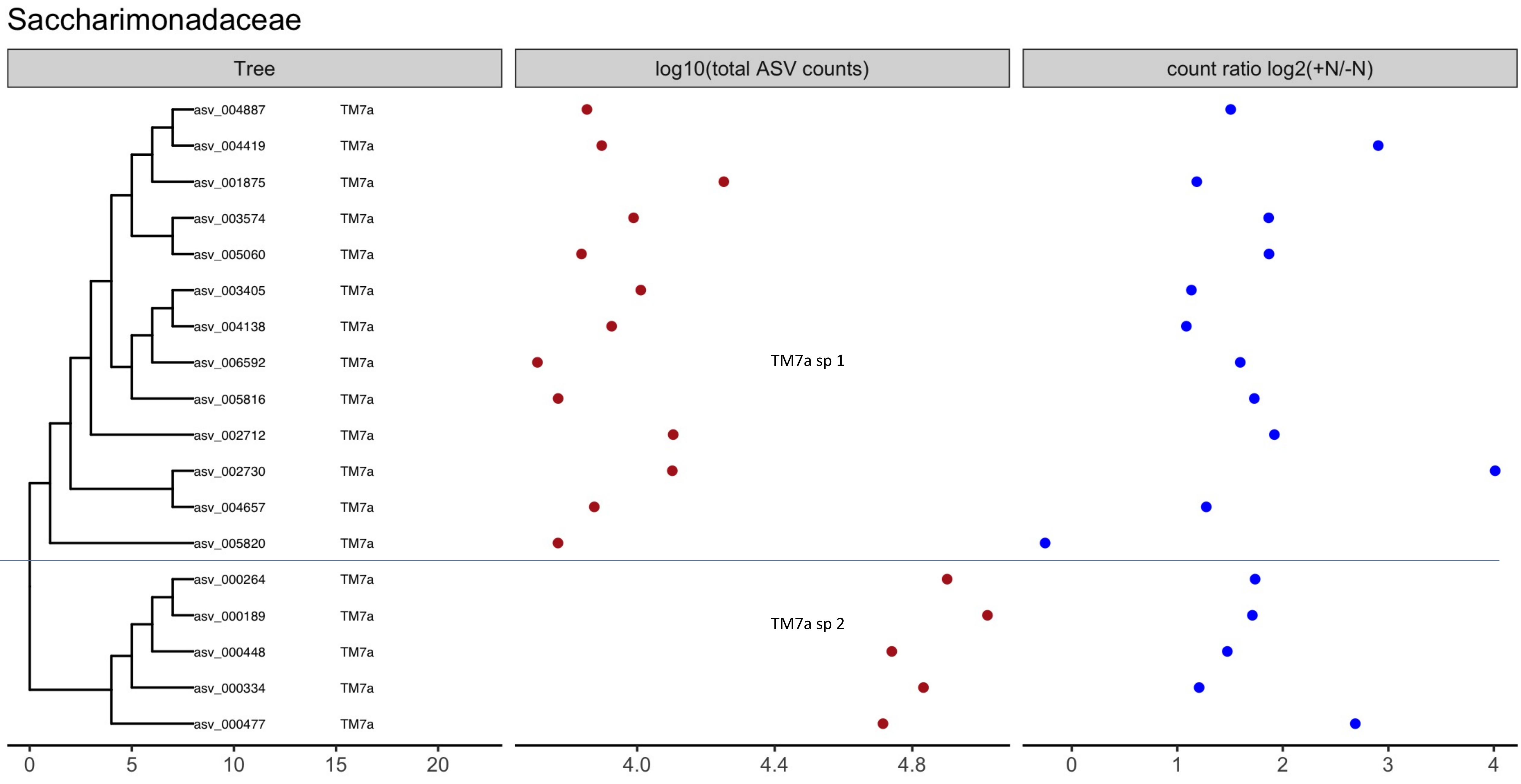
Saccharimonadaceae
Tree
log10(total ASV counts)
count ratio log2(+N/-N)
asv_004887 TM7a
asv_004419 TM7a
asv_001875 TM7a
asv_003574 TM7a
asv_005060 TM7a
asv_003405 TM7a
asv_004138 TM7a
asv_006592 TM7a
asv_005816 TM7a
asv_002712 TM7a
asv_002730 TM7a
asv_004657 TM7a
asv_005820 TM7a
asv_000264 TM7a
asv_000189 TM7a
asv_000448 TM7a
asv_000334 TM7a
asv_000477 TM7a
TM7a sp 1
TM7a sp 2
0
5
10
15
20
4.0
4.4
4.8
0
1
2
3
4

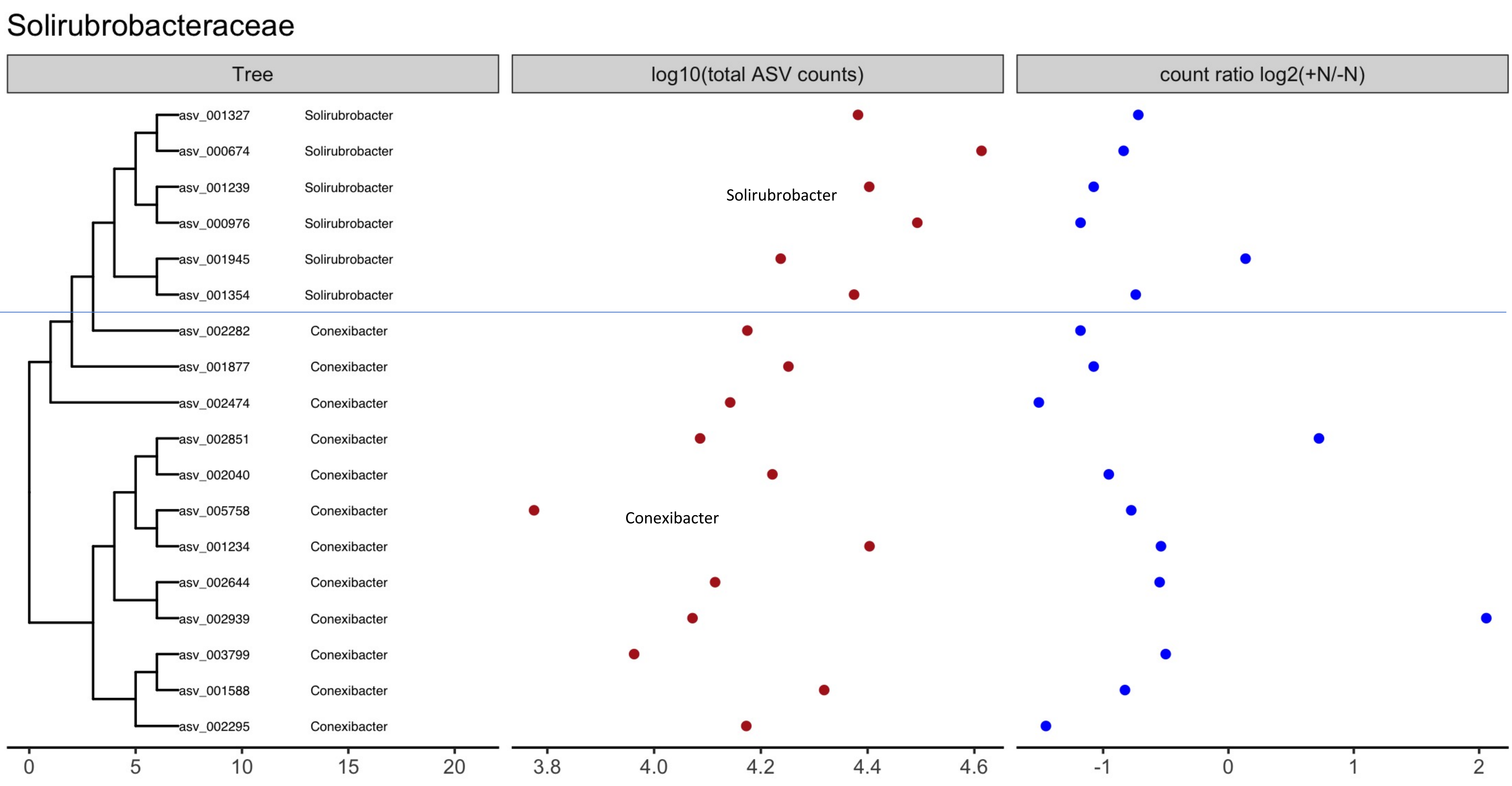
Solirubrobacteraceae
Tree
log10(total ASV counts)
count ratio log2(+N/-N)
asv_001327 Solirubrobacter
asv_000674 Solirubrobacter
asv_001239 Solirubrobacter
asv_000976 Solirubrobacter
asv_001945 Solirubrobacter
asv_001354 Solirubrobacter
asv_002282 Conexibacter
asv_001877 Conexibacter
asv_002474 Conexibacter
asv_002851 Conexibacter
asv_002040 Conexibacter
asv_005758 Conexibacter
asv_001234 Conexibacter
asv_002644 Conexibacter
asv_002939 Conexibacter
asv_003799 Conexibacter
asv_001588 Conexibacter
asv_002295 Conexibacter
Solirubrobacter
Conexibacter
0 5 10 15 20
3.8 4.0 4.2 4.4 4.6
-1 0 1 2

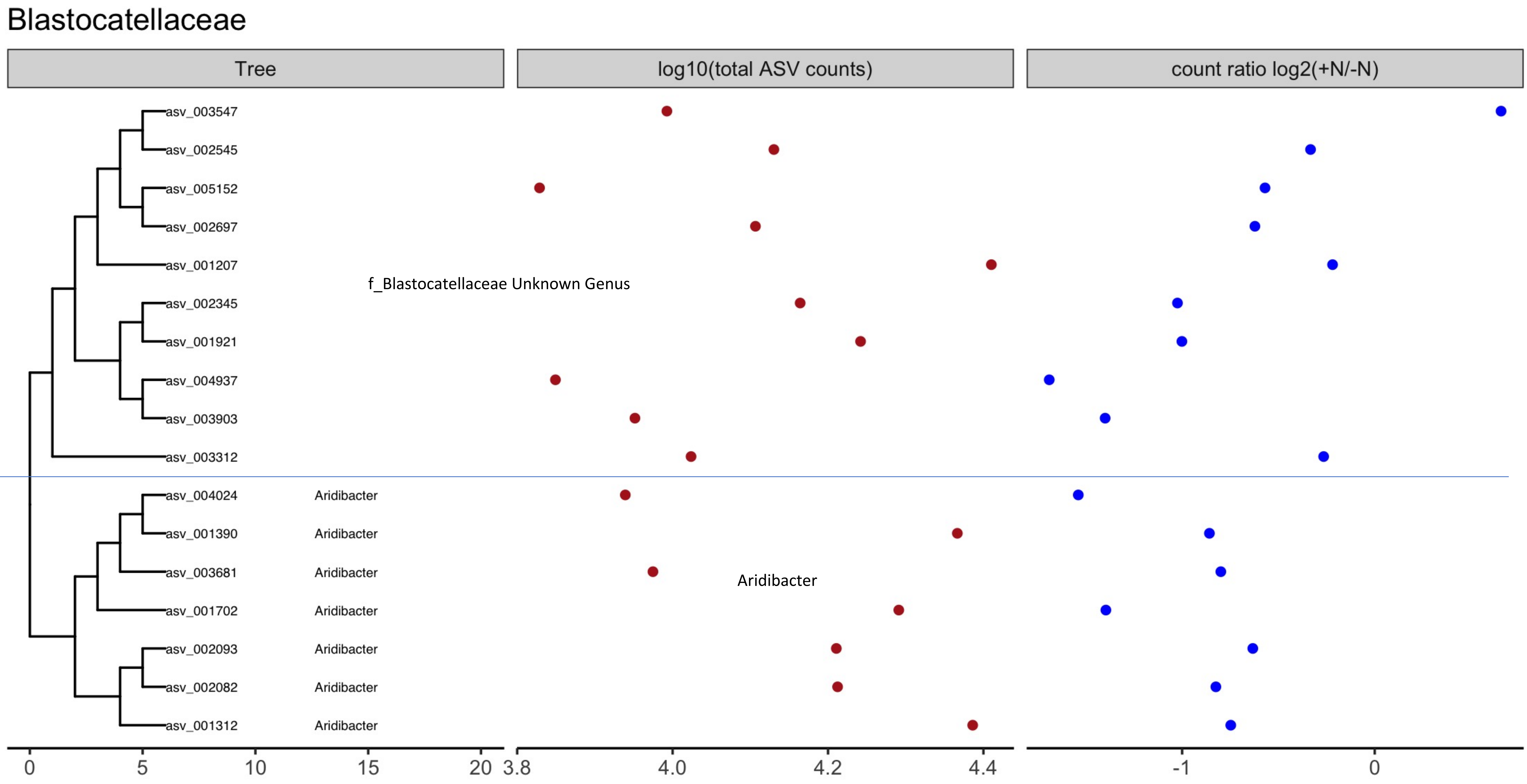

Blastocatellaceae
Tree
log10(total ASV counts)
count ratio log2(+N/-N)
asv_003547
asv_002545
asv_005152
asv_002697
asv_001207
f_Blastocatellaceae Unknown Genus
asv_002345
asv_001921
asv_004937
asv_003903
asv_003312
asv_004024 Aridibacter
asv_001390 Aridibacter
asv_003681 Aridibacter
Aridibacter
asv_001702 Aridibacter
asv_002093 Aridibacter
asv_002082 Aridibacter
asv_001312 Aridibacter
0 5 10 15 20
3.8 4.0 4.2 4.4
-1 0

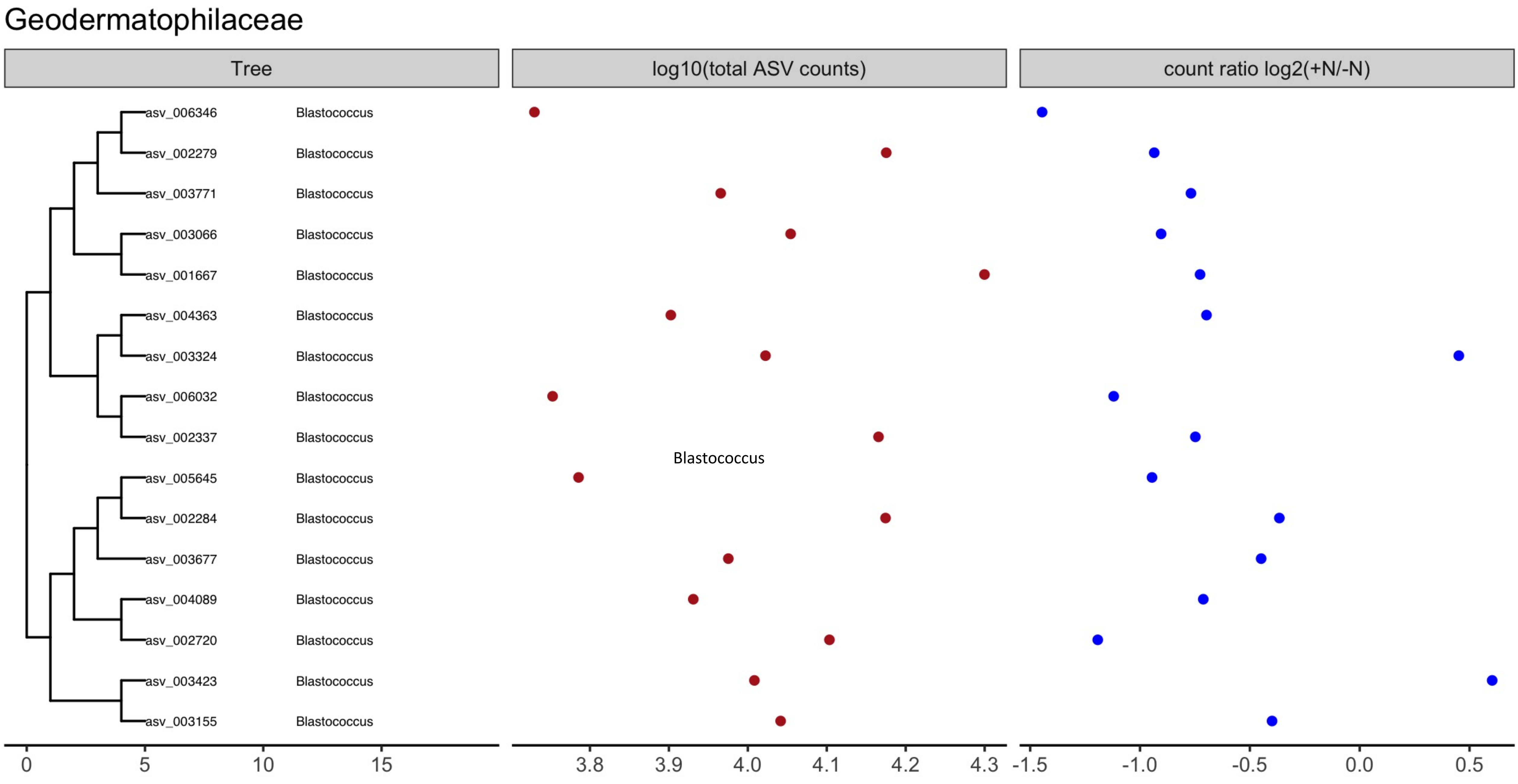

Geodermatophilaceae
Tree
log10(total ASV counts)
count ratio log2(+N/-N)
asv_006346 Blastococcus
asv_002279 Blastococcus
asv_003771 Blastococcus
asv_003066 Blastococcus
asv_001667 Blastococcus
asv_004363 Blastococcus
asv_003324 Blastococcus
asv_006032 Blastococcus
asv_002337 Blastococcus
Blastococcus
asv_005645 Blastococcus
asv_002284 Blastococcus
asv_003677 Blastococcus
asv_004089 Blastococcus
asv_002720 Blastococcus
asv_003423 Blastococcus
asv_003155 Blastococcus
0 5 10 15
3.8 3.9 4.0 4.1 4.2 4.3
-1.5 -1.0 -0.5 0.0 0.5

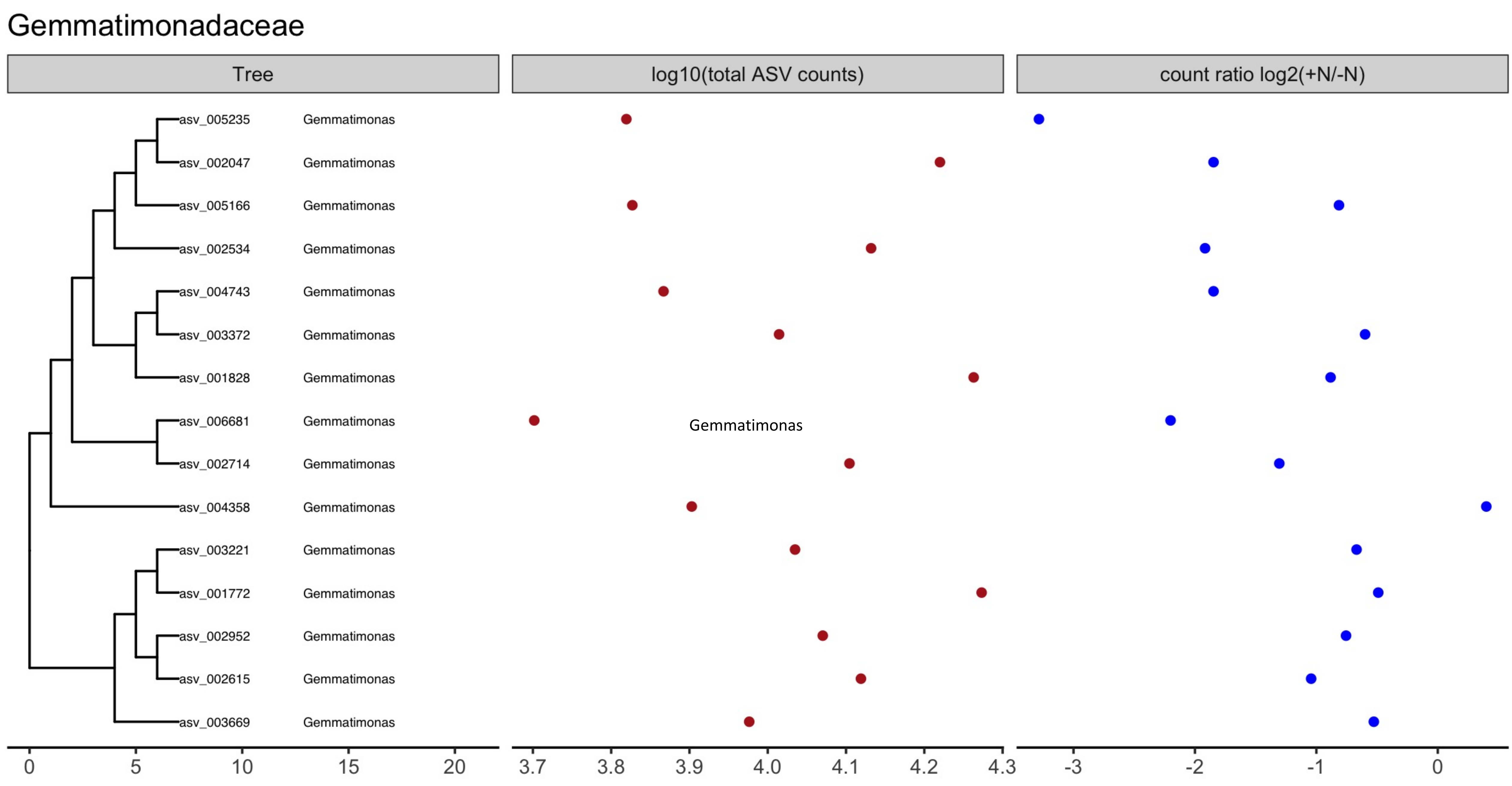
Gemmatimonadaceae
Tree
log10(total ASV counts)
count ratio log2(+N/-N)
asv_005235 Gemmatimonas
asv_002047 Gemmatimonas
asv_005166 Gemmatimonas
asv_002534 Gemmatimonas
asv_004743 Gemmatimonas
asv_003372 Gemmatimonas
asv_001828 Gemmatimonas
asv_006681 Gemmatimonas
asv_002714 Gemmatimonas
asv_004358 Gemmatimonas
asv_003221 Gemmatimonas
asv_001772 Gemmatimonas
asv_002952 Gemmatimonas
asv_002615 Gemmatimonas
asv_003669 Gemmatimonas
Gemmatimonas
0 5 10 15 20
3.7 3.8 3.9 4.0 4.1 4.2 4.3
-3 -2 -1 0

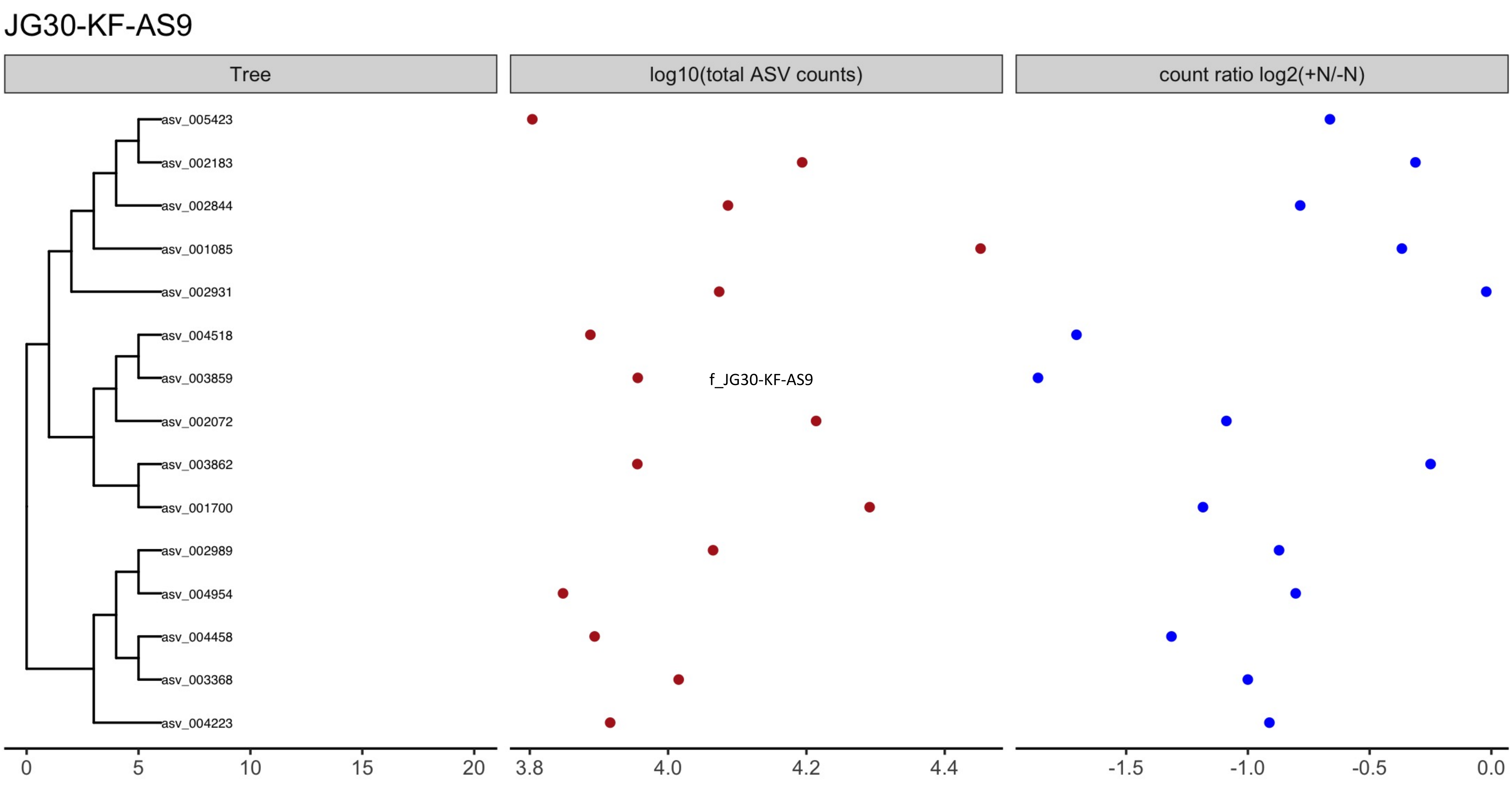

JG30-KF-AS9
Tree
log10(total ASV counts)
count ratio log2(+N/-N)
asv_005423
asv_002183
asv_002844
asv_001085
asv_002931
asv_004518
asv_003859
asv_002072
asv_003862
asv_001700
asv_002989
asv_004954
asv_004458
asv_003368
asv_004223
f_JG30-KF-AS9
0
5
10
15
20
3.8
4.0
4.2
4.4
-1.5
-1.0
-0.5
0.0

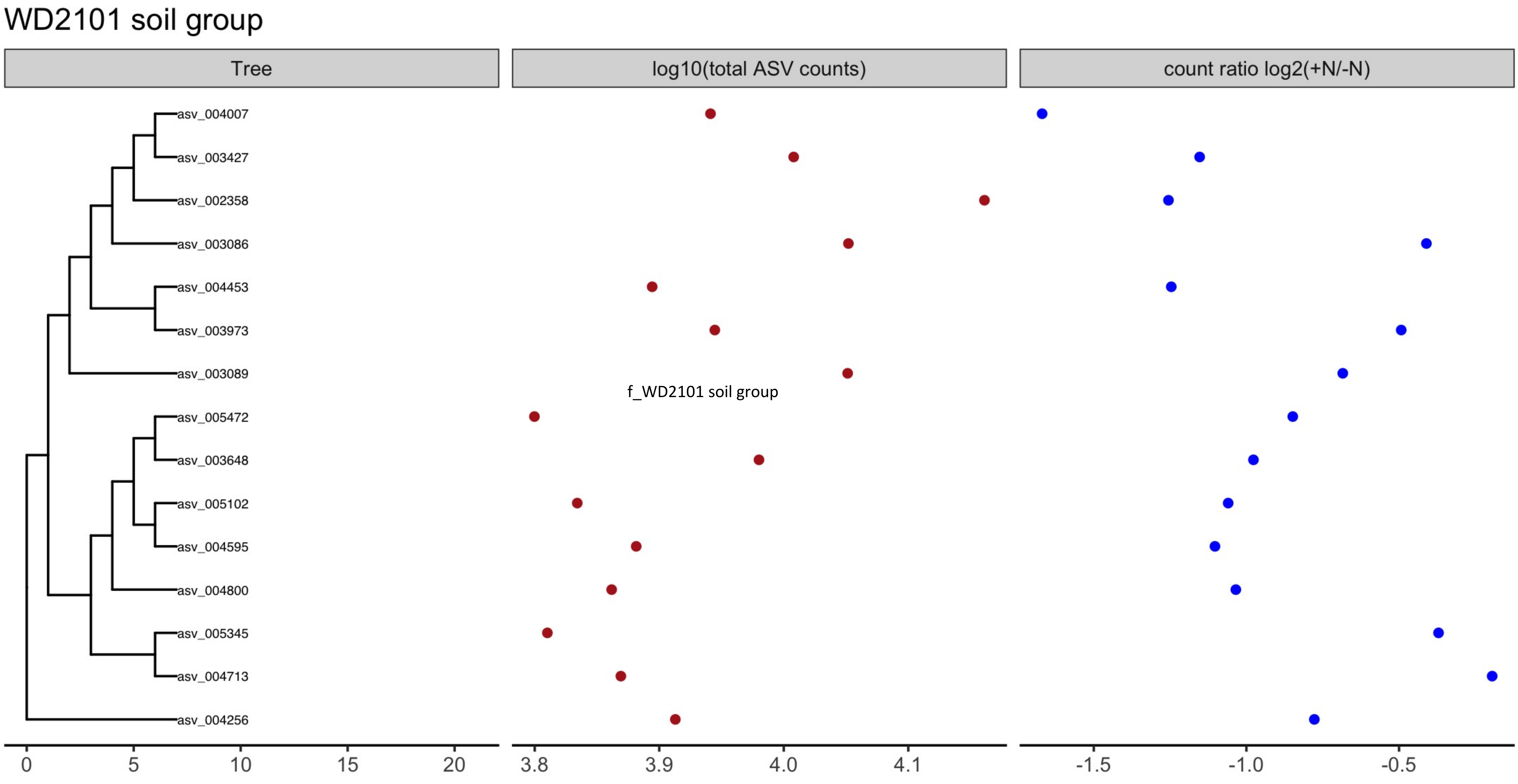

f_WD2101 soil group

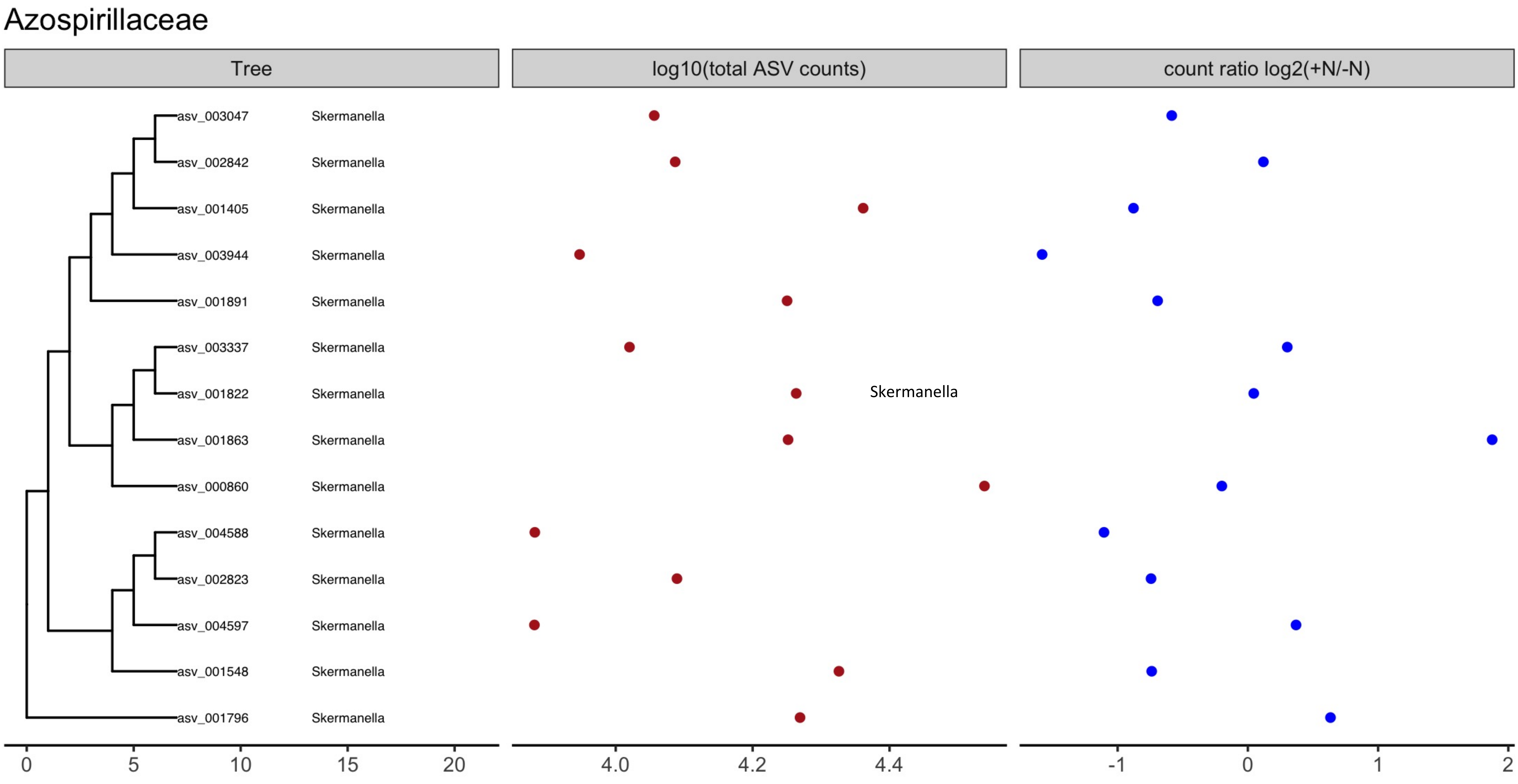

Azospirillaceae
Tree
log10(total ASV counts)
count ratio log2(+N/-N)
asv_003047 Skermanella
asv_002842 Skermanella
asv_001405 Skermanella
asv_003944 Skermanella
asv_001891 Skermanella
asv_003337 Skermanella
asv_001822 Skermanella
asv_001863 Skermanella
asv_000860 Skermanella
asv_004588 Skermanella
asv_002823 Skermanella
asv_004597 Skermanella
asv_001548 Skermanella
asv_001796 Skermanella
Skermanella
0 5 10 15 20
4.0 4.2 4.4
-1 0 1 2

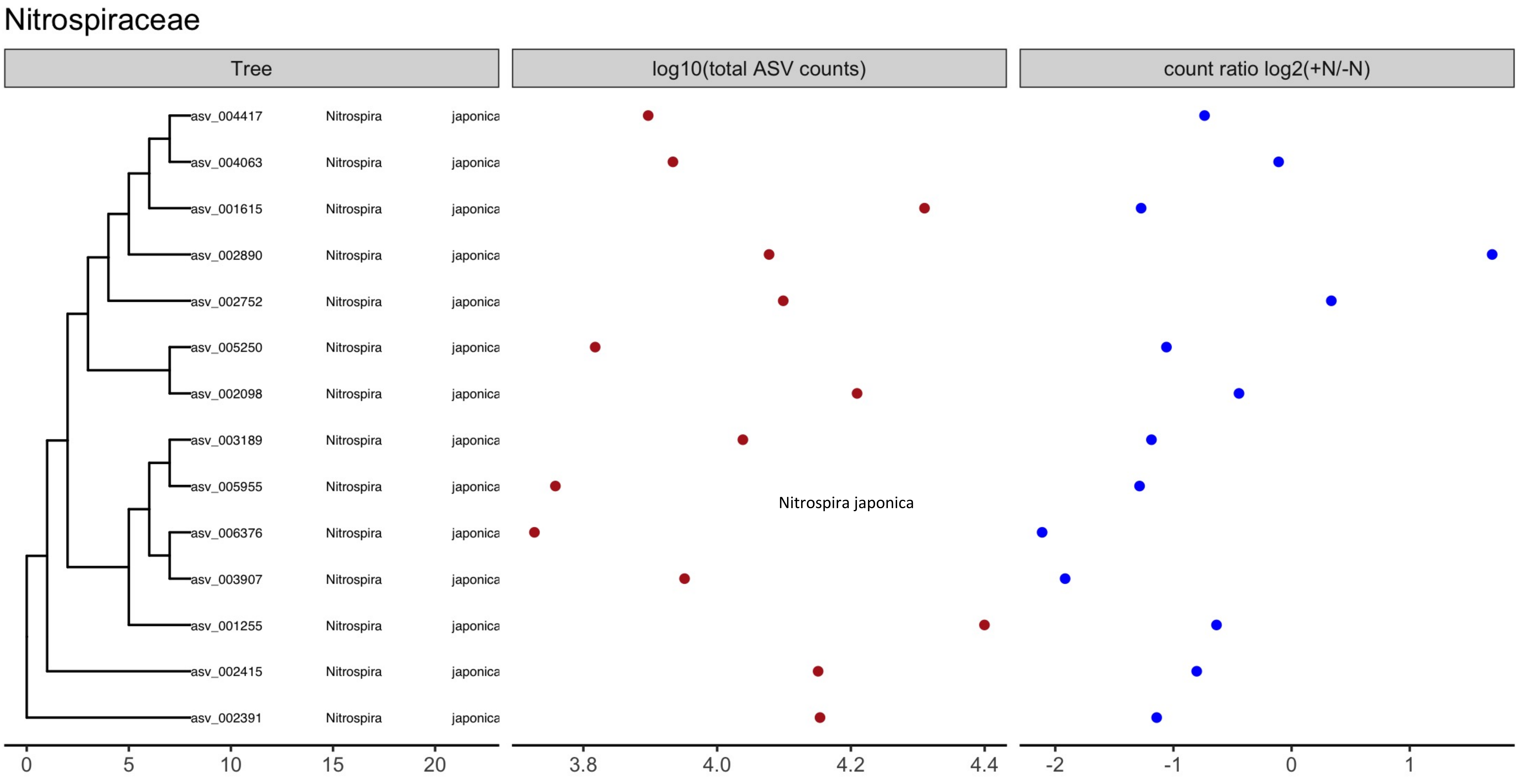
Nitrospiraceae
Tree
log10(total ASV counts)
count ratio log2(+N/-N)
asv_004417 Nitrospira japonica
asv_004063 Nitrospira japonica
asv_001615 Nitrospira japonica
asv_002890 Nitrospira japonica
asv_002752 Nitrospira japonica
asv_005250 Nitrospira japonica
asv_002098 Nitrospira japonica
asv_003189 Nitrospira japonica
asv_005955 Nitrospira japonica
asv_006376 Nitrospira japonica
asv_003907 Nitrospira japonica
asv_001255 Nitrospira japonica
asv_002415 Nitrospira japonica
asv_002391 Nitrospira japonica
Nitrospira japonica
0 5 10 15 20
3.8 4.0 4.2 4.4
-2 -1 0 1

Flavobacteriaceae

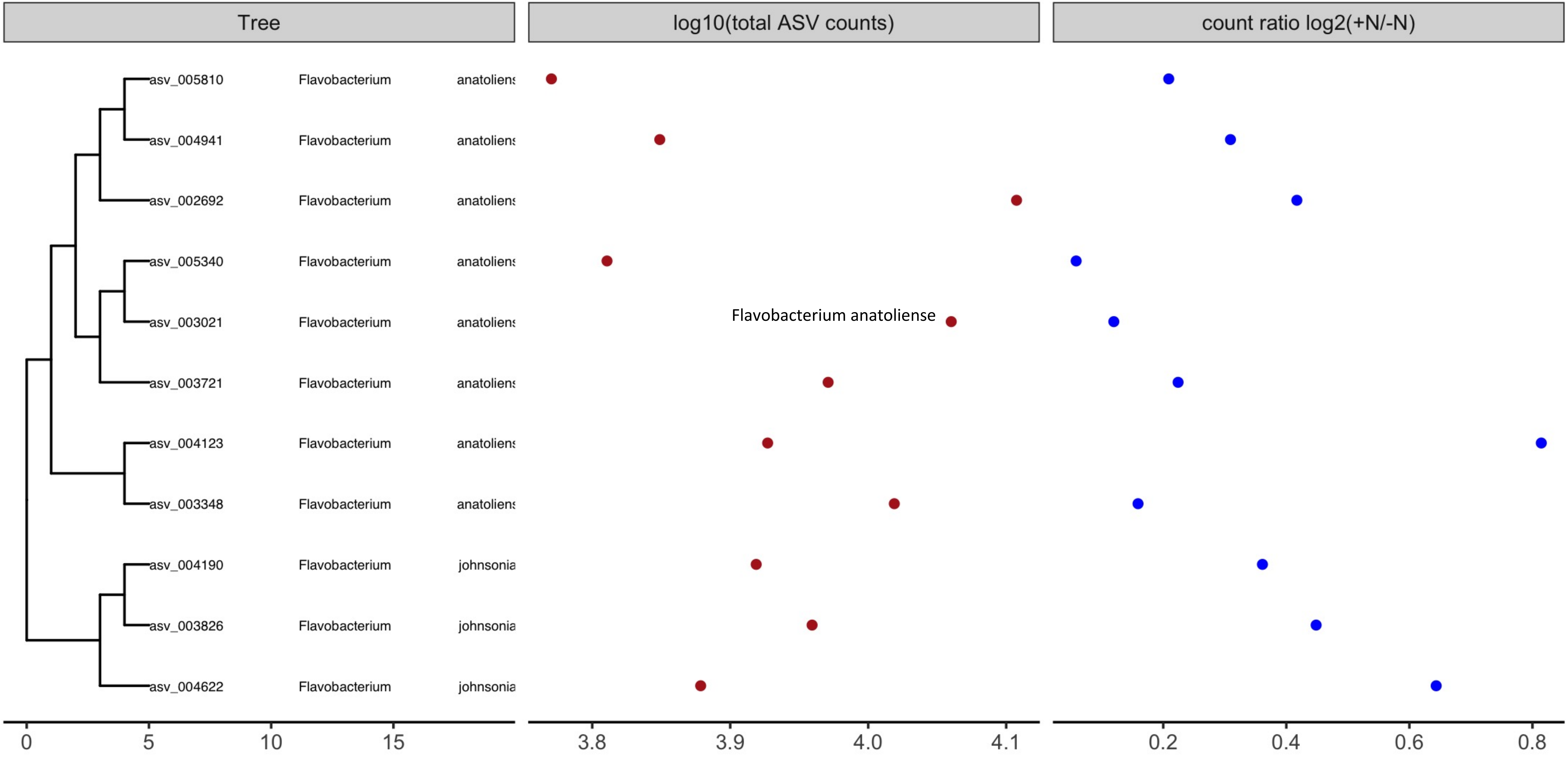

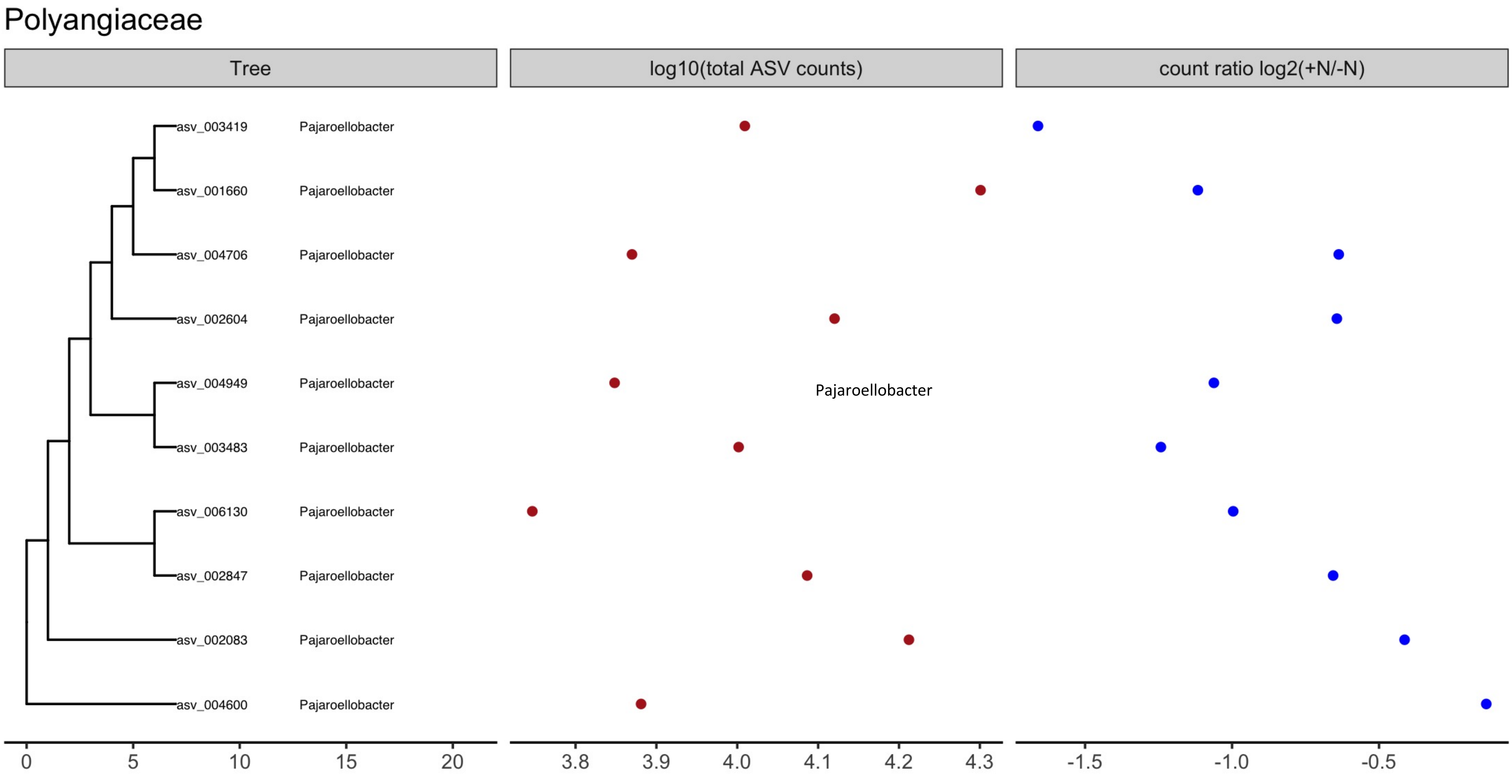
Polyangiaceae
Tree
log10(total ASV counts)
count ratio log2(+N/-N)
asv_003419 Pajaroellobacter
asv_001660 Pajaroellobacter
asv_004706 Pajaroellobacter
asv_002604 Pajaroellobacter
asv_004949 Pajaroellobacter
asv_003483 Pajaroellobacter
asv_006130 Pajaroellobacter
asv_002847 Pajaroellobacter
asv_002083 Pajaroellobacter
asv_004600 Pajaroellobacter
Pajaroellobacter
0
5
10
15
20
3.8
3.9
4.0
4.1
4.2
4.3
-1.5
-1.0
-0.5

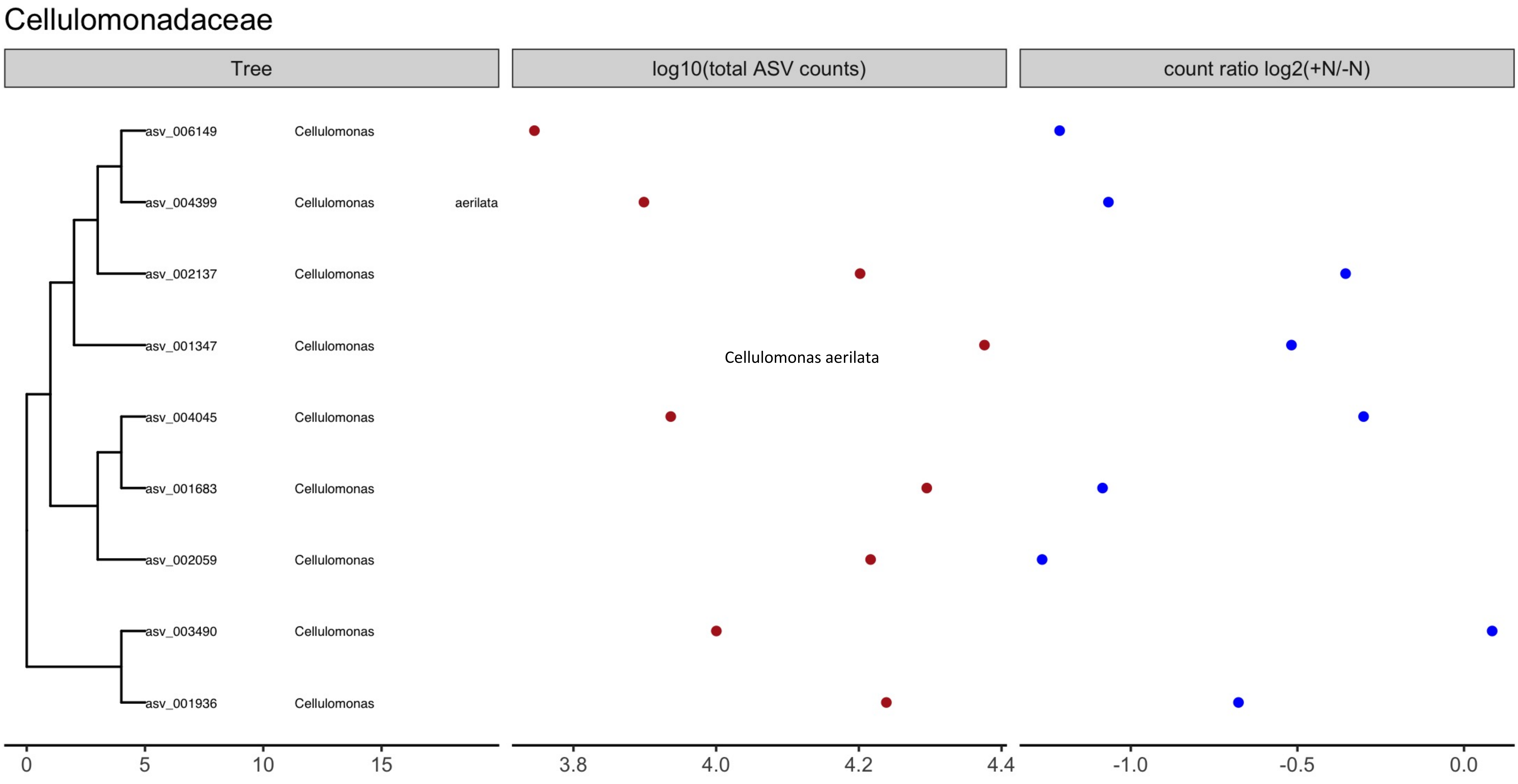
Cellulomonadaceae
Tree
log10(total ASV counts)
count ratio log2(+N/-N)
asv_006149 Cellulomonas
asv_004399 Cellulomonas aerilata
asv_002137 Cellulomonas
asv_001347 Cellulomonas
asv_004045 Cellulomonas
asv_001683 Cellulomonas
asv_002059 Cellulomonas
asv_003490 Cellulomonas
asv_001936 Cellulomonas
Cellulomonas aerilata
0 5 10 15
3.8 4.0 4.2 4.4
-1.0 -0.5 0.0

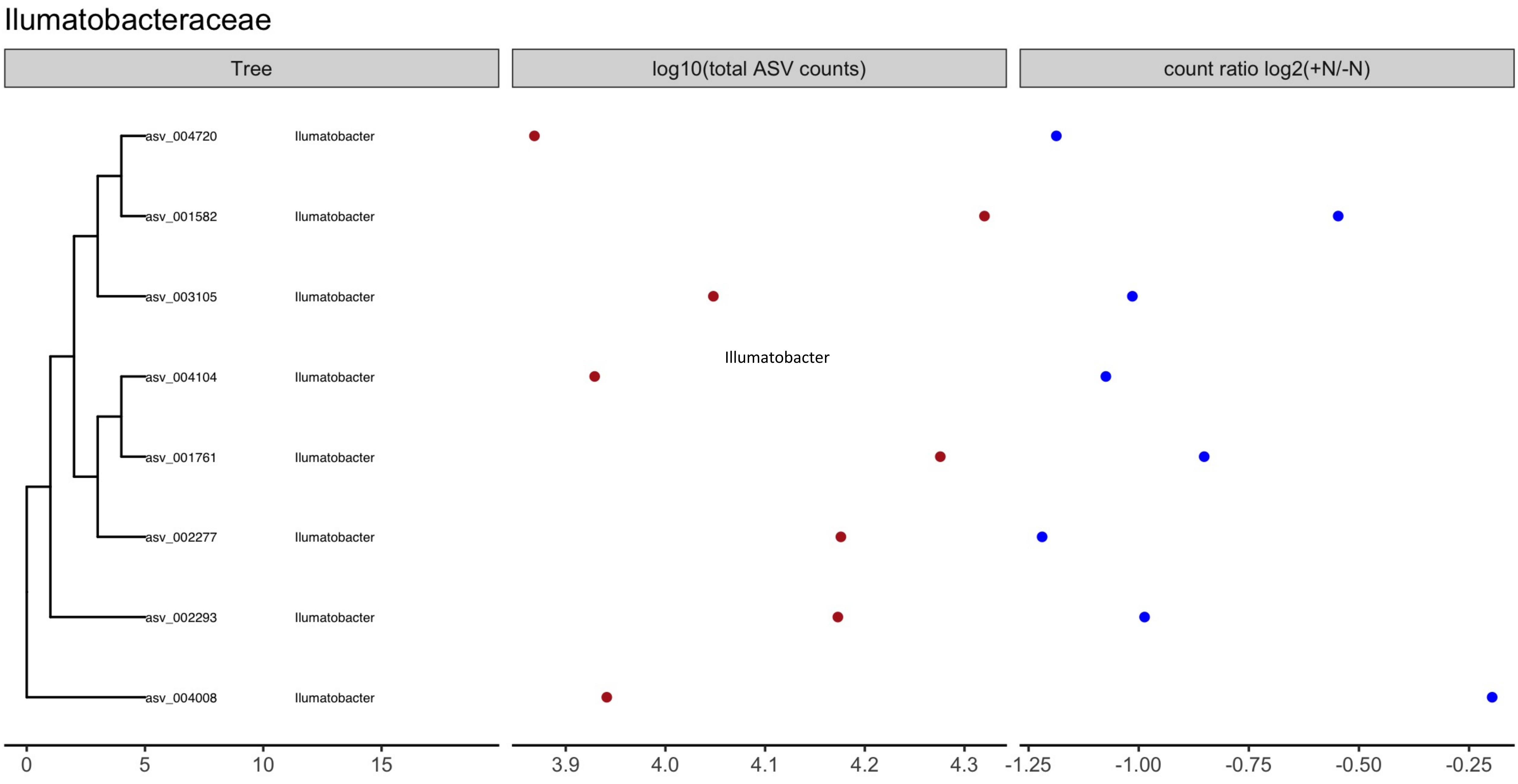

Illumatobacter

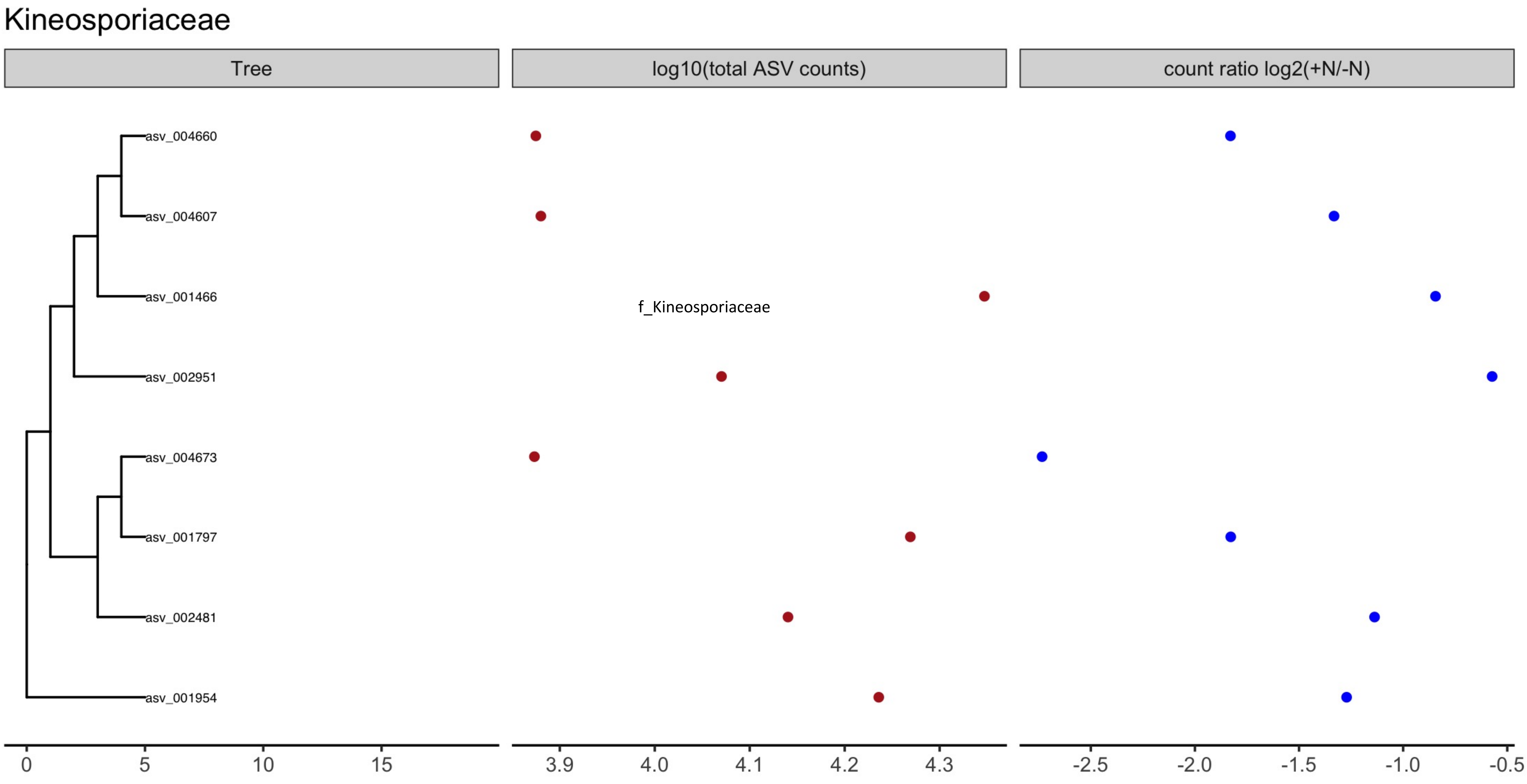

Kineosporiaceae
Tree
log10(total ASV counts)
count ratio log2(+N/-N)
asv_004660
asv_004607
asv_001466
asv_002951
asv_004673
asv_001797
asv_002481
asv_001954
f_Kineosporiaceae
0
5
10
15
3.9
4.0
4.1
4.2
4.3
-2.5
-2.0
-1.5
-1.0
-0.5

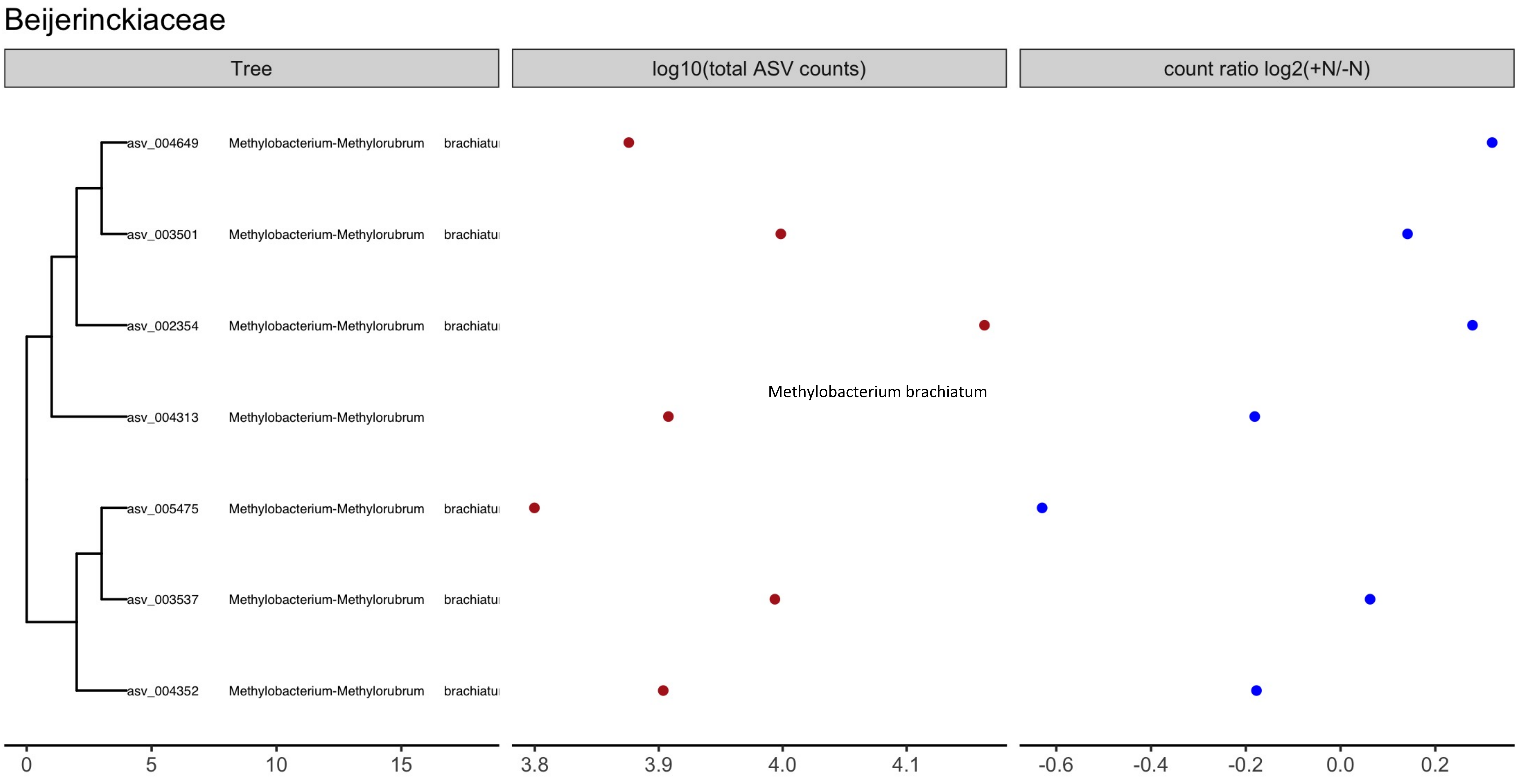

Beijerinckiaceae
Tree
log10(total ASV counts)
count ratio log2(+N/-N)
asv_004649 Methylobacterium-Methylorubrum brachiatu
asv_003501 Methylobacterium-Methylorubrum brachiatu
asv_002354 Methylobacterium-Methylorubrum brachiatu
asv_004313 Methylobacterium-Methylorubrum
asv_005475 Methylobacterium-Methylorubrum brachiatu
asv_003537 Methylobacterium-Methylorubrum brachiatu
asv_004352 Methylobacterium-Methylorubrum brachiatu
Methylobacterium brachiatum
0 5 10 15
3.8 3.9 4.0 4.1
-0.6 -0.4 -0.2 0.0 0.2

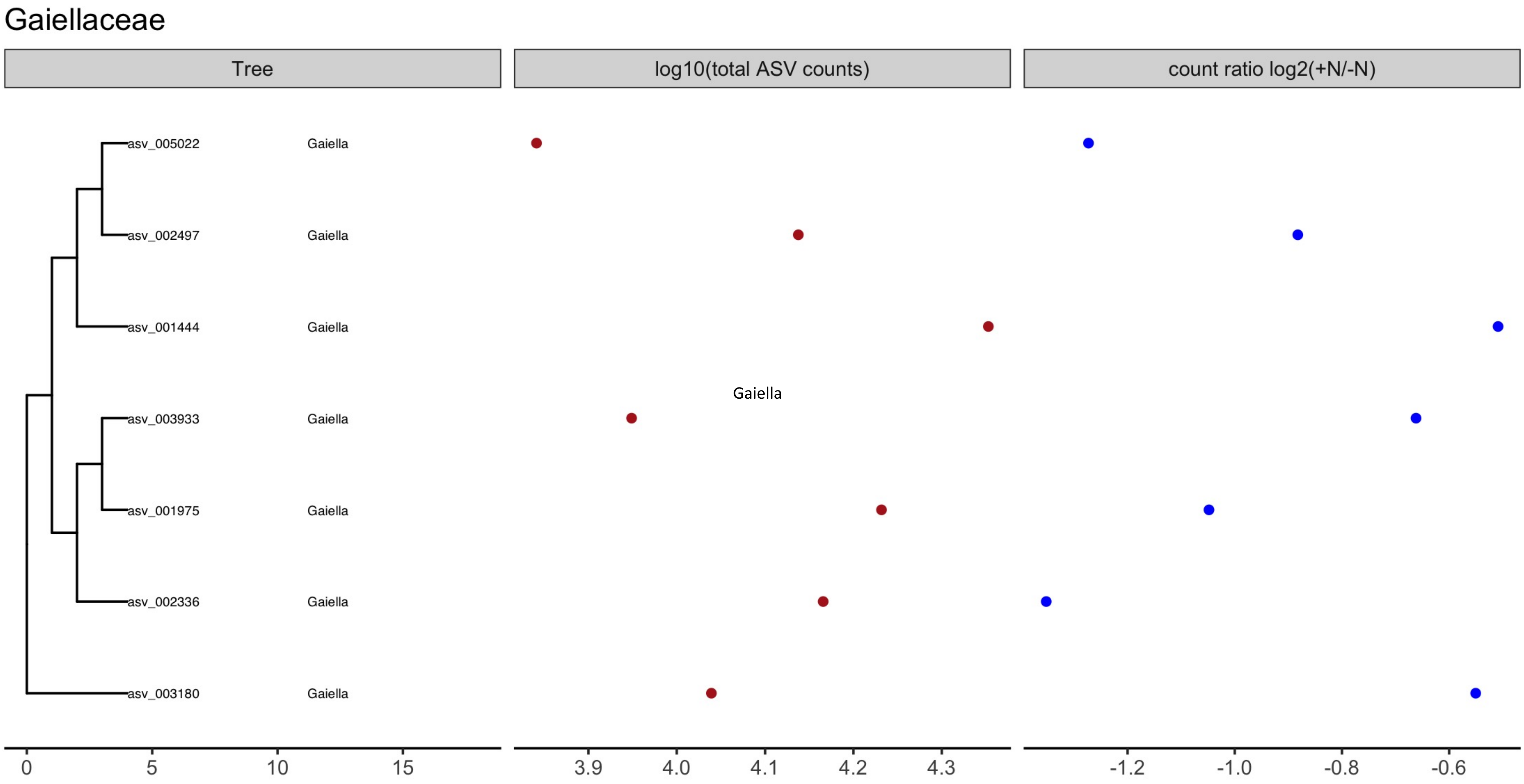
Gaiellaceae
Tree
log10(total ASV counts)
count ratio log2(+N/-N)
asv_005022
asv_002497
asv_001444
asv_003933
asv_001975
asv_002336
asv_003180
Gaiella
Gaiella
Gaiella
Gaiella
Gaiella
Gaiella
Gaiella
Gaiella
0
5
10
15
3.9
4.0
4.1
4.2
4.3
-1.2
-1.0
-0.8
-0.6

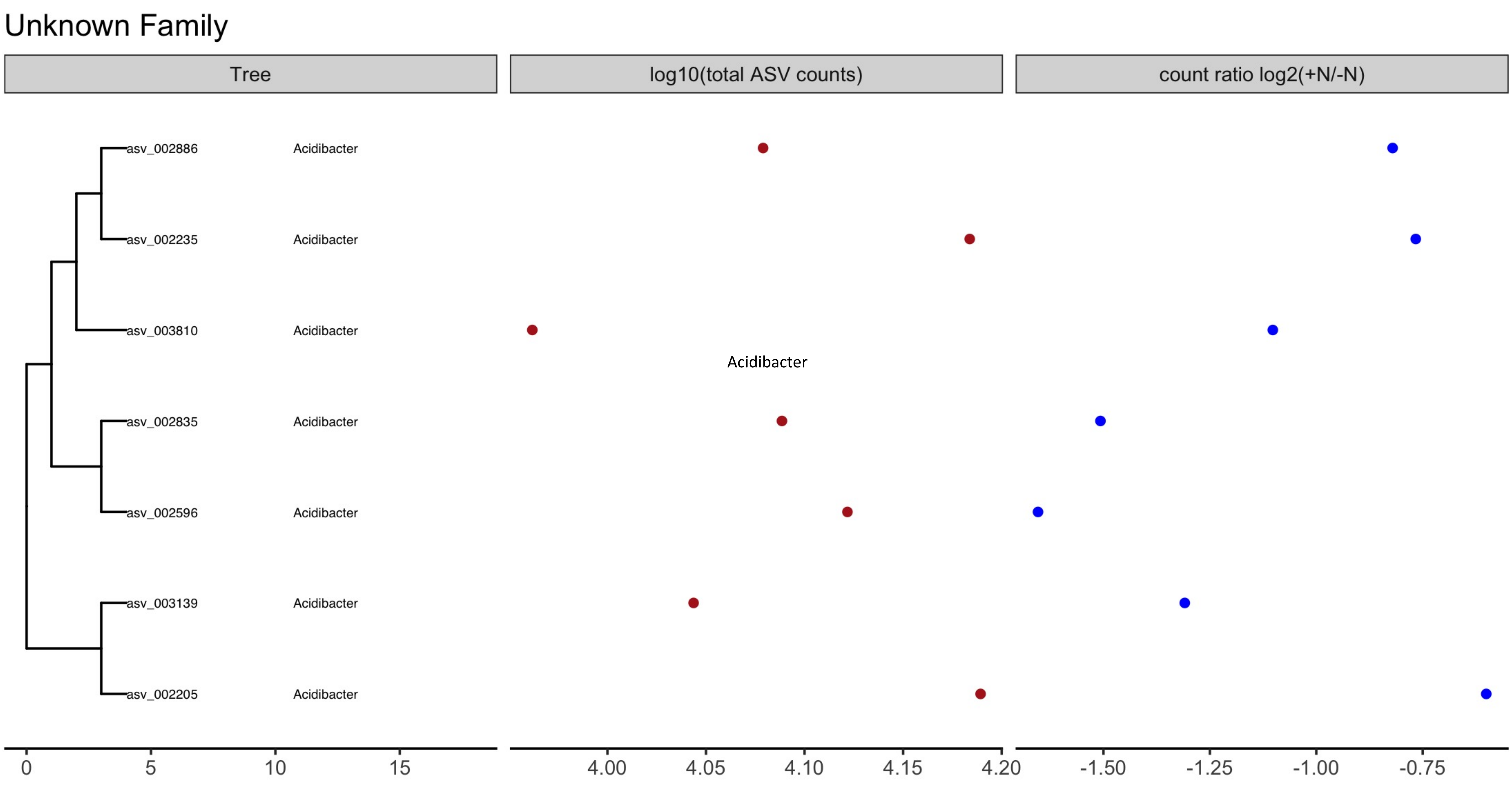

Unknown Family
Tree
log10(total ASV counts)
count ratio log2(+N/-N)
asv_002886
asv_002235
asv_003810
asv_002835
asv_002596
asv_003139
asv_002205
Acidibacter
Acidibacter
Acidibacter
Acidibacter
Acidibacter
Acidibacter
Acidibacter
Acidibacter
0
5
10
15
4.00
4.05
4.10
4.15
4.20
-1.50
-1.25
-1.00
-0.75

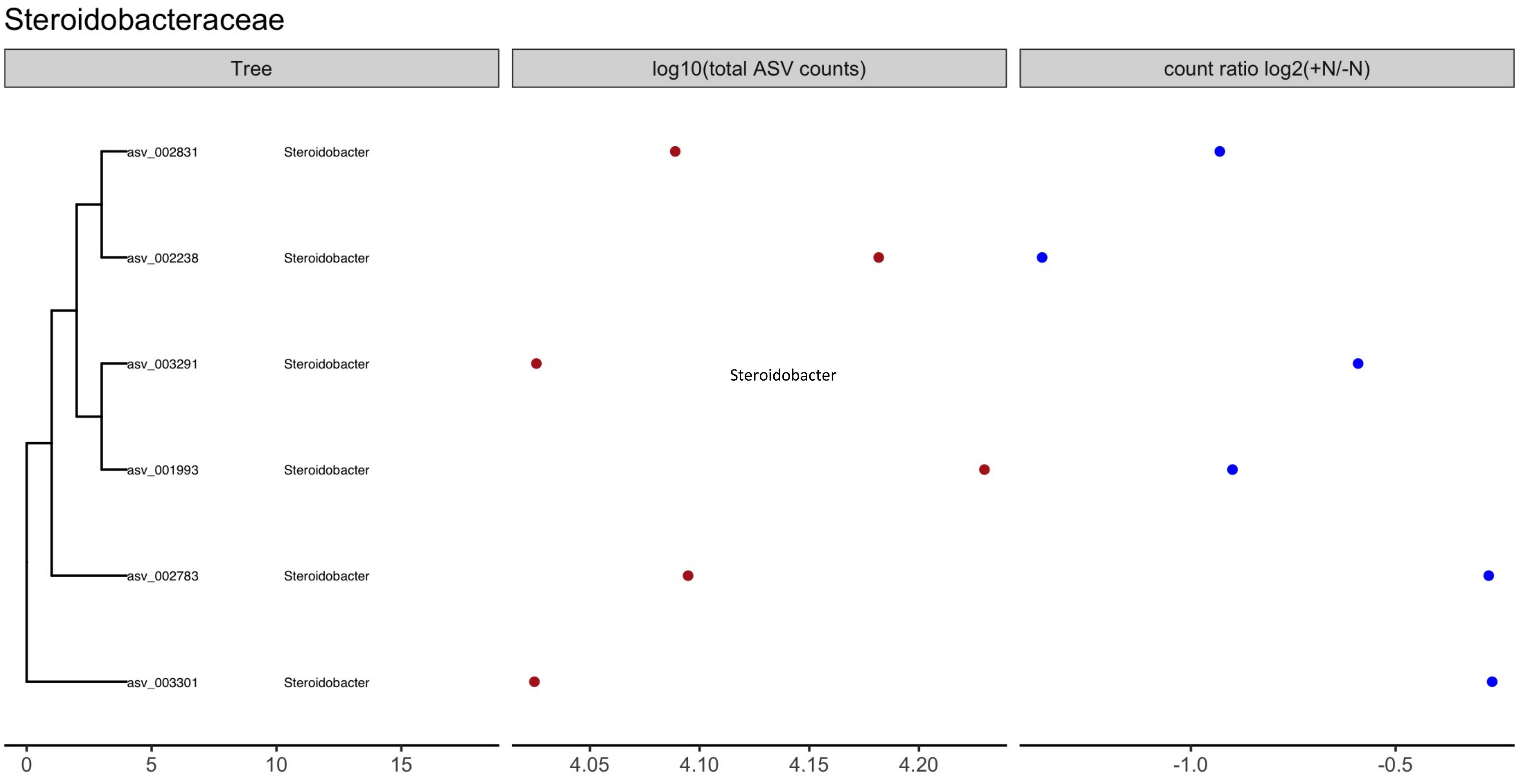
Steroidobacteraceae
Tree
log10(total ASV counts)
count ratio log2(+N/-N)
asv_002831
Steroidobacter
asv_002238
Steroidobacter
asv_003291
Steroidobacter
asv_001993
Steroidobacter
asv_002783
Steroidobacter
asv_003301
Steroidobacter
Steroidobacter
0
5
10
15
4.05
4.10
4.15
4.20
-1.0
-0.5

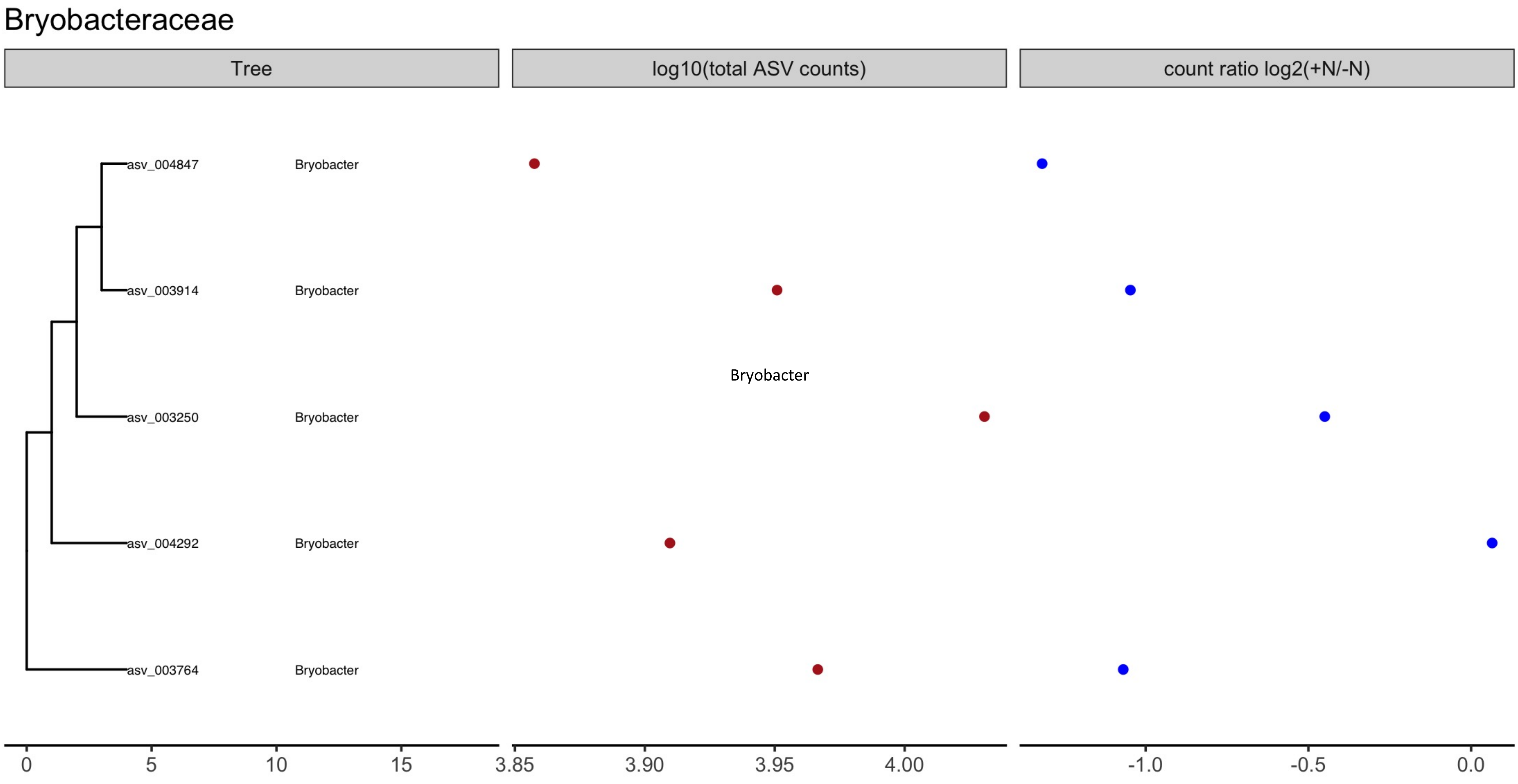
Bryobacteraceae
Tree
log10(total ASV counts)
count ratio log2(+N/-N)
asv_004847
Bryobacter
asv_003914
Bryobacter
Bryobacter
asv_003250
Bryobacter
asv_004292
Bryobacter
asv_003764
Bryobacter
0
5
10
15
3.85
3.90
3.95
4.00
-1.0
-0.5
0.0

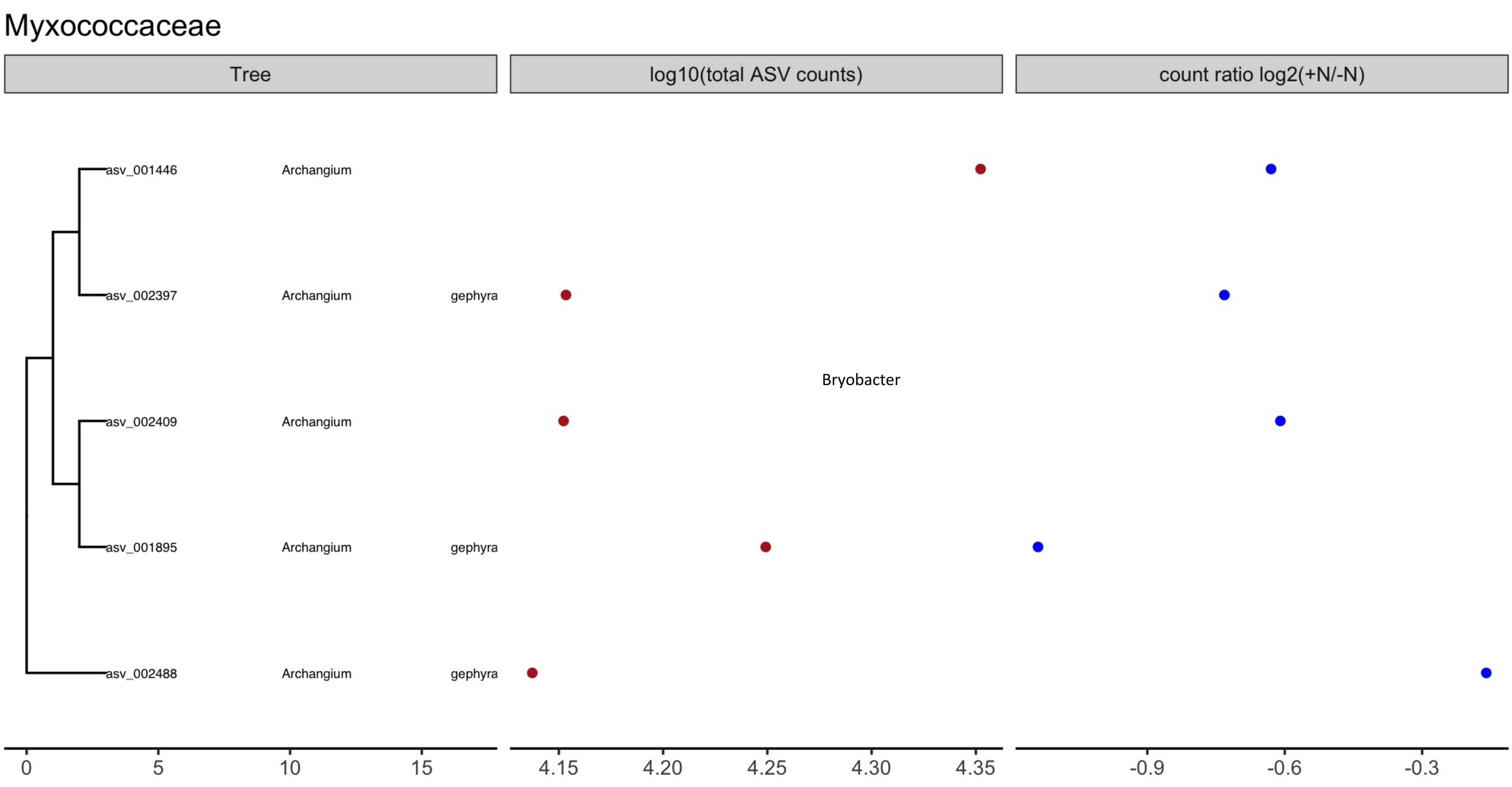

Myxococcaceae
Tree
log10(total ASV counts)
count ratio log2(+N/-N)
asv_001446
Archangium
asv_002397
Archangium
gephyra
asv_002409
Archangium
asv_001895
Archangium
gephyra
asv_002488
Archangium
gephyra
Bryobacter
0
5
10
15
4.15
4.20
4.25
4.30
4.35
-0.9
-0.6
-0.3

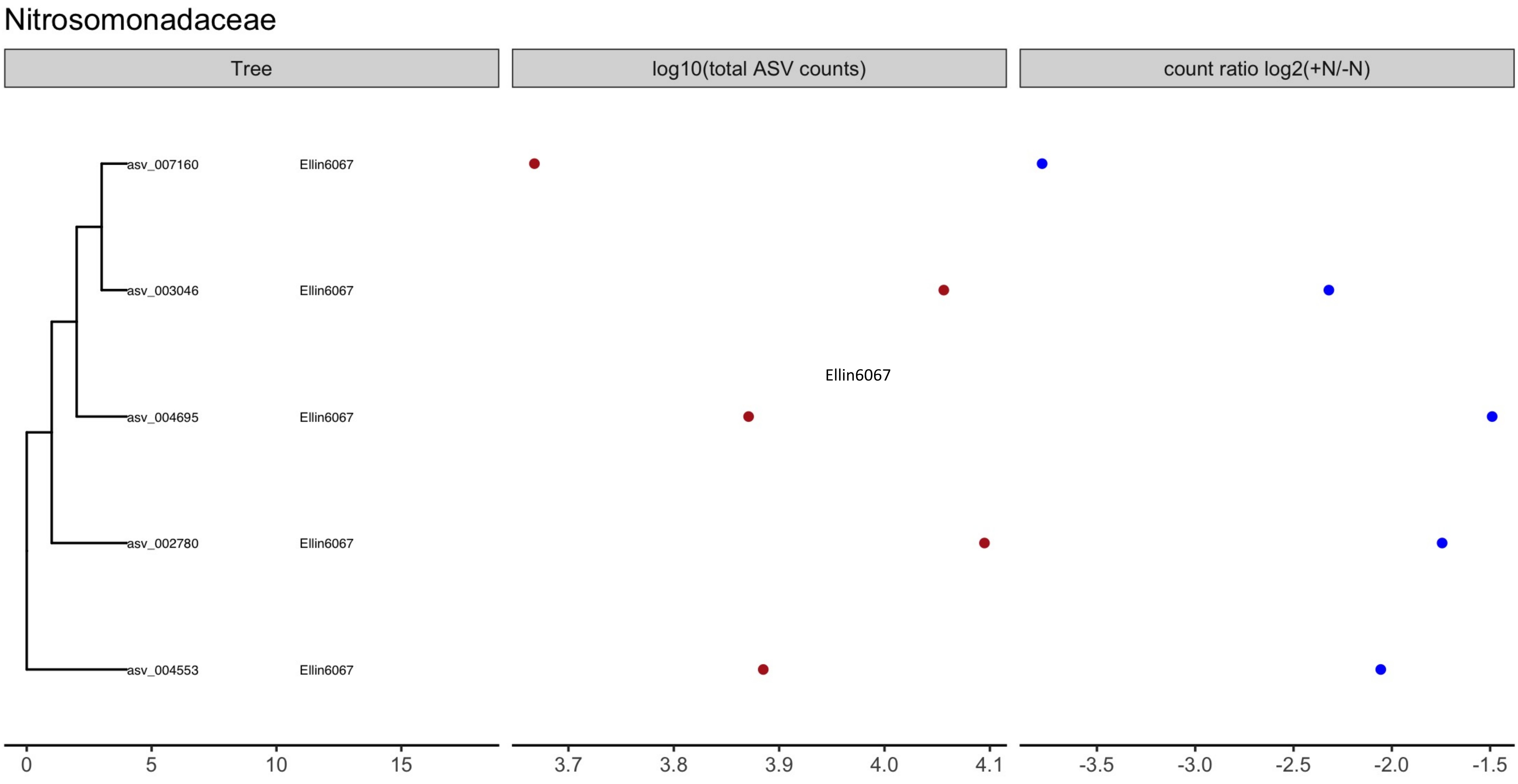

Nitrosomonadaceae
Tree
log10(total ASV counts)
count ratio log2(+N/-N)
asv_007160
Ellin6067
asv_003046
Ellin6067
Ellin6067
asv_004695
Ellin6067
asv_002780
Ellin6067
asv_004553
Ellin6067
0
5
10
15
3.7
3.8
3.9
4.0
4.1
-3.5
-3.0
-2.5
-2.0
-1.5
